# Chemistry of Carbon-Substituted Derivatives of Cobalt Bis(dicarbollide)(1^−^) Ion and Recent Progress in Boron Substitution †

**DOI:** 10.3390/molecules28196971

**Published:** 2023-10-07

**Authors:** Lucia Pazderová, Ece Zeynep Tüzün, Dmytro Bavol, Miroslava Litecká, Lukáš Fojt, Bohumír Grűner

**Affiliations:** 1 Institute of Inorganic Chemistry of the Czech Academy of Sciences, 250 68 Řež, Czech Republic; pazderova@iic.cas.cz (L.P.); tuzun@iic.cas.cz (E.Z.T.); bavol@iic.cas.cz (D.B.); litecka@iic.cas.cz (M.L.); 2 Department of Inorganic Chemistry, Faculty of Natural Science, Charles University, Hlavova 2030/8, 128 43 Prague, Czech Republic; 3 Institute of Biophysics of the Czech Academy of Sciences, Královopolská 135, 612 00 Brno, Czech Republic; fojtlukas@gmail.com

**Keywords:** borane, carborane, dicarbollide, cobalt bis(dicarbollide), lithiation, metalation

## Abstract

The cobalt bis(dicarbollide)(1^−^) anion (**1^−^**), [(1,2-C_2_B_9_H_11_)_2_-3,3′-Co(III)](1^−^), plays an increasingly important role in material science and medicine due to its high chemical stability, 3D shape, aromaticity, diamagnetic character, ability to penetrate cells, and low cytotoxicity. A key factor enabling the incorporation of this ion into larger organic molecules, biomolecules, and materials, as well as its capacity for “tuning” interactions with therapeutic targets, is the availability of synthetic routes that enable easy modifications with a wide selection of functional groups. Regarding the modification of the dicarbollide cage, syntheses leading to substitutions on boron atoms are better established. These methods primarily involve ring cleavage of the ether rings in species containing an oxonium oxygen atom connected to the B(8) site. These pathways are accessible with a broad range of nucleophiles. In contrast, the chemistry on carbon vertices has remained less elaborated over the previous decades due to a lack of reliable methods that permit direct and straightforward cage modifications. In this review, we present a survey of methods based on metalation reactions on the acidic C-H vertices, followed by reactions with electrophiles, which have gained importance in only the last decade. These methods now represent the primary trends in the modifications of cage carbon atoms. We discuss the scope of currently available approaches, along with the stereochemistry of reactions, chirality of some products, available types of functional groups, and their applications in designing unconventional drugs. This content is complemented with a report of the progress in physicochemical and biological studies on the parent cobalt bis(dicarbollide) ion and also includes an overview of recent syntheses and emerging applications of boron-substituted compounds.

## 1. Introduction

The 3,3-*commo-closo*-bis(-1,2-dicarba-3-cobalt dodecaborane)(**1^−^**) anion, often abbreviated using the trivial name “cobalt bis(dicarbollide) anion” or “COSAN” (**1^−^**), was discovered through the pioneering work of M.F. Hawthorne in 1965. M. F. Hawthorne found that the open face of the deprotonated 7,8-dicarba-nido-undecaborate ion (dicarbollide ion) [C_2_B_9_H_11_]^2−^ is iso-electronic with the cyclopentadiene ion, providing six electrons for bonding with a transition metal in a η^5^-fashion, similar to organic metallocenes [1,2]. Indeed, the cobalt bis(dicarbollide) ion possesses fully occupied bonding inner orbitals with an 18-electron structure, akin to organometallic ferrocene, making it the most stable compound within the series of metalla bis(dicarbollide) ions. However, significant differences arise, starting with Co(III) as the central atom, its oxidation state, ionic charge of the cluster, 3D architecture, overall size, presence of B-H and C-H sites in the molecule, and dipole moments. This compound can be synthesized through the deboronation of ortho-carborane, followed by deprotonation and treatment with cobalt chloride [2,3,4,5].

The chemistry of this ion has consistently garnered interest and, consequently, has been the subject of constant interest and has been the topic of several previous reviews and book chapters by, for example, Bregadze and Sivaev [6], Dash and Hosmane [7], and Grimes [8]. Its unique electronic structure, characterized by the presence of hydridic BH vertices, charge delocalization throughout the structure, and aromaticity, contributes to its high stability. The ion is marked by its ionic negative charge, diamagnetic character, low nucleophilicity, and very high thermal, chemical, and radiolytic stability. Its versatility is evident in various applications, including nuclear waste treatment [9,10], conducting polymers and coordination polymers [11], electrochemistry, ion-selective electrodes [11,12,13,14], catalysts, photocatalysts [15,16], and more. The ion (**1^−^**) also exhibits hydrogen and dihydrogen bonding capabilities, which facilitate self-assembly [17,18,19], water solubility [20], and the formation of micelles and vesicles [21,22,23]. Moreover, it shows resistance to catabolism [24], easy penetration into cells, low toxicity in both in vitro and in vivo models [25,26,27,28,29], and the potential for unconventional types of interactions [30]. Therefore, this ion represents an attractive candidate for medicinal applications, such as inhibitors of therapeutically relevant enzymes like HIV Protease [31], cancer-associated Carbonic Anhydrase IX (CA-IX) [32], NO synthase [33], kinases [34], antibacterial drugs [35,36], and antimycotic agents [37], as well as a boron delivery platform for Boron Neutron Capture Therapy (BNCT) of cancer [38], modifications and labeling of DNA [39], and the development of electrochemical, photoredox, and radiochemical probes in diagnostics [24,40,41,42,43].

The derivatization of the cobalt bis(dicarbollide) anion can be achieved at both the carbon and boron sites of the cluster. However, the synthetic methods for these derivatives differ [6,7,8]. There has been greater emphasis on studying substitution at the boron atoms due to the availability of convenient synthetic pathways, generally proceeding via the Electrophile Induced Nucleophilic Substitution (EINS) mechanism [8]. Functionalization at the carbon atoms has been accessible only through indirect synthetic routes involving metal insertion into modified dicarbollide anions as ligands. In contrast to dicarba-*closo*-dodecaboranes (carboranes) [44] and the 1-carba*-closo*-dodecaborane ion [45], the CH sites in the cobalt bis(dicarbollide) anion appear less susceptible to metalation reactions, and, thus, direct modifications of carbon became available much later, generally over the course of the last decade. Although some early reports of successful lithiation can be found in the literature [46], reproducibility under the described reaction conditions was poor or rather inaccessible. Therefore, chemists working in this area almost lost hope, after many unsuccessful attempts, that direct synthesis via metalation and reactions with electrophiles could be a feasible method for the successful derivatization of the cage.

Interest was reborn after the first successful report on the synthesis of compounds containing carbon–silicon and carbon–phosphorus bonds by the group of Teixidor and Viñas [47,48,49]. Following the development of approaches and reaction conditions allowing for reliable lithiation and subsequent reactions, the direct synthesis of carbon-modified cobalt bis(dicarbollide) anions containing carbon–carbon, carbon–silicon, carbon–phosphorus or carbon–halogen bonds, and various terminal groups became accessible, forming the main focus of this review. Since the last comprehensive review on cobalt bis(dicarbollide) derivatives in 2017, when Dash and Hosmane et al. [7] published an overview of cobalt bis(dicarbollide) derivatives functionalized on both carbon and boron atoms, significant progress has been made in this field. The purpose of our review is, therefore, to summarize the main achievements in cobalt bis(dicarbollide) ion chemistry published since the last review and to provide a comprehensive overview of the developments in this area over recent years.

## 2. General Properties of the Cobalt Bis(dicarbollide) Ion

Cobalt bis(dicarbollide) is a low-spin diamagnetic (d^6^) anionic complex composed of a Co(III) atom at its center, coordinated to two [C_2_B_9_H_11_]^2−^ ligands. The electrons are fully delocalized in the inner bonding orbitals, and the ion is considered an aromatic species [50,51,52,53]. While two known isomeric forms exist, [(1,2-C_2_B_9_H_11_)_2_-3,3′-Co(III)]^−^ and [(1,7-C_2_B_9_H_11_)_2_-2,2′-Co(III)]^−^, the number of potential isomers can reach 45 [9]. The latter isomer, with non-adjacent carbon atoms in the open {C_2_B_3_} face of the dicarbollide ligands, corresponds to the thermodynamically more stable species [54]. Isomerization into the meta-isomer requires high energy and temperature, but the presence of substituents may reduce the activation barrier for isomerization [8].

**Figure 1:**
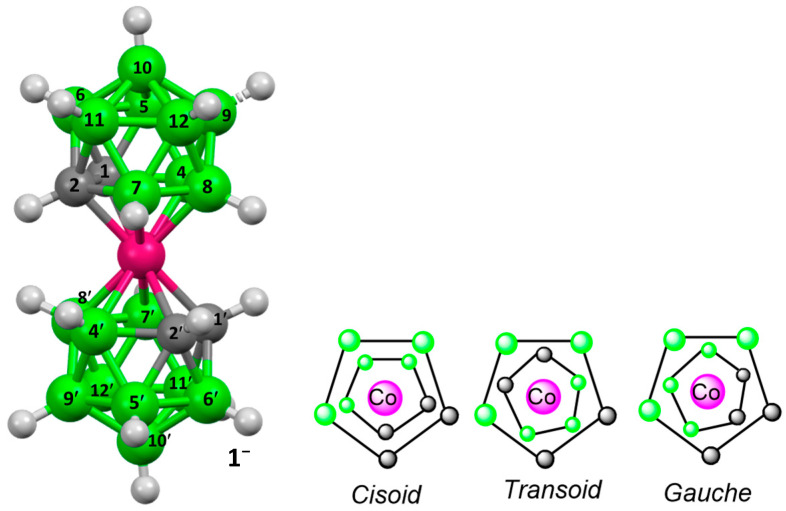
Schematic presentation of the molecular structure of cobalt bis(dicarbollide) ion (**1^−^**) (from XRD structure of Cs^+^ salt) presented in ref. [61] and three main energetically favored conformations [54]. Boron atoms are drawn in green, carbon in gray, and cobalt in rose color.

The structure of the cobalt bis(dicarbollide) ion resembles a rod-like “peanut” shape with nanometer dimensions. The distance between terminal H(10) atoms in apical positions is approximately 1.2 nm long, and the cage is 0.72 nm wide [32]. Both dicarbollide ligands can rotate around the *z*-axis in a solution. The surface is covered with B-H bonds, with partial negative charges localized on the hydrogen atoms. Four C-H groups are slightly acidic, though however, the determination of the corresponding p*K*_a_ values is pending. XRD structures and chemical calculations indicate the presence of several discrete conformers in solid-state, including *transoid*-(*staggered*), *gauche*-(*gauche*), and *cisoid*-(*eclipsed*) geometries as the main energetically favorable forms (Figure 1). The fast rotation of both ligand planes around the central cobalt atom occurs in a solution. Density Functional Theory (DFT) calculations suggest that the energetic separation of the three rotamers, around 11 kJ mol^−1^, and barriers for their interconversion, approximately 41 kJ mol^−1^ [54,55], are low. The rapid rotation of ligand planes in metalla bis(dicarbollide) ions is also supported by experiments; the most recent paper on a similar iron sandwich compound was published in 2022 [56]. Low energy interconversion precludes the isolation of the rotamers from the solution, however, these can be seen in solid state structures. The Cs^+^ salt of the parent ion typically adopts a *transoid* conformation in solid-state structures, although structures of many other salts with alkyl ammonium cations in the Cambridge Structural Database (CCDC) show a *cisoid* arrangement. Most derivatives of ion **1^−^** in the CCDC usually adopt *cisoid* or *gauche* conformations with a wide variety of torsion angles. Only derivatives with heavier halogens and those heterosubstituted with Ph and I adopt a *transoid* conformation, attributed to the formation of B-H-Hal or B-H-Ar bonds [57,58]. As reported in the literature, a search of the CCDC Database revealed that approximately 80% of the deposited structures adopt a *cisoid* conformation, 17% are *gauche*, and only 5% are *transoid* arrangements of the carbon atoms [59]. Conformational mobility can be partly or fully restrained by the presence of two (or more) bulky substituents on the ligand planes sandwiching the cobalt ion [60] or due to a bridge substituent (*ansa*-substitution) interlinking the boron or carbon positions.

The cobalt bis(dicarbollide) ion has a high surface area to charge ratio, with maximum electron density located on the B(8), B(9), and B(12) skeletal vertices [61,62]. No recent computational analysis had been reported until 2005 [54]. A DFT analysis showed that the electronegativity difference of B and C increases the electron density around the carbons of the dicarbollide ligand, and the Highest Occupied Molecular Orbital (HOMO) of cobalt bis(dicarbollide) exhibits the expected predominant d(metal) and pronounced ligand character, respectively [54].

Cobalt bis(dicarbollide) can be reduced to its Co(II) dianion by sodium amalgam or metallic cesium and, subsequently, oxidized back into its Co(III) state [46]. Its ability to undergo a Co(II/III) redox process makes it suitable for use in electrochemical and electronic applications [63]. Its ability to undergo a reversible 1e^−^ oxidation 1e^−^ reduction cycle was first reported in 1971, suggesting an Electron transfer, Chemical reaction, and Electron transfer (ECE-type) mechanism supported by cyclic voltammetry [64].

^11^B NMR is a comprehensive method for the identification of B and C-substituted carboranes and cobaltacarboranes. The paramagnetic Co(II) complex [Co(C_2_B_9_H_11_)_2_]^2−^ has a wide ^11^B NMR range (from +132 to −66 ppm), while the diamagnetic Co(III) complex has peaks typically in the +6 to −23 ppm region [46]. The sp^n^ hybridization of dicarbollide ligands is expected to result in larger coupling constants compared to ligands of pure p-character in their bonding orbitals, such as in a {C_5_H_5_}^−^ ion [65]. Despite distinguishable B(8) substitution, signals of the 18 BH vertices can often overlap, particularly those observed for B(4, 7, 9, and 12), which may complicate the assignment under practical circumstances. Bűhl et al. demonstrated that the ^11^B NMR shifts in the spectra of cobalt bis(dicarbollide) and its derivatives can be predicted using DFT calculations within a relatively small deviation [54]. Theoretical nucleus-induced chemical shift (NICS) studies showed that cobalt bis(dicarbollide) derivatives possess high aromaticity, providing excellent stability and reactivity [50].

## 3. Carbon-Substituted Derivatives

In the chemistry of the neutral 1,2-dicarba-*closo*-dodecaborane, it is well-established that the properties of functional groups on carbon and boron atoms can differ significantly. For instance, the p*K*_a_ of functions such as NH_2_, SH, and COOH, as example, attached to C(1) and B(9) positions in 1,2-dicarba-*closo*-dodecaborate can vary appreciably [66]. In general, groups on carbon atoms are notably more acidic than those bound to boron. A similar trend can be expected for groups on B and C atoms of the cobalt bis(dicarbollide) cage, although a systematic and sufficiently quantitative set of physicochemical data is not yet available.

Concerning amine derivatives, optimized structures and relative proton affinities have been calculated based on B3LYP and BP86 functionals along with conformational changes using BP86/6-31G* quantum-chemical computations [67]. The calculations were performed over the whole series of seven isomeric C(1), B(4), B(5), B(6), B(8), B(9), and B(10) substituted amines in *transoid*-conformation, as well as 11 possible isomers of C(1), C(2), B(4), B(5), B(6), B(7), B(8), B(9), B(10), B(11), and B(12) -NH_2_ derivatives of the cobalt bis(dicarbollide) ion. The results clearly demonstrate a significant increase in the acidity of amino functions when present on carbon atoms. However, in a *transoid*-conformation, the B(6)-NH_2_ group appears more acidic than the C(1)-NH_2_ group. Conversely, when considering *cisoid*-arrangements, the latter becomes the most acidic. The energy difference between proton affinity in *cisoid*-conformation is 178 kJ mol^−1^ between C(1)-NH_2_ and the most basic B(8)-NH_2_.

**Figure 2:**
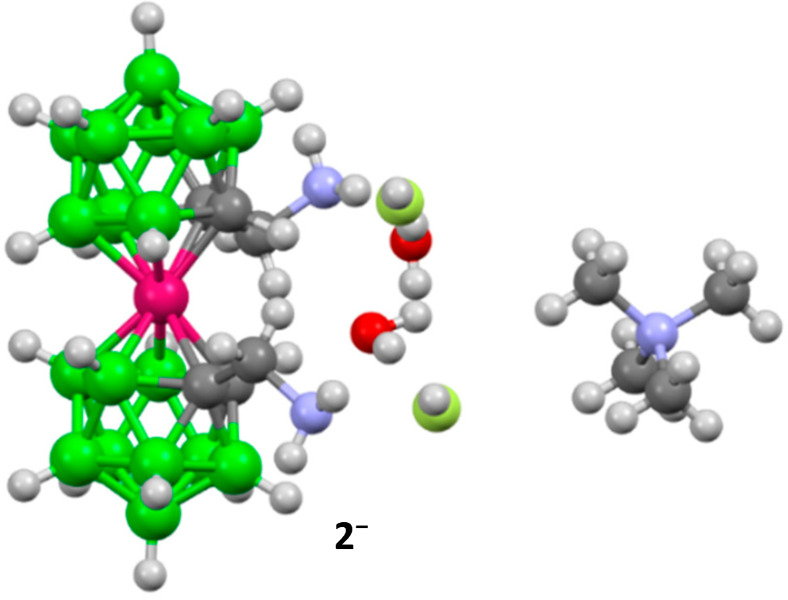
Schematic presentation of the molecular structure of the double salt of the formulation [1,1′-(HCl·H_2_N-CH_2_-C_2_B_9_H_10_)_2_-3,3′-Co(III)]Me_4_N based on unpublished structural data [68]. Boron atoms are drawn in green, carbon in gray, cobalt in rose, chlorine in green-yellow, nitrogen in blue, and oxygen in red color.

Available experimental evidence supports this theory and shows that NH_2_ groups bonded to boron atoms readily undergo protonation to -NH_3_ under slightly acidic conditions or even at or below pH 7, producing stable zwitterionic compounds. However, the protonation of C-substituted amines with 3 M HCl proceeds differently and results in the respective hydrochlorides -NH_2_·HCl instead, which is characterized by the formation of double salts. This feature has also been observed in several unpublished solid-state structures of amines that have recently been crystallized [68]; one example being [1,1′-(HCl·H_2_N-CH_2_-C_2_B_9_H_10_)_2_-3,3′-Co(III)]Me_4_N (**2^−^**), schematically depicted in Figure 2. As a practical consequence, these compounds tend to behave as anions in solution.

Recent demands for fine-tuning interactions of cobalt bis(dicarbollide) derivatives with biological targets in medicinal chemistry have sparked increased interest in the synthesis and use of carbon-substituted derivatives as a viable solution, offering better control over the distance between functional groups and the cage. Indeed, C-substitution allows for the introduction of one or two substitution groups, which may be identical, or eventually different. When combined with boron substitution, this opens up a broad range of geometrical and functional possibilities. In practical terms, these derivatives have proven advantageous, for example, in the design of CA-IX inhibitors, demonstrating clear benefits which proved to have clearly better properties compared to boron-substituted derivatives, particularly in terms of greater flexibility in modifications and improved inhibitory properties (as discussed in Section 5.2.1 below) [32].

### 3.1. Indirect Substitutions via Modified 1,2-Dicarba-closo-dodecaborate

Indirect substitution pathways were extensively covered in previous reviews and books [6,7,8]. The first known methods for obtaining C-substituted derivatives involved the modifications of *ortho*-carborane followed by the degradation of specific derivatives into modified dicarbollide ions. This was followed by deprotonation with a strong base (typically NaH or KO*^t^*Bu) in THF or DME and metal insertion via a reaction with anhydrous CoCl_2_. Generally, this approach primarily leads to disubstituted compounds, typically obtained as a mixture of two diastereoisomers. The first is represented by the asymmetric (*racemic*) form, and the second corresponds to the form that has a plane of symmetry (*meso*) (see Section 3.3 on stereochemistry below). The substituents are then located in two ligand planes up and down the cobalt atom. This method can also be employed in the synthesis of monosubstituted compounds, provided that a mixture of modified and parent dicarbollide ligand is reacted with cobalt(II) chloride (or other Co(II) salts). However, its use is quite uneconomical since the reaction kinetics of the parent dicarbollide ion is faster than that of the substituted ligand. Therefore, a considerable amount of the parent sandwich ion **1^−^** forms in these reactions, along with some disubstituted products that start to form when the parent dicarbollide ion is spent.

**Figure 3:**
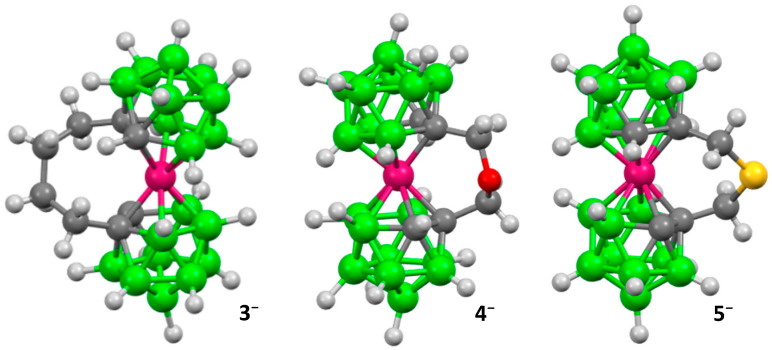
Schematic presentation of the molecular structures of (**3^−^**) *rac*-form of the butylene bridged derivative [70], (**4^−^**) *rac*-CH_2_OCH_2_- bridged [71], and (**5^−^**) *meso*-thioether bridged [72] cobalt bis(dicarbollide). Boron atoms are drawn in green, carbon in gray, cobalt in rose, sulfur in yellow, and oxygen in red color.

Derivatives substituted on carbon atoms with two or four alkyls or phenyls were prepared using this approach. Additionally, a series of bridged *ansa*-derivatives were synthesized, where two carboranes or dicarbollide anions were interconnected via a chain, eventually containing heteroatoms, into which a cobalt atom was inserted (Figure 3). This category also includes Hawthorne’s famous *ansa*-pyrazolyl Venus flytrap system designed for easy labeling with ^60^Co(III) for radioimaging [69]. In addition, Hawthorne’s group also synthesized bridged derivatives on cage carbons of cobalt bis(dicarbollide) with -propyl-, -butyl-, -pentyl-, and -TosN(CH_2_CH_2_) spacers via deboronation, deprotonation, and cobalt insertion steps with yields ranging from 21% to 61% [70]. They also similarly prepared -COC- and -CSC- bridged derivatives, with the latter being oxidizable into its corresponding sulfone derivative using meta-chloroperoxybenzoic acid in dichloromethane [71,72]. More recent studies by Nabakka et al. and Viñas et al. focused on preparing cobalt bis(dicarbollide) substituted with ethylene glycol units, which proved advantageous in partitioning ^137^Cs, ^90^Sr, and ^152^Eu from simulated nuclear waste [72,73,74]. Recently, carbon-substituted dimethylsulfanyl derivatives [60] and some alkyl-sulfonamides alkylsulfonamides [75] were prepared using the indirect approach.

### 3.2. Direct Modifications

Since the cesium salt of cobalt bis(dicarbollide) has become widely available commercially, direct lithiation reactions offer advantages over the above indirect methods by allowing the synthesis of either mono- or disubstituted compounds in a single step. However, it is worth noting that the metalation with RLi requires strictly controlled low-temperature conditions, although the exact reason for this requirement remains unclear. Considering similar, but neutral, ferrocene, the acidity of the C-H group is significantly enhanced compared to common aromatic molecules, allowing for easy lithiation [76]. In the case of cobalt bis(dicarbollide), a similar effect can probably be anticipated, however, corresponding systematic studies are not yet available.

**Figure 4:**
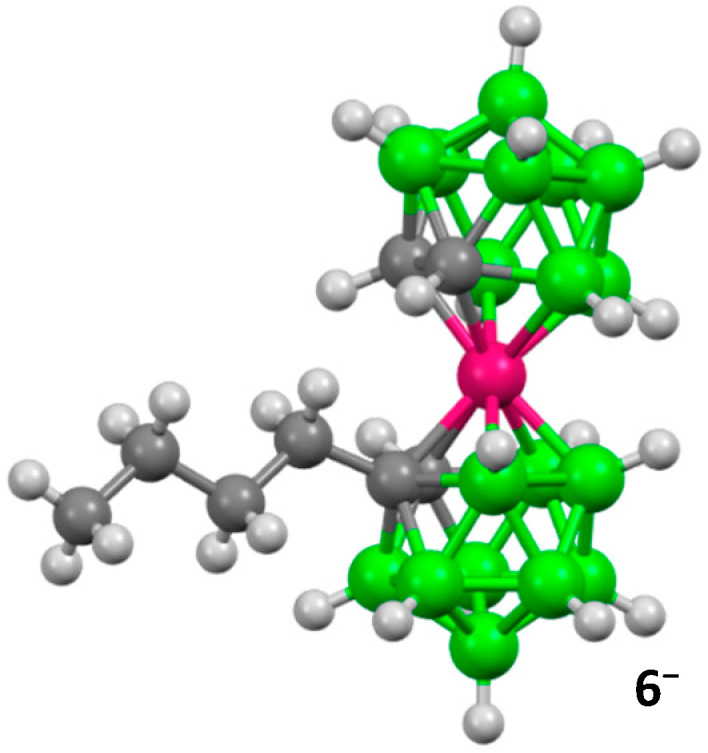
Drawing of the molecular structure of the butyl-substituted derivative (**6^−^**) based on XRD analysis [68]. Boron atomsare drawn in green, carbon in gray, and cobalt in rose color.

It is currently uncertain as to what extent C-Li interactions display an ionic character, as seen in the case of the [CB_11_H_12_]^−^ ion, which has been shown to stabilize a “naked” Li^+^ cation in a solution [77]. Complications in reacting lithiated species could be connected with the abstraction of Li^+^ by other species present in the solution, the aggregation of the RLi reagent depending on the specific solvent [78], or unspecific interactions of Li^+^ complexes in a solution with other sites on the boron cage. Some observations indicate that C-Li vertices can, under certain reaction conditions, follow unexpected pathways, such as nucleophilic reactions with R present in RLi, resulting in C-R substitution [68]. An example of a 1-butyl derivative isolated from a reaction with BuLi in DME is presented in Figure 4. Other examples include nucleophilic substitution on a carbonyl group observed in reactions with *N*-(ω-bromoalkyl)phthalimides (see Section 3.7.3 below) [79] or the preferential formation of a C-Br bond in a reaction with BrCN (see Section 3.10) [80].

On the other hand, metalation, and subsequent reactions with electrophiles, offer advantages in terms of direct and rapid syntheses, the possibility of generating monosubstituted compounds, and the capability to introduce substituents that would interfere during metal insertion (due to coordination to cobalt), which may react with dicarbollide ligands or decompose under the highly alkaline conditions that are necessary when using indirect methods.

### 3.3. Stereochemistry of Substitution

In principle, there are four CH vertices available for metalation in cobalt bis(dicarbollide) ions. However, experimental trials and compounds reported in the literature [46,81] suggest that it is challenging to achieve more than disubstituted products, even when using a larger excess (up to six equivalents) of RLi and an appropriate ratio of reagents. Although small quantities, up to approximately 20%, of tri- and tetra-substituted species may form, these reactions do not typically go to completion. Several factors could contribute to this limitation, including the steric bulk of the Li^+^ (Solvent)_x_ moiety on the carbon vertex, the potential involvement of B-H interactions, or electronic repulsion of the lithiated C-vertices, which carry a partial negative charge.

**Figure 5:**
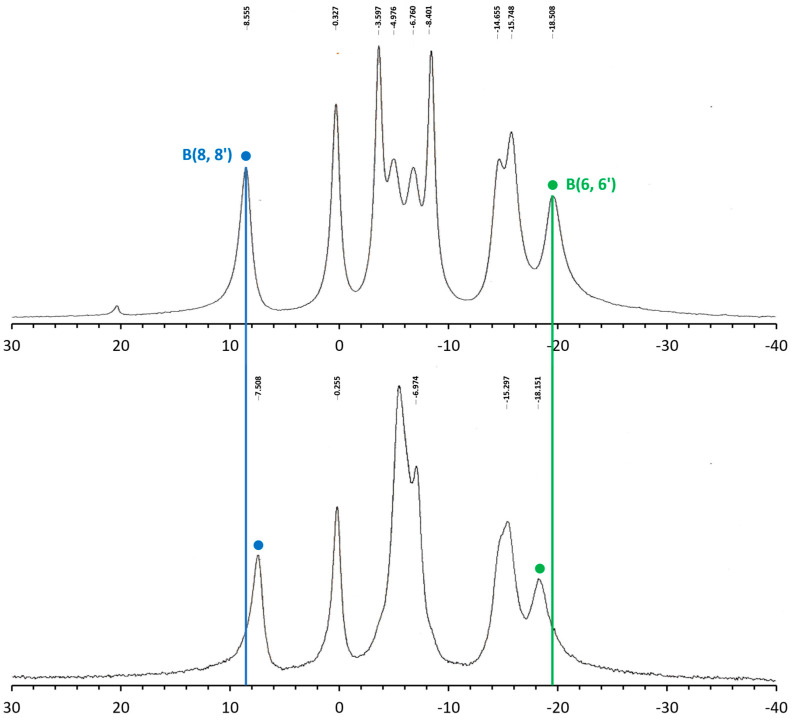
^11^B {^1^H} NMR spectra of *rac*- (up) and *meso*-forms (down) of disulfonamidoethyl of **1^−^**.

**Scheme 1:**
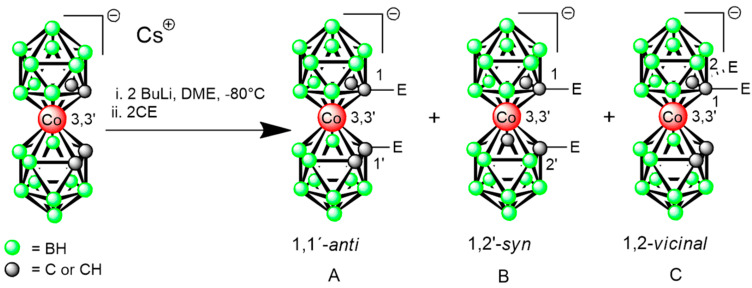
Possible stereoisomers of carbon disubstituted species, E means a general substituent bind by simple bond. (**A**) *meso*-, (**B**) *rac*-, and (**C**) *vicinal*-isomer.

In contrast to ferrocene chemistry [79], the *ortho*-metalation on the cobalt bis(dicarbollide) ion appears to be difficult, at least based on current knowledge. Indirect experimental evidence indicates that, in cases of disubstitution, typically two nonadjacent vertices are metalated and subsequently substituted, resulting in the formation of one or two diastereoisomers out of three possible options (Figure 5). The exact outcome depends (Scheme 1) on the reagent and reaction conditions. Most often, a mixture of *rac*- and *meso*-forms is obtained. Typically, the asymmetric *rac*-form, characterized by the highest spatial separation of the substituted carbon atoms, is the most abundant product. From a study published by Juarez-Perez et al. on the silylation of carbon vertices, it follows that the presence of the *meso*-form can increase with temperature [48]. Solvent choice can also favor the prevalence of *rac*-form, e.g., the replacement of dimethoxy ethane (DME) with diethoxy ethane (DEE) led to a higher ratio of the *rac*-form [80,81]. Some reagents, such as CO_2_, CH_3_OCH_2_CH_2_OCH_2_Cl (MEMCl) [46] in reaction with lithiated parent cobalt bis(dicarbollide), or trimethylene oxide, in case of the ion **1^−^** rigidified with an oxygen bridge, produced essentially pure *rac*- isomer [82]. This is likely due to increased steric strain around the reaction sites induced by the coordination of Li^+^ to a specific solvent, or steric requirements of the initial substituent entering the carbon site and/or its coordination ability. In the latter case, the preference was connected with the inherent *cisoid*-arrangement of carbons in the bridged molecule [82], which apparently resulted in higher steric strain around the carbon atoms.

The presence of two (or three) isomers can be discerned from the ^11^B, ^1^H, and ^13^C spectra [46,81]. An example of differences in ^11^B NMR signals between *rac*- and *meso*-forms is illustrated in Figure 5. It can be seen that both forms exhibit very similar spectral patterns with the same number of nine boron signals. Only the peaks in the lowest and highest field corresponding to B(8,8′) and B(6,6′) may be, depending on the specific substituent, mutually and distinguishably shifted. Other ^11^B signals for particular boron vertices exhibit only minor differences in chemical shifts. These signals are often overlapping, and overlap especially in the analysis of unseparated mixtures of diastereoisomers.

The pseudosymmetric spectral pattern in the ^11^B -NMR of the *rac*-form is notably unexpected. Even so, the spectrum reflects the asymmetry of each ligand but corresponds to the magnetic equivalency of both dicarbollide cages in a solution. This results in the signal averaging in the NMR time scale due to time-averaged rotational disorder. The ^11^B NMR spectrum of the symmetric *vicinal*-isomer consists of two shifted symmetric patterns of dicarbollide ligands in the spectrum; each part shows six boron signals of relative intensities 1:1:2:2:2:1 [46]. Each isomer typically shows one CH signal with an intensity of two in the corresponding ^1^H and ^13^C spectra, usually differing in chemical shifts. The ratios of isomers can be reliably analyzed using ion-pair reverse-phase (IP-RP) chromatography on RP columns modified with octyl or phenyl groups, which often allows for the baseline separation of the peaks of respective stereoisomers [83]. Compared to normal-phase chromatography, the separations on the RP column often provide better results when considering the preparative separations of diastereoisomers [75].

### 3.4. Chirality

Substitutions on the cobalt bis(dicarbollide) ion can readily induce asymmetry in the cage, leading to chirality. While mainly electroneutral compounds with chirality induced by asymmetric bridging units between boron atoms were previously described and resolved into enantiomers [83,84,85,86], carbon substitutions offer a broader range of possibilities for preparing chiral species.

Monosubstituted compounds with the general formulation [(1-X-1,2-C_2_B_9_H_10_)(1′,2′-C_2_B_9_H_11_)-3,3′-Co(III)]^−^ and [(1-Y^+^-1,2-C_2_B_9_H_10_)(1′,2′-C_2_B_9_H_11_)-3,3′-Co(III)]^0^ are inherently chiral species displaying planar chirality. Trisubstituted derivatives can also exhibit the same type of asymmetry. In the case of disubstitutions with identical groups, of the substitutions with short or rigid bridges, and out of the three possible stereoisomers (Scheme 1, A: *rac*-, B: *meso*-, and C: *vicinal*-), only the *rac*-stereoisomer is inherently asymmetric and represents a prochiral species with axial chirality. Chiral molecules can also be obtained when two different substituents are present on carbon atoms in the *meso*-and *vicinal*-forms or when the second group is located on a boron atom.

Enantioseparations of these chiral ionic cobalt bis(dicarbollide) derivatives using chiral HPLC have been challenging, in particular, due to the unspecific interactions of the anions with silica-gel-based Chiral Stationary Phases (CSPs). Horáček and Kučera recently achieved the successful resolution of this issue by demonstrating that the addition of strong electrolytes to the mobile phase can effectively mitigate these effects [87]. The separation of approximately 16 enantiomeric pairs of chiral ionic, carbon-substituted, cobalt bis(dicarbollide) derivatives on different modified cellulose and cyclodextrin stationary phases was recently described using HPLC and SFC chromatographic techniques [82,88,89,90]. The majority of resolved compounds belong to the type with simple functional groups with preserved rotation of the ligand planes around the cobalt atom. Surprisingly, these compounds could be resolved with similar or even better selectivity and resolution compared to anions rigidified with bridges between B(8,8′) boron positions, indicating that the conformational mobility of the cage has little effect on the separation of enantiomers.

### 3.5. Derivatives and Structural Blocks Prepared through Metalation Reactions

The carbon-substituted compounds in question are categorized and discussed in the following sections based on the chemical origin of the terminal groups attached to the cage. This classification encompasses scenarios where these groups are either separated by a spacer group or not. Particularly in the context of medicinal applications, mostly compounds containing a linker have been studied due to the search for optimal interactions with biological targets.

Notably, compounds with functional groups directly attached to the cage carbon atoms, although they have received less attention, are of significant importance due to their distinct chemical and physicochemical properties. Indeed, even the terminal groups bound to the cage via a methylene spacer show chemically different properties compared to those with ethylene and longer pendant groups [32,91]. In general, the synthesis and isolation of compounds containing groups directly attached to carbon atoms pose various challenges due to the proximal steric and electronic effects of the bulky cobalt bis(dicarbollide) ion. Currently, the first members of the series are the subject of extensive investigation in our laboratories.

#### 3.5.1. Alkyl Derivatives

**Figure 6:**
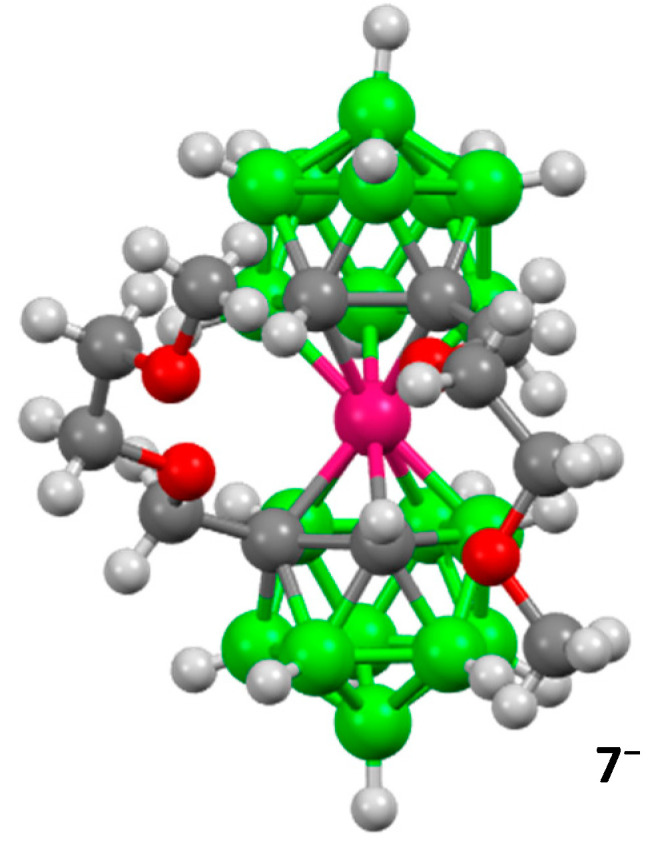
Schematic presentation of the molecular structure of the *rac*-isomer of di-(MEM)^−^ derivative presented in ref. [46]. Cesium atoms are omitted for clarity. Boron atoms are drawn in green, carbon in gray, cobalt in rose, and oxygen in red color.

The first description of deprotonation and nucleophilic substitution reactions in carbon atoms was presented by Miller-Chamberlin et al., who utilized butyllithium lithiation followed by reactions with methylene iodide, hexyl bromide, or the glycolic reagent MEMCl. These reactions resulted in the alkylation of one to four cage carbon atoms [46]. The reactions were conducted in tetrahydrofuran (THF) at ambient temperature or at −30 °C for NMR experiments. Despite detailed NMR studies supporting the synthetic results of the cage deprotonation with BuLi and a thorough characterization of the products, later attempts by different research groups to reproduce these syntheses under the described conditions failed. The NMR results indicated the formation of purple or blue diamagnetic species resulting from the metalation of the C-H sites. Consequently, the reduction of the cobalt atom to Co(II) with BuLi was ruled out. The deprotonated sites kinetically determined the stereochemistry of the substitution reactions. The lithiated form was observed to undergo deuterium exchange with D_2_O. In the case of disubstituted compounds, unseparated mixtures of *rac*- and *meso*-isomers were formed in a ratio of 2.5:1 for methyl and hexyl species, while the pure *rac*-diastereoisomer of the di-(MEM) derivative [1,1′-(CH_3_OCH_2_CH_2_OCH_2_)_2_-(1,2-C_2_B_9_H_10_)_2_-3,3′-Co(III)]^−^ emerged as the sole product (**7^−^**, Figure 6). Larger ratios of BuLi exceeding two equivalents were reported to cause the degradation of the cage, leading to the release of metallic cobalt. The lithiation of boron-substituted mono- and dibromo/iodo-cobalt bis(dicarbollide) (followed by a reaction with MeI) did not yield any methylated derivatives. This can be explained by the interactions of large-sized and electron-rich Cl and Br atoms with the acidic C-H hydrogens in the *transoid* conformation preferred by the halide derivatives [46]. These compounds were synthesized for the purpose of their use in radionuclide extraction.

#### 3.5.2. Hydroxyalkyl Derivatives and Their Respective Esters

**Scheme 2:**
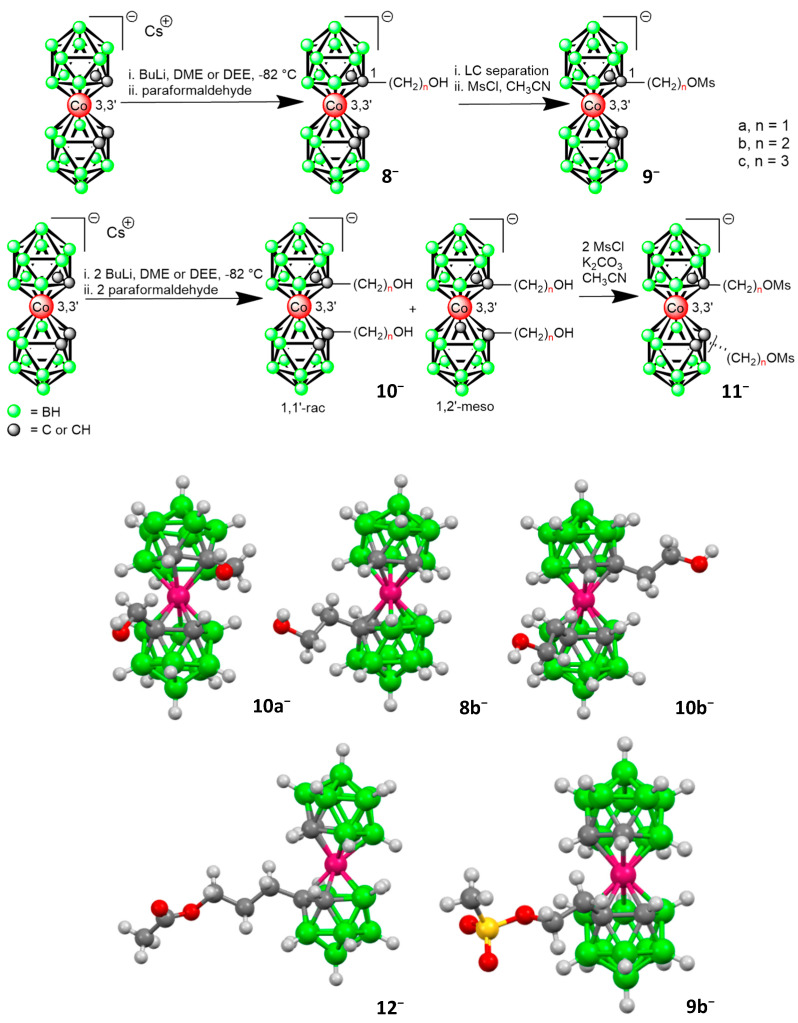
Synthesis of mono- and disubstituted hydroxylalkyl and the respective mesyl esters of cobalt bis(dicarbollide) from the reaction of hydroxyalkyl derivatives with methylsulfonyl chloride; i. Cs_2_CO_3_ or K_2_CO_3_, CH_3_CN or CH_3_CN/toluene; ii. MsCl or TsCl, 45 or 80 °C; ii. MeOH, aq. Me_4_NCl, drying; the schematic presentation of the molecular structures of compounds **10a^−^**, **8b^−^**, **10b^−^**, **12^−^**, and **9b^−^** [75,81]. Boron atoms are drawn in green, carbon in gray, and cobalt in rose, oxygen in red, and sulfur in dark yellow color.

Reactions of lithiated cobalt bis(1,2-dicarbollide)(**1^−^**) in the presence of paraformaldehyde, ethylene oxide, or trimethylene oxide led to the substitution of **1^−^** at the carbon atoms, resulting in the high-yield formation of monosubstituted alkylhydroxy derivatives [(1-HO(CH_2_)_n_-1,2-C_2_B_9_H_10_)(1′,2′-C_2_B_9_H_11_)-3,3′-Co(III)] (n = 1–3) (**8^−^a**, **b**, **c**), along with disubstituted products of general formulation [(HO(CH_2_)_n_-1,2-C_2_B_9_H_10_)2-3,3′-Co(III)]^−^ (n = 1–3) (**10^−^a**, **b**, and **c**) (Scheme 2) [81]. Disubstituted compounds are, in fact, mixtures of 1,1′-*rac*- and 1,2′-*meso*-diastereoisomers. In the case of the longest pendant group in **10c^−^**, the 1,2-*vicinal*-isomer also formed. Initially, only the *rac*-isomer could be isolated in pure form through multiple fractional crystallizations from dichloromethane layered by hexane, in the case of shorter chain compounds **10a^−^** and **10b^−^**. Later, the *meso*-isomer of **10b^−^** and **10c^−^** was also isolated through a combination of crystallization and chromatography [32]. All these alkylhydroxy derivatives have been further utilized as structural blocks in the synthesis of various other derivatives in previous (see Section 5) and ongoing drug design.

These alcohols readily undergo esterification reactions with chlorides and anhydrides of carboxylic acids (see the acetyl ester in Scheme 2). In addition, esters of toluensulfonic or methylsulfonic acid, of the general formula [1-X-O-(CH_2_)_n_-(1,2-C_2_B_9_H_10_)(1′,2′-C_2_B_9_H_11_)-3,3′-Co(III))]Me_4_N (where n = 1–3 and X = -SO_2_CH_3_ or -SO_2_(-C_6_H_4_-4-CH_3_) and diesters [1,1′-(X-O-(CH_2_)_n_)_2_-(1,2-C_2_B_9_H_10_)_2_-3,3′-Co(III)]Me_4_N (n = 1 and 2), are easily available in good to excellent yields using reactions of trimethylammonium salts of alkylhydroxy derivatives with methylsulfonyl or *p*-toluenesulfonyl chloride. Lower conversions were observed only for starting compounds with short methylene linkers [75]. The presence of Me_3_NH^+^ in the starting salt seems essential for attaining good yields of all compounds, due to the catalytic effect of the tertiary amines, a well-known concept in organic chemistry [92]. The Ms and Ts esters were further employed as structural blocks in the synthesis of the respective amines, azides, and sulfamides.

**Figure 7:**
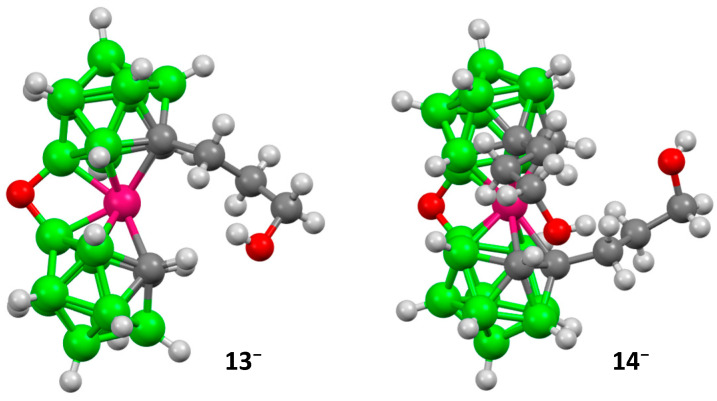
Schematic presentation of the ms of the anions heterosubstituted with one (**13^−^**) or two (**14^−^**) terminal hydroxyalkyl groups, and the oxygen bridge presented in ref. [82]. Cations are omitted for clarity. Boron atoms are drawn in green, carbon in gray, cobalt in rose, and oxygen in red color.

The low-temperature lithiation and reaction with trimethylene oxide were recently studied using the cobalt bis(dicarbollide) cage, which was rigidified by a polar oxygen atom positioned between boron positions (B8,8′), lying in the plane intersecting the bonds between the carbon atoms in dicarbollide ligands located across the central atom [82]. Therefore, the starting anion had a restricted rotation of the ligand planes that corresponded to the symmetric C_2h_ Point Group. The reaction procedure led to the high yield formation of mixtures of hydroxypropyl [*µ*-8,8′-O-(1-HOC_3_H_6_-(1,2-C_2_B_9_H_9_)(1′,2′-C_2_B_9_H_10_)-3,3′-Co(III))]^−^ (**13^−^**) and dihydroxypropyl [*µ*-8,8′-O-(1,1′-(HOC_3_H_6_)_2_-(1,2-C_2_B_9_H_9_)_2_-3,3′-Co(III))]^−^ (**14^−^**) derivatives, with one product prevailing depending on the initial ratios of BuLi used in the reaction. The compounds were characterized using a combination of spectroscopic methods, X-ray crystallography, and chemical analysis. Dissimilarly to the parent cage, only the *racemic*-isomer of dihydroxypropyl derivative formed when the conformation mobility of the cobalt bis(dicarbollide) cage was restricted. As also follows from XRD analysis, the molecular structures of **13^−^** and **14^−^** are completely asymmetric due to substituent sites located on carbon atoms C1 and C(1,1′), respectively. Both types of this heterosubstitution are inherently chiral, and the enantiomers were resolved using analytical HPLC techniques on chiral stationary phases. The molecular structures of these compounds are depicted in Figure 7.

The Me_3_NH^+^ salts of the hydroxypropyl derivatives were easily converted to their corresponding methylsulfonyl (Ms) esters of formulae [*µ*-8,8′-O-[(1-(CH_3_SO_2_O-C_3_H_6_-1,2-C_2_B_9_H_9_)(1,2-C_2_B_9_H_10_)-3,3′-Co(III)]^−^ and [*µ*-8,8′-O-(1,1′-(CH_3_SO_2_O-C_3_H_6_)_2_-(1,2-C_2_B_9_H_9_)_2_-3,3′-Co(III))]^−^.

Recently, the feasibility of the reaction of lithiated cobalt bis(1,2-dicarbollide) with ethylene oxide was confirmed by Śmiałkowski et al. for the production of compounds containing substituents on both boron and carbon sites (2023) [93]. The starting compounds were B(8,8′) substituted derivatives that contained either two -OEtOSi(CH_3_)_2_C(CH_3_)_3_ groups or a phosphorothioate bridge that were substituted with a trityl-protected hydroxy-ethyl group at the sulfur atom (see Section 4). In both cases, the lithiation followed by reactions with ethylene oxide provided the expected type of substitution, with ethoxy groups on carbon atoms. Although low yields of the respective products were obtained, the results represent a rare example of simultaneous substitutions with terminal hydroxy groups bonded to the cage via an alkyl chain on two boron positions, along with the presence of another group on one or two carbons. The products substituted with four groups on two C- and two B-sites are reported to be a mixture of stereoisomers and enantiomers, and these mixtures could not be separated.

#### 3.5.3. Carboxylic Acids and Esters

**Figure 8:**
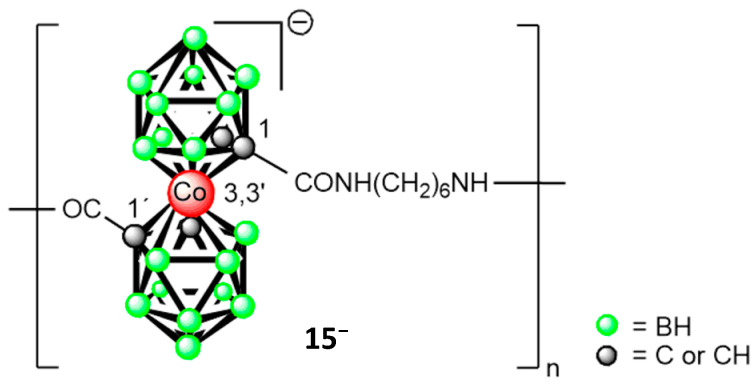
Condensation polymerization product of cobalt bis(dicarbollide) dicarboxylic acid [1-(CONH(CH_2_)_6_NH)-1′-(CO)_2_-(1,2-C_2_B_9_H_10_)_2_-3,3′-Co(III)]_n_^−^ (**15^−^**) [94,98].

The first report on the synthesis of C-dicarboxylic acid appeared in the literature as early as in 1997 [94]. However, the paper primarily focused on polymer synthesis and lacked sufficient details on the synthesis and characterization of the precursor acids. The polyamide polymer backbone incorporating cobalt bis(dicarbollide) anion (**15^−^**) was tested as a cation exchanger for the separation of ^137^Cs^+^ and ^90^Sr(II) from radioactive waste (Figure 8) [95]. This was paralleled by other studies of this team on radionuclide partitioning, which were conducted using extraction agents based on carbon alkylated [96] or boron halogenated [97] cobalt bis(dicarbollide) ions.

**Scheme 3:**
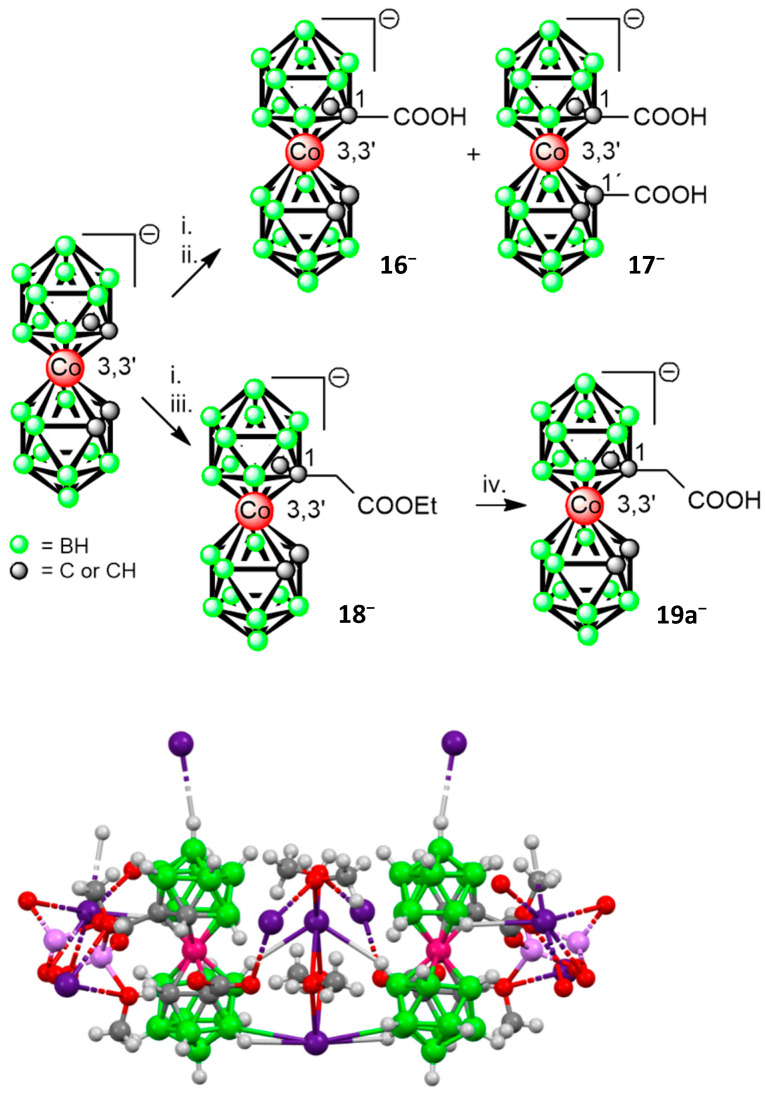
Synthesis of the carboxylic acids **16^−^**, **17a**^−^, and **19a^−^** from **1^−^** by direct lithiation reactions. Reaction conditions: i. DME, −82 °C, n-BuLi; ii. CO_2_(s); iii. BrCH_2_COOEt at −82 °C; iv. aq. NaOH in EtOH, reflux. BH and CH hydrogen atoms are omitted for clarity. Schematic drawing of the molecular structure of the double Cs^+^/Li^+^ salt of dicarboxylic acid **17a^−^** based on unpublished crystallographic data [68]. Boron atoms are drawn in green, carbon in gray, cobalt in rose, oxygen in red, and Li^+^ and Cs^+^ cations in magenta and dark violet color, respectively. The compound was isolated by the crystallization of the reaction mixture from aqueous MeOH.

**Scheme 4:**
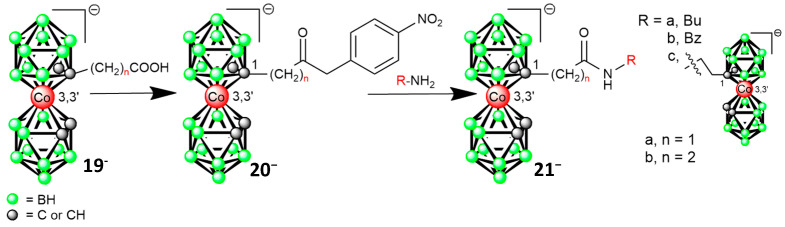
Active esters of cobalt bis(dicarbollide)(**1^−^**) carboxylic acids allow for incorporation of this ion into functional molecules by amidic bonds [99].

Later, a detailed study was published on the reactions of lithiated cobalt bis(1,2-dicarbollide)(**1^−^**) with carbon dioxide in DME that led to the substitution of **1^−^** on the C-atoms by carboxy function(s) [99]. This reaction pathway resulted, according to conditions, in good yields of the monosubstituted and disubstituted carboxylic acids of formulations [(1-HOOC-1,2-C_2_B_9_H_10_)(1′,2′-C_2_B_9_H_11_)-3,3′-Co(III)]^−^ (**16^−^**) and [(HOOC)_2_-(1,2-C_2_B_9_H_10_)_2_-3,3′-Co(III)]^−^ (**17^−^a**, **b**), respectively. The 1,1′-*rac*-isomer (**17a^−^**) forms in this reaction as the main product, along with minor quantities of 1,2′-*meso*-isomer (**17b^−^**, up to 10% by HPLC). Only the 1,1′-*rac*-isomer could be isolated in its pure form. The stereochemistry of these species was supported by the geometry optimizations and calculations of ^11^B NMR shifts at the GIAO-DFT level. Scheme 3 presents a more recent XRD structure of the main 1,1′-*rac*-diastereoisomer that was measured and refined in the meantime [68]. This fully confirms its asymmetric structure. In addition, compounds where the carboxylic function is separated from the cage with different pendant groups were reported in this article. The first has a formula [(1-HOOC-CH_2_-1,2-C_2_B_9_H_10_)(1′,2′-C_2_B_9_H_11_)-3,3′-Co(III)]^−^ (**19a^−^**) and resulted from a lithiation followed by a reaction with BrCH_2_COOEt and the hydrolysis of the respective ethyl ester (**18^−^**). The second compound, which contains an ethylene pendant group, [(1-HOOC-(CH_2_)_2_-1,2-C_2_B_9_H_10_)(1′,2′-C_2_B_9_H_11_)-3,3′-Co(III)]^−^ (**19b^−^**) between the cage and carboxylic group, was prepared by the oxidation of a hydroxypropyl derivative of the ion **1^−^**. *Para*-nitrophenyl esters were also prepared (**20^−^**) as building blocks with the aim of the easy formation of amidic bonds between the boron cage and organic primary amino functions (Scheme 4). Their use was exemplified with model organic amines; butylamide and benzylamide [(1-RNHOC-(CH_2_)_n_-1,2-C_2_B_9_H_10_)(1′,2′-C_2_B_9_H_11_)-3,3′-Co(III)]^−^ (**21^−^a**, **b**) (n = 1, 2; R = Bu, R = Bn) were isolated and characterized. In addition, the feasibility of the synthesis of compounds containing two cobalt bis(dicarbollide) cages, inter-connected via an amidic bond, was also demonstrated.

### 3.6. Nitrogen Containing Compounds

#### 3.6.1. Nitriles

**Scheme 5:**
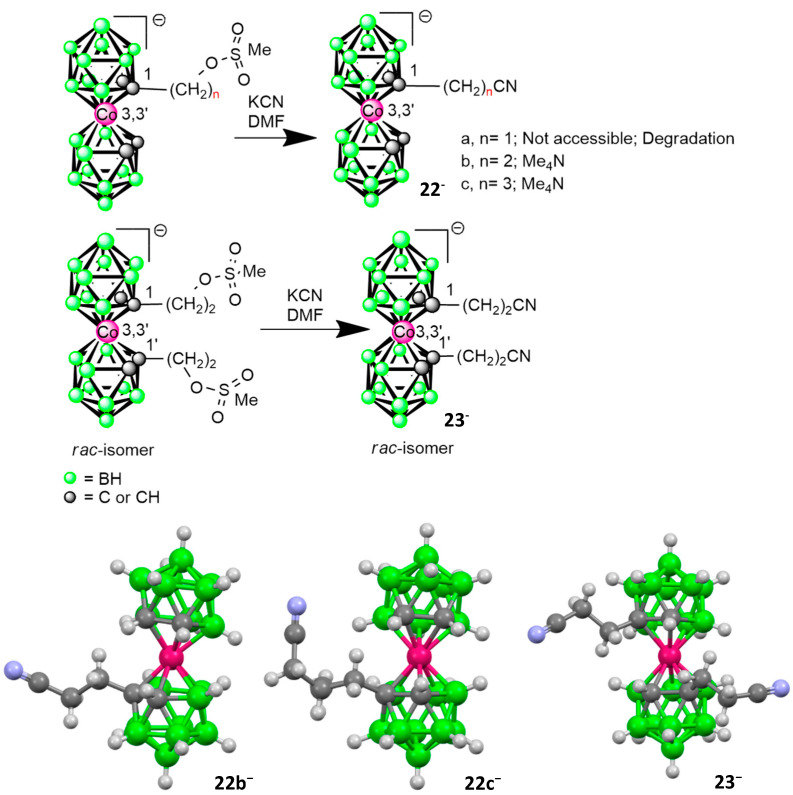
Synthesis of alkylnitrile derivatives of the cobalt bis(dicarbollide) ion starting from mesyl esters, which were prepared according to ref. [91]. Schematic figures of molecular structures of three nitrile derivatives, determined using XRD and reported in ref. [80], are depicted at the bottom. Cations and solvent molecules are omitted for clarity. Boron atoms are drawn in green, carbon in gray, cobalt in rose, and nitrogen in blue color.

Three compounds with terminal nitrile groups have been reported in the literature [80]. These were synthesized through reactions of Ms esters containing longer linkers with KCN in a polar solvent, such as DMF, resulting in the respective alkylnitriles in good to excellent yields (Scheme 5). Compounds with ethylene and propylene connectors between the nitrile group and the cage, with a general formula of [(1-NC(CH_2_)_n_-1,2-C_2_B_9_H_10_)(1′,2′-C_2_B_9_H_11_)-3,3′-Co(III)]^−^ (n = 2 and 3, Me_4_N**22b** and Me_4_N**22c**), were isolated as Me_4_N^+^ salts after chromatography. Using this procedure, a C,C-disubstituted member of the series of formula [1,1′-(NC(CH_2_)_2_-1,2-C_2_B_9_H_10_)_2_-3,3′-Co(III)]^−^ (Me_4_N**23**) was also prepared that comprised two ethylnitrile groups in a *rac*-arrangement. The starting Ms ester was prepared from the pure *rac*-diastereoisomer of the corresponding alcohol, isolated through crystallization [25,81]. On the other hand, the synthesis of the first members of the series containing a short methylene connection failed, apparently due to the steric effects of the cage and ionic repulsion. Interestingly, procedures involving bromoacetonitrile or cyanogen bromide resulted in substitutions with bromine instead (see Section 3.10 on halogen derivatives). These compounds serve as widely applicable building blocks, and their reactivity was tested in dipolar cycloaddition reactions, resulting in the formation of the tetrazole motif.

#### 3.6.2. Azides

**Scheme 6:**
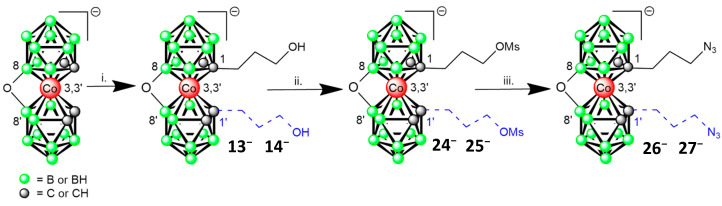
Synthesis of C-alkylazides of the oxygen bridged cobalt bis(dicarbollide) ion. Reaction conditions: i. BuLi, trimethylene oxide, DME, −78 °C, then Me_3_NH^+^; ii. MsCl, K_2_CO_3_, CH_3_CN, 45 °C, then Me_4_N^+^; iii. NaN_3_, 45 °C [82].

Monosubstituted propylazide and *rac*-isomer of dialkylazide [*µ*-8,8′-O- [(1-(N_3_C_3_H_6_-1,2-C_2_B_9_H_9_)(1,2-C_2_B_9_H_10_)-3,3′-Co(III)]^−^ and dipropylhydroxy [*µ*-8,8′-O-(1,1′-(N_3_C_3_H_6_-1,2-C_2_B_9_H_9_)_2_-3,3′-Co(III)]^−^ (**26^−^**, **27^−^**) were successfully prepared by the reaction of the corresponding Ms esters **24^−^** and **25^−^** with NaN_3_ in dry dimethyl formamide (DMF) (Scheme 6). Only the azides derived from the bridge compound with conformationally restricted geometry have been reported thus far. These compounds were then studied as building blocks suitable for Huisgen–Sharpless dipolar [2 + 3] cycloadditions with organic alkynes [82]

#### 3.6.3. Amines

**Scheme 7:**
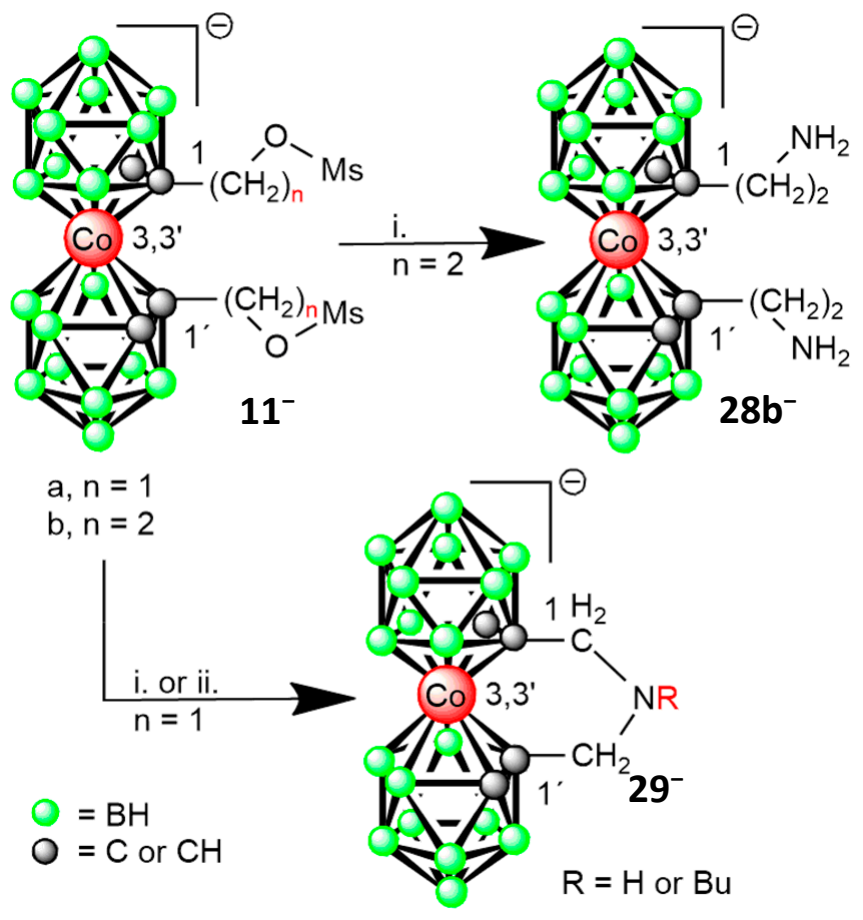
Diethylamine substituted cobalt bis(dicarbollide) (**34^−^**) and bridge derivatives (**35^−^a, b**) from the reactions of monosubstituted methylsulfonyl and p-toluenesulfonyl esters of cobalt bis(dicarbollide) with excess of ammonia, primary and secondary amines; i. NH_3_ in excess, heating; ii. nBuNH_2_, 60 °C; R = H or n-butyl [99].

C-substituted alkylamine derivatives of the cobalt bis(dicarbollide) ion are readily available when starting from primary hydroxyalkyl derivatives with varying lengths of the aliphatic spacer between the C(1) or C(1) and C(1′) cage positions. The reaction pathways involve two steps: the conversion of hydroxyalkyl derivatives to methylsulfonyl or p-toluenesulfonyl esters and their subsequent reactions with ammonia or primary and secondary amines in toluene. These reactions resulted in compounds with primary, secondary, and tertiary alkylamine groups attached to the carbon C(1) cage positions. These synthetic routes also enable the easy disubstitution of the cobalt bis(dicarbollide) with two amine functions (**28**^−^). Less satisfactory results were obtained only for groups attached to the cage via a methylene linker. In particular, the dimesyl ester (**10a^−^**) was shown to react with ammonia and primary amines in a different pathway, producing bridge structures consisting of three atoms of the formula [(*μ*-(-CH_2_NRCH_2_)-(1,2-C_2_B_9_H_10_)_2_-3,3′-Co(III))]^−^ (R = H or Bu) (**29^−^a**, **b**). In our opinion, this synthetic route is the most reliable and convenient method for the synthesis of C-substituted amines, although three reaction steps are necessary. An example of disubstituted compounds is shown in Scheme 7 [99].

**Scheme 8:**
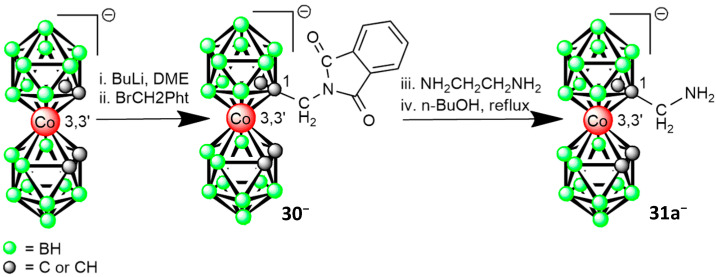
Synthesis of aminomethyl (**32^−^**) cobalt bis(dicarbollide) by low temperature reaction of bromomethyl phthalimide with lithiated Cs**1** and subsequent cleavage of the protective group (**31^−^**). Alternatively, NaBH_4_ in *i*-PrOH-water can be used, followed by hydrolysis by 3 M HCl in glacial acetic acid [79].

In principle, alkylamines are accessible from reactions of halogenoalkyl amines with protected amino functions (using phthalimido, *t*-Bu-carbonyl, or other protective groups). However, this reaction pathway is compromised by a nucleophilic substitution that may simultaneously proceed on the carbonyl group (if present in the particular protective group). Thus, only one successful reaction is described in the literature, which consists of the treatment of the lithiated anion **1^−^** with bromomethyl phthalimide. This produces, after deprotection, methylene amine in a low yield of up to 18% (Scheme 8) [79].

### 3.7. Nitrogen-Containing Heterocyclic Compounds

#### 3.7.1. Triazines

**Scheme 9:**
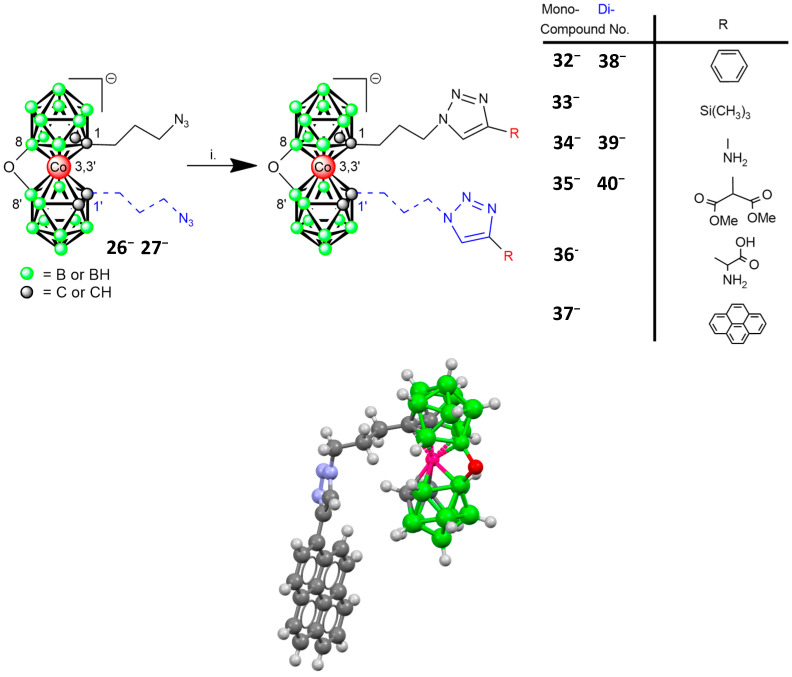
Conjugation of azides with conformationally restricted geometry with organic functional groups via copper-catalyzed (2 + 3) cycloaddition reactions. Reaction conditions: CuI, DIPEA, alkyne, 37 °C, and the schematic drawing of the molecular structure of product **37^−^** containing pyrene substituted triazine ring (in bottom), which is based on unpublished XRD data [68]. Boron atoms are drawn in green, carbon in gray, cobalt in rose, oxygen in red and nitrogen in blue color.

El Anwar et al. reported a series of compounds heterosubstituted with 1*H*-1,2,3-triazole ring and an oxygen bridge between boron sites, which was synthesized starting from azidopropyl derivatives with conformationally restricted geometry by the presence of an oxygen bridge [82]. The main aim of this study has been the testing and development of reaction conditions suitable for the Huisgen–Sharpless [2 + 3] cycloaddition reactions of azides with alkynes. This would allow for an easy and reliable merging of cobalt bis(dicarbollide) ion with complex organic functional molecules and bioconjugations. The reactions of the boronated alkylazides with model alkynes such as phenylacetylene, ethynylsilane, trimethylsilane, propargyl amine, dimethylpropargyl malonate, propargyl glycine, and ethynylpyrene were tested. The optimized procedure comprised the use of CuI (5 mol%) together with *N,N*-diisopropylethylamine (DIPEA) in anhydrous ethanol at 37 °C over a period from 20 h to 5 days. In general, using these conditions, reactions of **36^−^** with most of the alkynes provided the expected products of the general formula [*µ*-8,8′-O-(1-(4-*R*-1,2,3-triazolyl)-(1,2-C_2_B_9_H_9_)(1′,2′-C_2_B_9_H_10_)-3,3′-Co(III))]^−^ (**33^−^** to **36^−^**) in good yields. Also, the feasibility of reliable disubstitutions was studied starting from diazidopropyl derivative **27^−^**. This was supported by the successful synthesis of compounds [*µ*-8,8′-O-[(1-(4*-R*-1,2,3-triazolyl-1,2-C_2_B_9_H_9_)_2_-3,3′-Co(III)]^−^ **38^−^** to **40^−^** (Scheme 9). Difficulties encountered due to the tedious isolations and the removal of side products could be seen in the case of **35^−^** and **40^−^**. This was reflected in the low yields of both latter compounds (12%). Drawing based on an unpublished XRD structure of tetrazole derivative **37^−^** is shown in Scheme 9, bottom. All these compounds are inherently chiral. This has been verified by the enantioseparation of a large part of these anionic species using HPLC techniques on chiral stationary phases [82].

#### 3.7.2. Tetrazoles

**Scheme 10:**
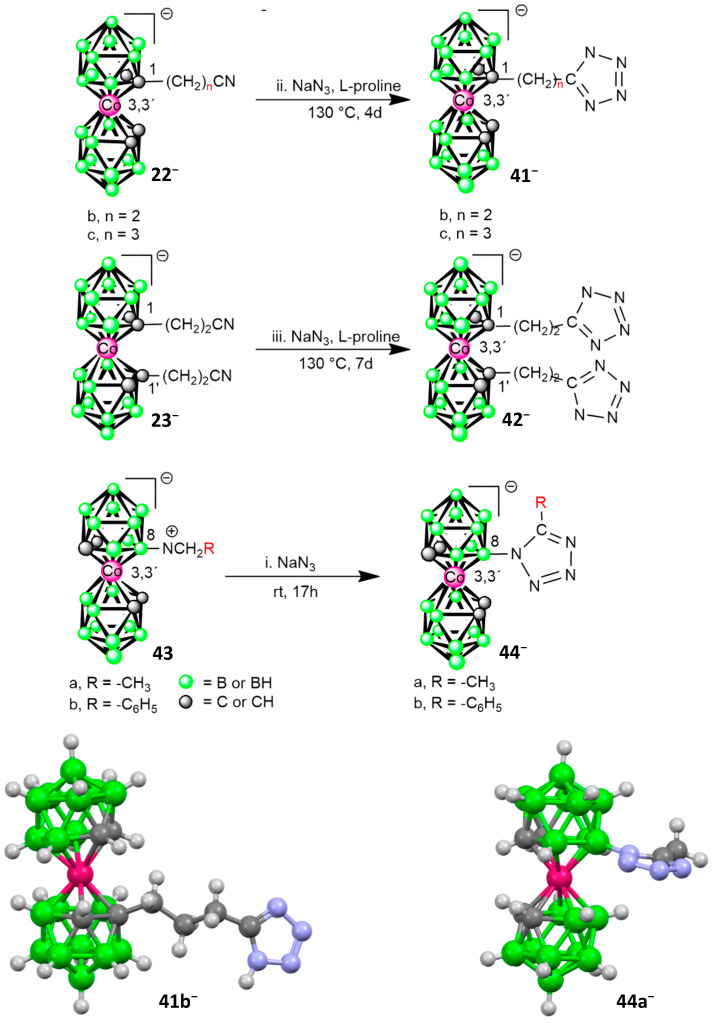
Formation of tetrazoles from nitrilium and isonitrilium derivatives of cobalt bis(dicarbollide) and sodium azide. The drawings of the molecular structures in the bottom part are based on structural data from ref. [80]. Boron atoms are drawn in green, carbon in gray, cobalt in rose, and nitrogen in blue color.

El Anwar et al. (2020) used an advanced organocatalysis approach in the cycloaddition reactions of nitriles with azide ion [80]. Thus, L-proline catalyzed reactions of nitriles **22^−^b**, **c** with NaN_3_ in DMF provided substitution of the cobalt bis(dicarbollide) ion with a tetrazole ring and compounds of the general formula [(1-(tetrazol-5-yl)-(CH_2_)_n_-1,2-C_2_B_9_H_10_)(1′,2′-C_2_B_9_H_11_)-3,3′-Co(III)]^−^ (**41^−^a**, **b**, Scheme 10). Prolonged heating at 130 °C for 4–11 days was needed, however, the products were obtained in good yields and purity after necessary purifications using chromatography and crystallization. In addition, disubstituted compound comprising two tetrazole rings per cobalt bis(dicarbollide) cage of formula [(1-(tetrazol-5-yl)-C_2_H_4_)-1,2-C_2_B_9_H_10_)_2_-3,3′-Co(III)]^−^ (**42^−^**, Scheme 4) was prepared in good yield, starting from *rac*-form of the di(ethylnitrile) **23^−^**. Alternatively, cycloadditions using a previously known isonitrilium derivative [8*-R*-CN-(1,2-C_2_B_9_H_10_)(1′,2′-C_2_B_9_H_11_)-3,3′-Co(III)]^0^ (**43a**, **b** R = Me, Ph) bonded via a cage boron atom B(8), were studied for comparison. The reactions with NaN_3_ proceeded readily even without a catalyst in CH_3_CN at room temperature, and afforded products of the formulae [(8-(5-Me-tetrazol-1-yl)-1,2-C_2_B_9_H_10_)(1′,2′-C_2_B_9_H_11_)-3,3′-Co(III)]^−^ (**44a^−^**) and [(8-(5-Ph-tetrazol-1-yl)-1,2-C_2_B_9_H_10_)(1′,2′-C_2_B_9_H_11_)-3,3′-Co(III)]^−^ (**44b^−^**) which were obtained in good isolated yields. On the other hand, the corresponding reactions with organic azides could be accomplished in neither case, despite a variety of tested catalysts and conditions. The molecular structures of two compounds corresponding to both types of tetrazole derivatives Me_3_NH**41b** and Me_3_NH**44a** were determined using single-crystal X-ray diffraction analysis and are presented in Scheme 10, bottom. The 5-substituted tetrazole species belongs to key structural motifs that mimic carboxylic functions and provide additional interactions and hydrogen bonding, which has been widely introduced into drug design [100]. The structural modifications of the cobalt bis(dicarbollide) ion with a tetrazole ring may help to further tune its properties as a hydrophobic pharmacophore.

#### 3.7.3. Isoindolones and Related Species

**Scheme 11:**
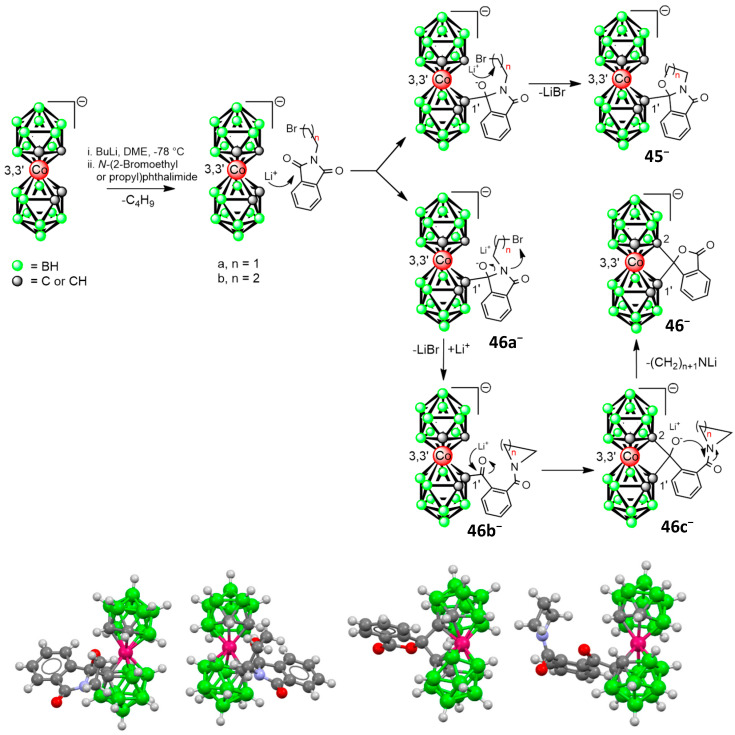
Proposed mechanism for the formation of the main cyclic products **45^−^** and **46^−^** via low temperature reaction of bromoethyl or bromopropyl phthalimide with lithiated Cs**1** and the schematic images of molecular structures of the compounds determined by XRD and presented in ref. [79], cations and solvent atoms are omitted for clarity. Boron atomsare drawn in green, carbon in gray, cobalt in rose, nitrogen in blue, and oxygen in red color.

Grűner et al. reported that the low-temperature lithiation of cobalt bis(dicarbollide) ion and subsequent reactions with *N*-(ω-bromoalkyl)phthalimides, Br-(CH_2_)_n_-N(CO)_2_NC_6_H_4_ (where n = 2 and 3) give compounds substituted at cage carbon atoms by tricyclic isoindolone moieties, with five- (**45a^−^**) or six-member (**45b^−^**) lateral oxazine rings as the predominant products. In addition, two minor products (**46^−^**) were isolated that correspond to unusual cyclic substitution. Anion **46^−^** contains a bicyclic benzofuranone ring attached by a quaternary carbon in a bridging manner, and anion **46^−^** consists of a ketobenzoic acid amide substituent with a lateral azetidine ring [79]. The structures of all the isolated cyclic products were determined using single-crystal X-ray crystallography. As depicted in Scheme 11, the first step in the main reaction mechanism consists of the nucleophilic addition of the lithiated carbon of the metallacarborane to the carbonyl moiety present in the phthalimide group. This is followed by the intramolecular cyclization process of the alkylbromide end of the molecule, which results in the formation of a tricyclic heterocycle of the isoindolone type. Only the later stages of the reaction correspond to an SN_2_ mechanism in which the nucleophilic alcoholate oxygen reacts with the alkylbromide end and forms the lateral ring in the isoindolone scaffold (see Scheme 11). The formation of a stable pentagonal or hexagonal ring apparently contributes significantly to the observed cyclization pathways. The mechanism that rationalizes the formation of the isoindolone rings thus closely resembles that observed previously for other metalated species such as phenylithium [101] or lithiated 1-phenyl-1,2-carborane [102].

### 3.8. Silicon Derivatives

**Scheme 12:**
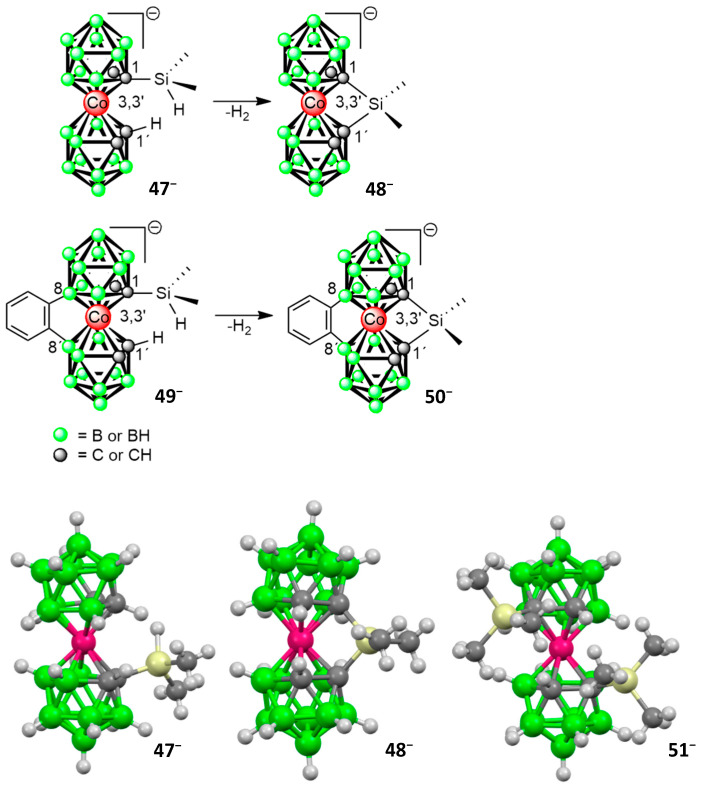
The formation of the bridge due to the elimination of the hydrogen molecule from cage-C-H and hydridic Si-H is shown on top; and the schematic drawings of the molecular structures of the trimethylsilyl, bridged dimethylsilyl, and bis(trimethylsilyl) derivatives determined using XRD [48] are presented on bottom. Boron atoms are drawn in green, carbon in gray, cobalt in rose, and silicon in pale yellow color.

Teixidor’s group reported the synthesis of bridged and non-bridged dimethylsilyl derivatives of cobalt bis(dicarbollide) anion after lithiation at low temperatures [48,103]. Dimethylsilyl-bridged derivative can be obtained using Me_2_SiCl_2_ or even Me_2_SiHCl in high yield due to the loss of H_2_ from the acidic proton of the cage carbon and hydridic Si-H, which results in bridged substitution between carbon sites, compound [1,1′-*µ*-(Me_2_Si)-(1,2-C_2_B_9_H_10_)_2_-3,3′-Co(III)]^−^ (**48^−^**). The non-bridged mono-substituted derivatives can be obtained in lower yields compared to their bridged derivatives (Scheme 12). The non-bridged mono-substituted derivative [(1-Me_3_Si-1,2-C_2_B_9_H_10_)(1′,2′-C_2_B_9_H_11_)-3,3′-Co(III)]^−^ (**47^−^**) and disilylated compound [1,1′-(Me_3_Si)_2_-(1,2-C_2_B_9_H_10_)_2_-3,3′-Co(III)]^−^ (**51^−^**) could be obtained via the reaction of the lithiated cobalt bis(dicarbollide) ion with Me_3_SiCl [48].

In addition, both of the silyl reagents gave the *meso*-substituted doubly bridged dimethyl silyl-cobalt bis(dicarbollide) when the reaction was started from lithiated 8,8′-*µ*-C_6_H_4_-1,2-cobalt bis(dicarbollide) (**49^−^**). The preference of *meso*-form in (**50^−^**; Scheme 12) is explained in terms of the presence of a phenylene bridge, which does not allow for the inclination of the dihedral angle that would enable the attaining of optimal bond lengths in the C(1)-Si-C(1′) bridging group. This represents a unique example when *meso*-isomer forms preferentially. In this case, the singly bound analogue could not be isolated due to H_2_ loss from the structure. While the intermediate from the rotation-restricted phenylene derivative could have never been isolated, it was possible to isolate the mono-silylated intermediate of the parent ion, cobalt bis(dicarbollide), [1,1′-*µ*-SiMeH-(1,2-C_2_B_9_H_10_)_2_-3,3′-Co(III)]^−^, which might be due to the availability of rotation in the structure.

DFT calculations supported the experimental outcome that the *rac*-isomers are more stable than *meso*- which leads to the preferential formation of *rac*-isomer and would also allow for the chiral separation of the enantiomers [48]. It was demonstrated that temperature may play a role in the stereoselectivity of these reactions. Relatively high temperatures, such as −40 °C, favored the formation of *meso*-derivative and, in turn, a higher ratio of *rac*-isomer formed at *−*78 °C. The [1,1′-*µ*-SiMeH-(1,2-C_2_B_9_H_10_)_2_-3,3′-Co(III)]^−^ derivative was used in the regiospecific hydrosilylation reactions of vinyl-terminated dendrimers, producing species decorated with up to eight boron clusters on the periphery [48].

### 3.9. Phosphorylated Derivatives

**Figure 9:**
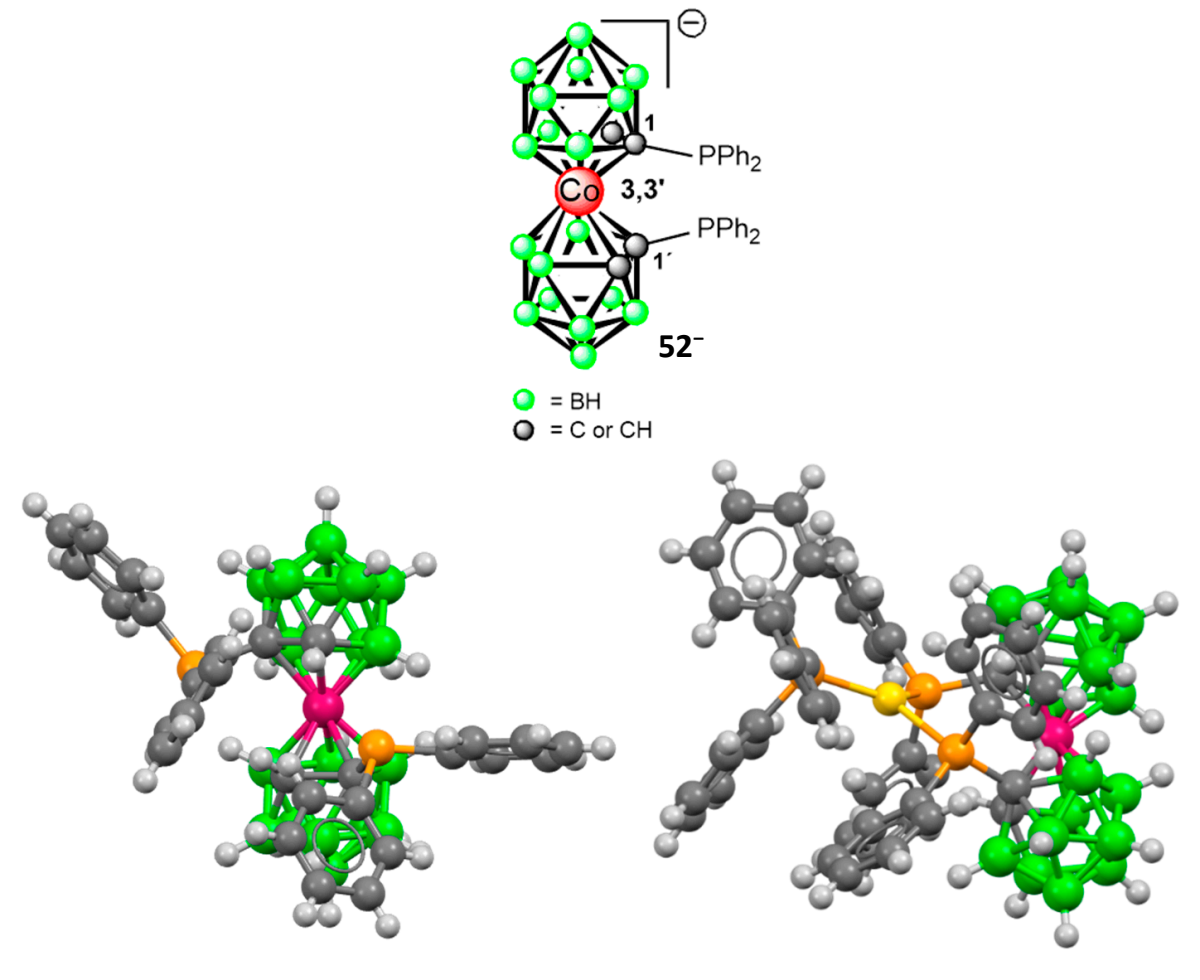
Schematic presentation of the bis(diphenylphosphine) derivative *rac*-(PPh_2_)_2_-cobalt bis(dicarbollide) (**52^−^**) [47]; and molecular structures of *rac*-bis(PPh_3_) ligand **52^−^** and its gold complex PPh_3_Au.**52** drawn from XRD data presented in ref. [47]. Boron atoms are depicted in green, carbon in gray, cobalt in rose, phosphorus in orange, and gold in yellow color.

The synthesis of compounds containing two diphenylphosphine groups was prepared via the direct reaction of lithiated anion **1^−^** with diphenyl chlorophosphine in DME at a low temperature (Figure 9).

The compound [1,1′-(PPh_2_)_2_-(1,2-C_2_B_9_H_10_)_2_-3,3′-Co(III)]^−^ **52^−^**, with two diphenylphosphine groups, has been denoted as a versatile ligand available for metal complexation and has been denoted here as an alternative of organic BINAP ligand or chiral ferrocenyl phosphines with asymmetric substitution [47]. Indeed, this derivative was prepared in *rac*-form and can potentially serve as a versatile chiral auxiliary for metal complexation in asymmetric catalysis. The coordination of rhodium, palladium, silver, and gold with phosphine groups was described, which resulted in the formation of an *ansa*-arrangement of the complexes that contain the triatomic -Ph_2_P-ML-Ph_2_- bridging group. Three XRD structures of these complexes were determined using X-ray crystallography. The XRD structure of the PPh_3_Au.**52** complex is shown in Figure 9, bottom. The two metal coordination sites have been shown to be less separated than in the case of the corresponding phosphinoferrocenes and more mutually separated than in BINAP. Therefore, the plane-intersecting PPh_2_ has a different relative orientation with respect to the rotation axis.

**Scheme 13:**
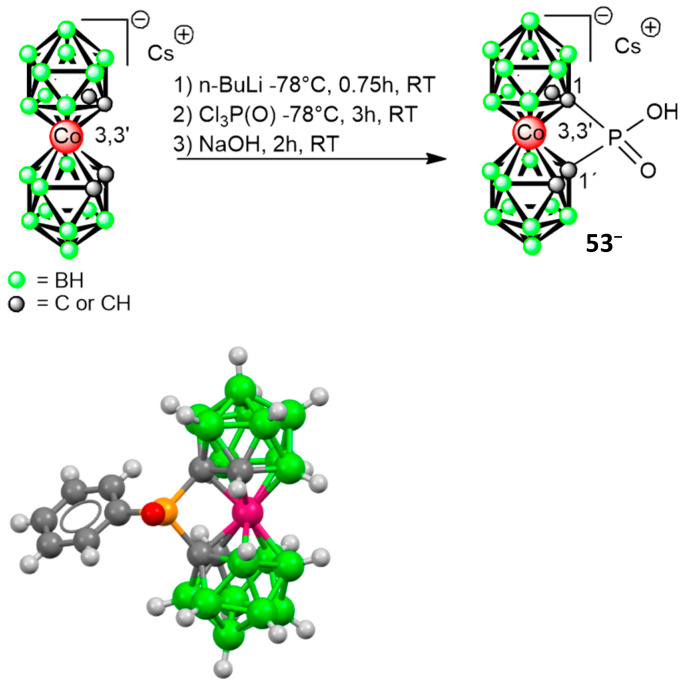
Bridge-substituted phosphine oxide derivative **53^−^** of cobalt bis(dicarbollide) and its molecular structures are drawn from XRD data presented in ref. [48]. Boron atoms are drawn in green, carbon in gray, cobalt in rose, phosphorus in orange, and oxygen in red color.

Later, the reaction of the lithiated anion **1^−^** with phosphoryl chloride was studied that provided, after hydrolysis, substitution at two carbon sites, which was denoted as a bridged derivative of the formula [1,1′-*µ*-HO(O)P-(1,2-C_2_B_9_H_10_)_2_]^−^ (**53^−^**). Thus, the product was characterized using XRD as *rac*-isomer (Scheme 13), bottom.

**Scheme 14:**
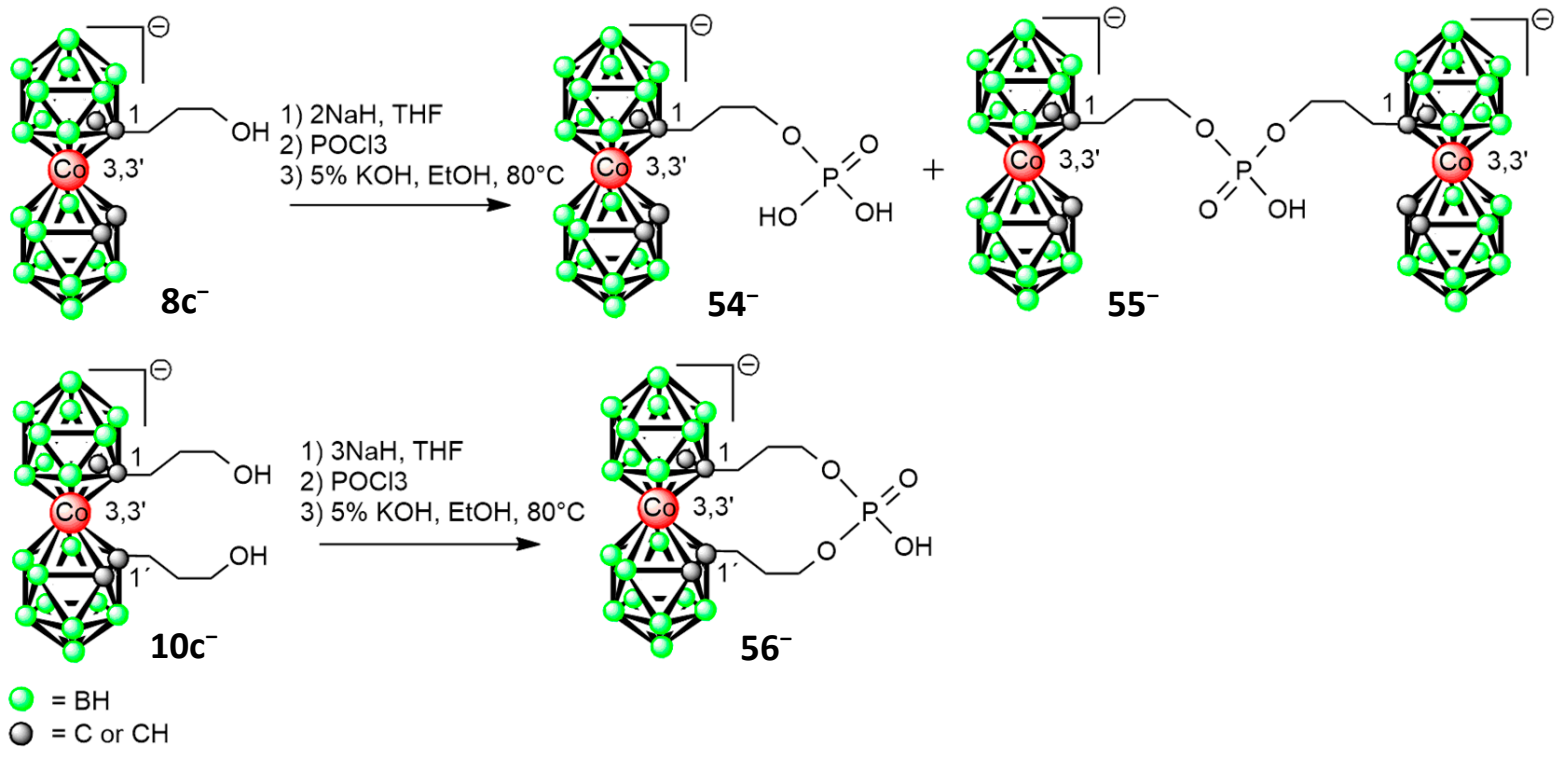
Preparation of phosphoric acid derivatives [81].

Grűner et al. developed another series of phosphorus derivatives containing a phosphoric group that was separated from the cage by a propylene pendant group. A compound substituted with one group was prepared by a reaction of hydroxypropyl derivative Me_3_NH**8c** with NaH and one equivalent of POCl_3_ [81]. This resulted, after hydrolysis, in a phosphorylated compound of the formula [(1-(HO)_2_P(O)OC_3_H_6_)-1,2-C_2_B_9_H_10_)(1′,2′-C_2_B_9_H_11_)-3,3′-Co(III)]^−^, with Me_3_NH**54** as the main product. The corresponding reaction of the hydroxypropyl derivative with half of the equivalent produces a high yield of phosphoric acid diester (Me_3_NH)_2_**55**, in which structure two cages are connected via a propylene linker(s) to the central phosphoric acid moiety. The calcium salt Ca(**56**)_2_ of the bridged ion [(*μ*-(HO)P(O)(OC_3_H_6_)_2_-(1,2-C_2_B_9_H_10_)_2_-3,3′-Co(III)]^−^ was isolated from the reaction of Me_3_NH**10c** with NaH and one equivalent of POCl_3_, followed by hydrolysis and the addition of CaCl_2_. All new compounds were characterized using multinuclear NMR spectroscopy and mass spectrometry (Scheme 14).

### 3.10. Halogen Derivatives

**Scheme 15:**
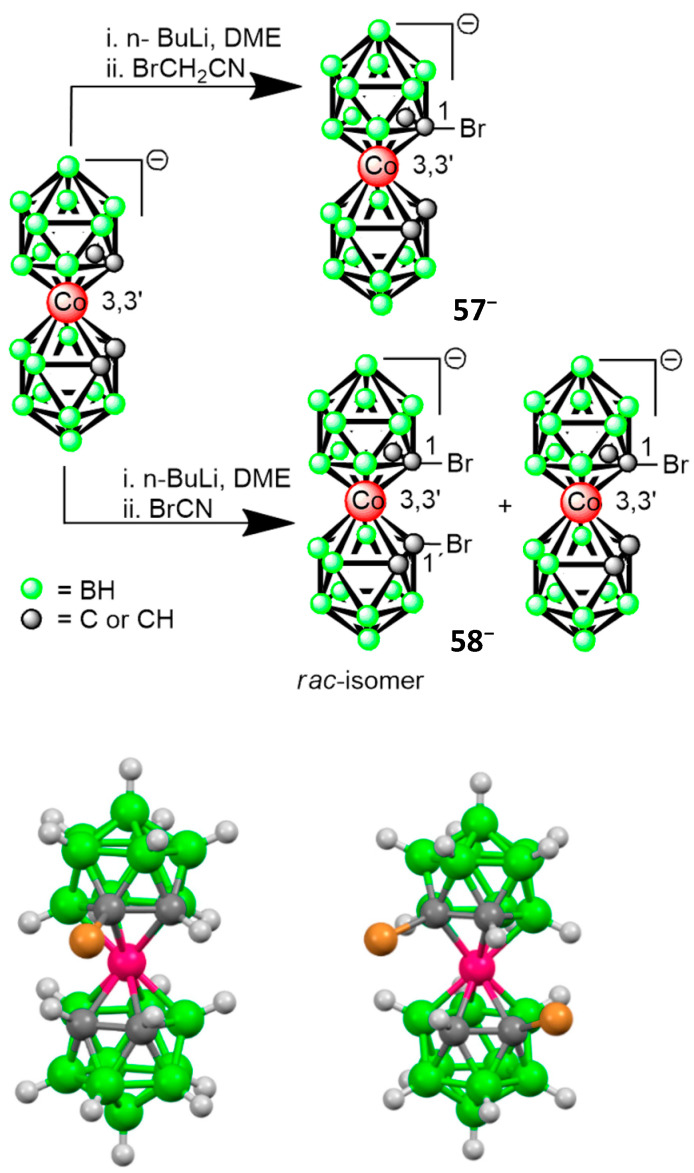
Synthesis of bromo derivatives of the cobalt bis(dicarbollide) ion and schematic drawings of their molecular structures determined using XRD (**57^−^, 58^−^**) and presented in ref. [80]. Boron atoms are drawn in green, carbon in gray, cobalt in rose, and bromine in brown color.

The treatment of lithiated cobalt bis(dicarbollide) with bromoalkylnitriles resulted in mono- and di-bromo derivatives, unexpectedly. Hence, the reaction with 1-bromoacetonitrile gave low yields of monosubstituted product of the formulation [(1-Br-1,2-C_2_B_9_H_10_)(1′,2′-C_2_B_9_H_11_)-3,3′-Co(III)]^−^ (**57^−^**). A similar low-temperature reaction (−78 °C, Scheme 15) of the cobalt bis(dicarbollide) ion lithiated by two equivalents of n-BuLi in DME with BrCN proceeded smoothly with high conversion to C,C-dibromide [Br_2_-(1,2-C_2_B_9_H_10_)_2_-3,3′-Co(III)]^−^ (**58^−^**), along with a smaller quantity of monobrominated compound **57^−^** [80]. Thus, in both instances, the bromine atom acts as a preferred reagent, possibly due to its properties lying on the borderline between soft and hard electrophiles. It was assumed in this study that the lithiated large cobalt bis(dicarbollide) ion might be, due to its bulky structure and low electron density, considered to act as a soft nucleophile that would react preferentially with soft electrophiles rather than with hard ones like CN^+^ or ^+^CH_2_CN. The disubstituted compound was separated using chromatography combined with crystallization and characterized as a mixture of all three possible diastereoisomers, which were present in an approximate ratio of 5:1:1 according to NMR. Pure *rac*-isomer [1,1′-Br_2_-(1,2-C_2_B_9_H_10_)_2_-3,3′-Co(III)]^−^ was then isolated by repeating the chromatography on an RP-C18 column using 55% aqueous MeOH as the mobile phase.

In addition to all the carbon-substituted cobalt bis(dicarbollide) derivatives mentioned in the above sections, derivatives with terminal sulfamide, sulfonamide, and phthalimide groups were prepared (please see details in Section 5.2) focusing on medicinal applications.

The carbon-substituted derivatives currently form a portfolio of structural blocks applicable in diverse areas of chemical research. They form viable alternatives compared to more elaborated chemistry on boron vertices and offer new opportunities in tuning the spatial arrangement of substituents, dipole moments, interactions with medicinal targets, and in the development of chiral platforms. This has already been shown by the applications of carbon-substituted derivatives in drug design. Several types of new compounds addressing different therapeutic targets have already been prepared and studied, often showing improved properties when compared with related boron-substituted analogues (see Section 5) devoted to medicinal applications.

## 4. Recent Progress in Boron Substitution

**Scheme 16:**
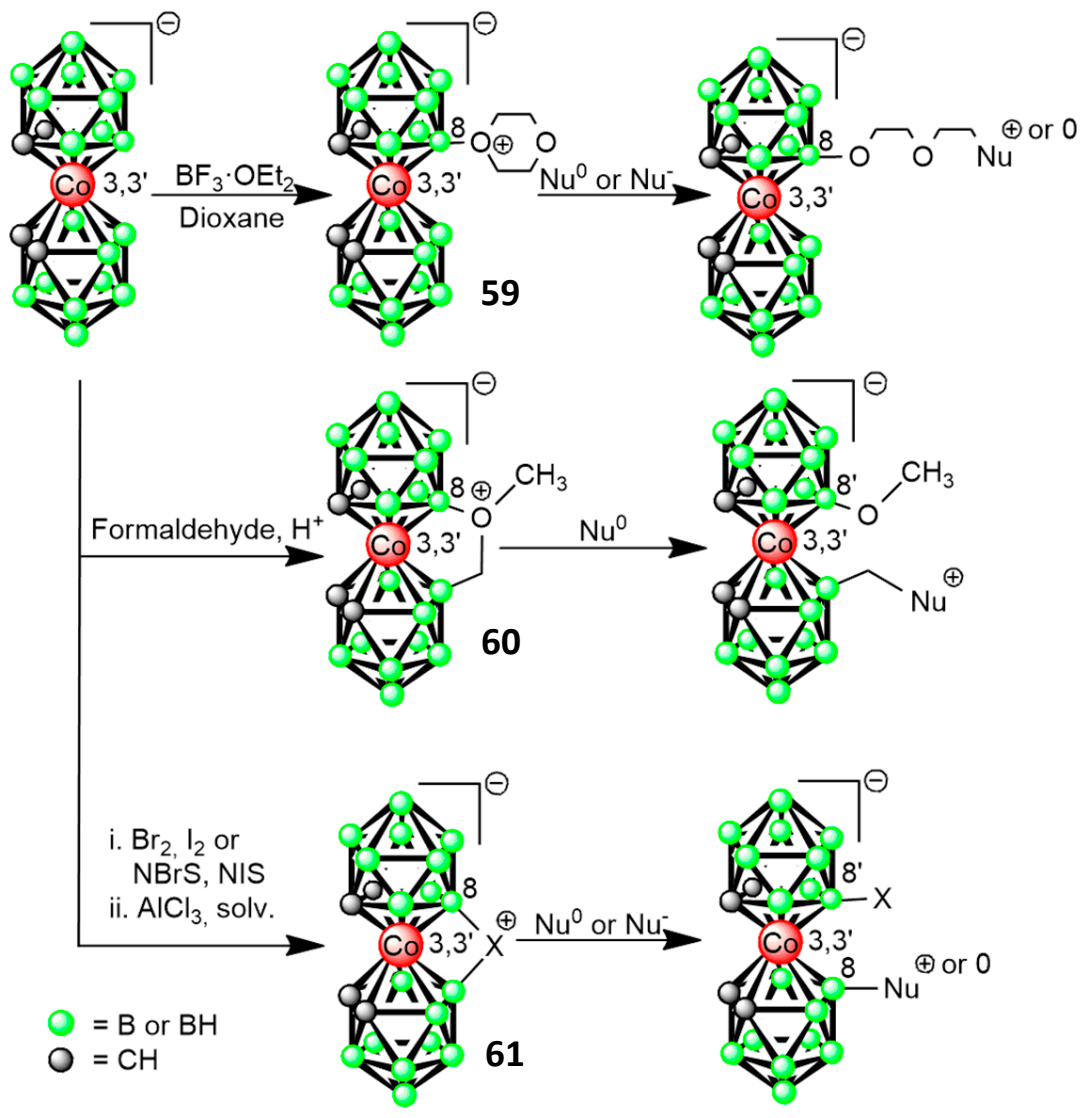
General synthetic routes for easy introduction of variety group(s) and molecule(s) to boron atom(s).

The demand for new derivatives of cobalt bis(dicarbollide) ion continues to drive ongoing synthesis efforts. Over the past 30 years, significant progress has been achieved in synthesizing derivatives through ring-opening reactions of cyclic oxonium derivatives when treated with various nucleophiles. This particular reaction, initially invented by Plešek [104,105], provides a highly efficient method for introducing functional groups onto the B(8) atom of the cage. It is sometimes referred to as “boron-click” due to its effectiveness in producing various types of derivatives with functional groups attached to the boron atoms via a flexible six-atom spacer [106,107,108,109]. These reactions have been the subject of review articles published in 2008, 2012 [110,111], and 2021 [112]. For some terminal groups, it has been observed that the six-atom spacer can undergo cleavage to form a shorter chain consisting of a B-OCH_2_CH_2_OH group [113]. This offers new opportunities for tuning the distance between the functional group and the cage. Another type of oxonium compound of this kind consists of a diatomic (-O^+^(CH_3_)-CH_2_-) bridge in (B8,8′) (**60**) positions of the cage. Cleaving this bridge with nitrogen nucleophiles results in a short methylene linker present in the B(8)-CH_2_-Nu unit [114]. However, the use of this building block has certain limitations due to demethylation, which, in some circumstances, proceeds preferentially. The latter pathway occurs with oxygen nucleophiles and some bulky amines. Due to their high versatility, these methods have been extensively investigated since they represent an easy solution for introducing a wide variety of functional groups and bioorganic molecules to boron atoms (Scheme 16) [112,115]. Another widely applicable method, which was also initiated by Plešek et al., consists of a similar cleavage of a monoatomic bromonium or iodonium bridging moiety between (B8,8′) positions (**61**) [116]. This cleavage results in the formation of a B(8)-Nu bond along with a B(8′)-Hal substitution (**61**) [104,117,118].

From the perspective of tuning the spatial arrangements of substituents on the cage, especially for interactions with medicinal targets and materials science, recent results on compounds substituted at “unusual” boron sites B(10), B(4), and B(9) are particularly important.

Safronov, Hawthorne et al. described disubstituted iododerivative in apical positions B(10,10’) accessible through the degradation of the corresponding 8-I-dicarba-*closo*-dodecaborane followed by the insertion of the cobalt atom [119].

**Figure 10:**
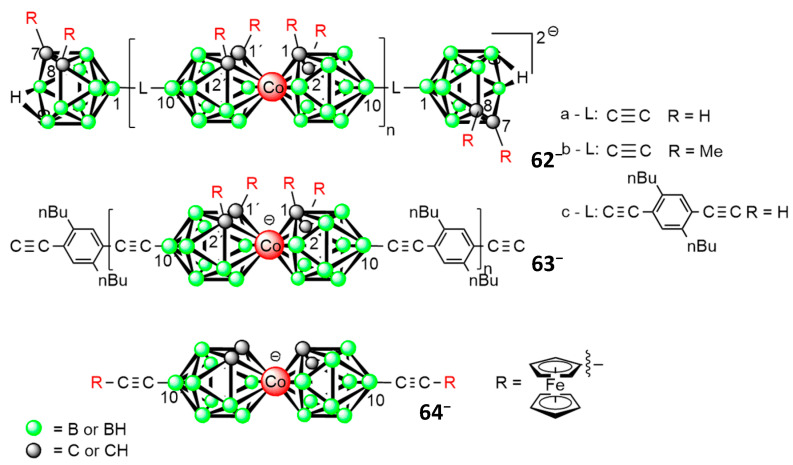
Structural formula(s) of compound(s) {7,8-*R*_2_-C_2_B_9_H_8_-[1,1′,2,2′-(*R*)_4_-10-*L*-(1,2-C_2_B_9_H_8_)_2_-3,3′-Co(III)]_n_}^2−^ **62^−^**, {[1,1′,2,2′-(*R*)_4_-10-((2,5-C_4_H_9_)_2_-Ph)-C_2_)-10′-C_2_-(1,2-C_2_B_9_H_8_)_2_-3,3′-Co(III)]}_n_^−^ (**63^−^**), [10,10′-(*R*-C_2_)_2_-(1,2-C_2_B_9_H_10_)_2_-3,3′-Co(III)]^−^ (**64^−^**).

Pichaandi et al. described two approaches for synthesizing rod-like polymers containing metal bis(dicarbollide) anions connected by organic linkers. In the first synthetic route, *nido*-bis(carborane) precursors with acetylene or 1,4-dibutyl-2,5-diethynylbenzene linkers were used. The synthesis involved the formation of the cobalt bis(dicarbollide) dianion through the reaction of *nido*-bis(carboranes) with n-butyllithium, followed by metal insertion using cobalt(II) acetylacetonate. These precursors resulted in the formation of oligomers **62^−^a**, **b**, **c** (Figure 10). In the second synthetic route, the synthesis started with diiodo-substituted cobaltacarborane, and a zinc derivative of the 1,4-dibutyl-2,5-diethynylbenzene linker was prepared. The reaction between the complex and the zinc derivative with a palladium(0) catalyst resulted in the formation of a mixture of oligomers and polymers **63^−^** (Figure 10) [120]. The mentioned synthetic strategy was also later used in the synthesis of bis(ferrocenylethynyl)cobalt bis(dicarbollide) anion **64^−^** (Figure 10). In this case, the organozinc derivative of the 1,4-dibutyl-2,5-diethynylbenzene was replaced with an ethynylferrocenyl zinc reagent. This reagent was used in a Pd-catalyzed Kumada cross-coupling reaction with B(8,8′)-diiodo bis(dicarbollide) [121].

**Figure 11:**
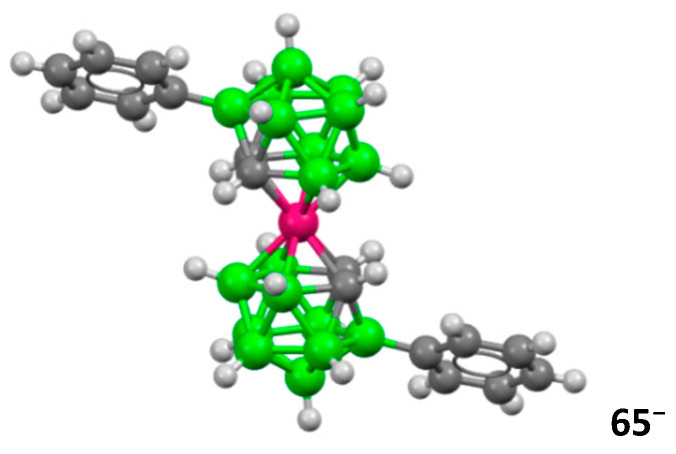
Drawing of the molecular structure of **65^−^** reported in ref. [122]. Boron atoms are drawn in green, carbon in gray, and cobalt in rose color.

Shmaľko, Sivaev, Bregadze et al. reported the synthesis of phenyl disubstituted anion **1^−^** in position B(6,6′) of the cage (**65^−^**), which was prepared from the corresponding 3-Ph-dicarba-*closo*-dodecaborane derivative (Figure 11) [122]. Additionally, a wider series of (9,9′, 12,12′) tetrasubstituted cobalt bis(dicarbollide) derivatives containing bromo, alkyl, and phenyl groups were prepared using these pathways [123]. These compounds have substituents at unusual sites located in the upper skeletal pentagonal plane, which is non-adjacent to the cobalt atom.

**Figure 12:**
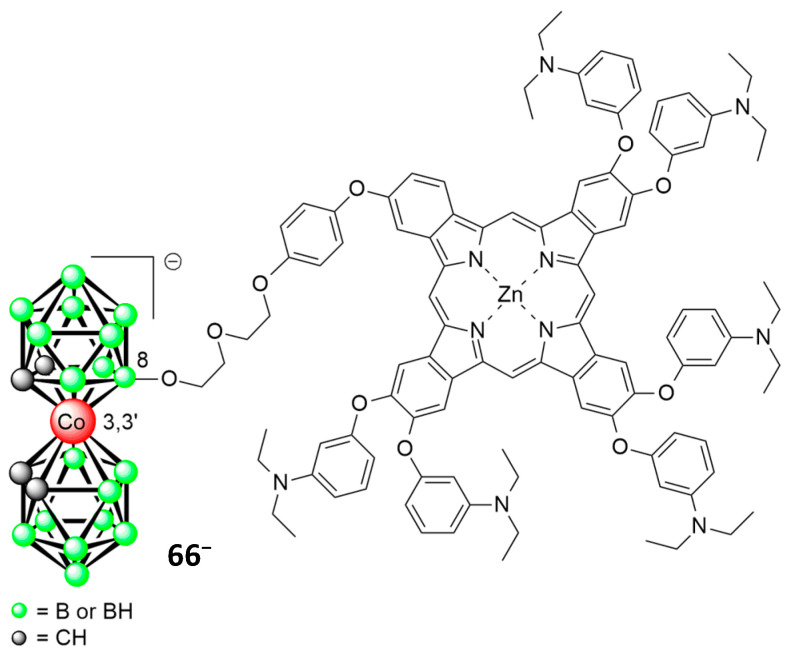
Chemical structure(s) of 2,3,9,10,16,17-Hexakis(3-dietylaminophenoxy)-23-[3,3′-Co(1,2-C_2_B_9_H_10_)_2_]phthalocyaninato Zinc(II) (**66^−^**).

From an electrochemical perspective, Nar et al. reported the synthesis and characterization of a new unsymmetrical zinc phthalocyanine with a cobalt bis(dicarbollide) unit **66^−^** (Figure 12). In the synthesis of this compound (Pc), a statistical condensation approach was employed using two different precursors. Phthalonitrile derivatives 4,5-bis(3-diethylaminophenoxy)-phthalonitrile and 4-hydroxyphenoxyphthalonitrile were used as starting materials. The metal-mediated cyclotetramerization of these compounds with ZnCl_2_ produced a mixture of symmetrical and unsymmetrical analogs of phthalocyanine, which were separated using column chromatography. The compound **66^−^** was then obtained by reacting Pc with the oxonium derivative of cobalt bis(dicarbollide) in acetone under reflux. By introducing diethylaminophenoxy moieties to the phthalocyanine fragments, high solubilities in organic solvents were achieved, enabling electropolymerization on the electrode surface [124].

**Figure 13:**
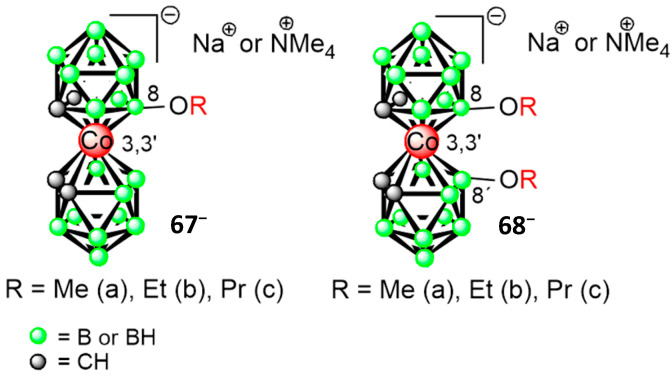
Structural formulas of compounds of various alkoxy derivatives of cobalt bis(dicarbollide) (**67^−^**, **68^−^**).

The synthesis of alkoxy derivatives via the alkylation of hydroxy derivatives of cobalt bis(dicarbollide) remains limited [99,125,126]. Stogniy et al. performed the reactions of monohydroxy derivatives of cobalt bis(dicarbollide) with methyl, ethyl, or propyl iodide in the presence of sodium hydride, resulting in the formation of alkoxy derivatives [8-*R*-O- (1,2-C_2_B_9_H_10_)_2_-3,3′-Co(III)]^−^ (R = Me, Et, Pr) (**67^−^a**, **b**, **c**; Figure 13) in high yields. Similarly, it was possible to obtain symmetrically substituted 8,8′-dialkoxy derivatives (**68^−^a**, **b**, **c**; Figure 13) [8,8′-(*R*-O)_2_-(1,2-C_2_B_9_H_10_)(1′,2′-C_2_B_9_H_10_)-3,3′-Co(III)]^−^ (R = Me, Et, Pr) either directly or using a two-step synthesis depending on the amount of NaH [126].

**Scheme 17:**
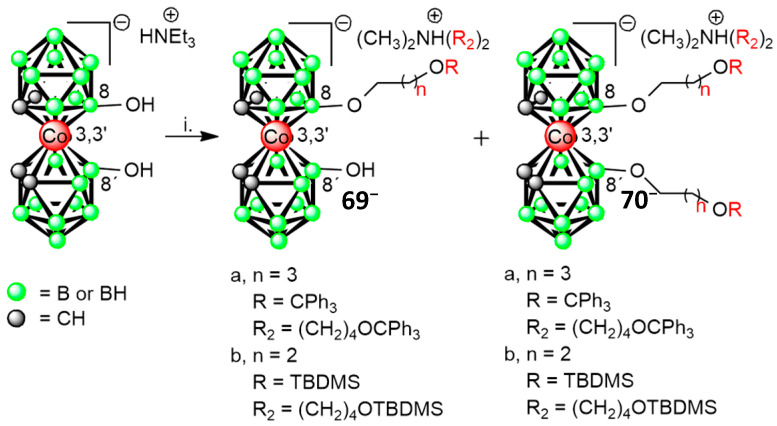
Alkylation of [8,8′-(OH)_2_-(1,2-C_2_B_9_H_10_)_2_-3,3′-Co(III)]^−^; i. NaH, alkylating agents in DMF, heating [93].

Śmiałkowski et al. (2023) focused on the oligofunctionalization of cobalt bis(1,2-dicarbollide) using hydroxyalkyl ligands to maximize the distance between functional groups and the cluster core. The study focused on functionalizing cobalt bis(1,2-dicarbollide) at boron atoms 8 and 8′ proceeding via the direct alkylation of hydroxy groups. The cobalt bis(1,2-dicarbollide) was converted into formula [8,8′-(OH)_2_-(1,2-C_2_B_9_H_10_)_2_-3,3′-Co(III)]^−^ using aqueous sulfuric acid, following the procedure described by Plešek et al. [127]. The authors employed different alkylating agents (4-trityloxybutyl 4-methylbenzenesulfonate and 3-bromopropoxy)(tert-butyl)dimethylsilane) for protective substitution of OH groups, however, the complete alkylation of both hydroxyl groups was not achieved, resulting in a mixture of mono- and disubstituted products (**69^−^a**, **b**, **70^−^a**, **b**; Scheme 17) [93], which had to be separated.

**Figure 14:**
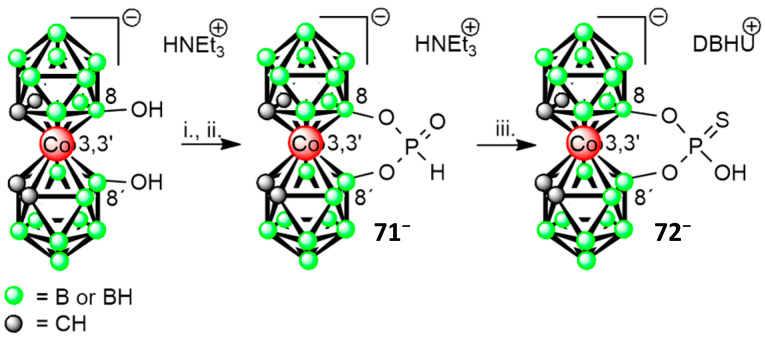
Preparation of H-phosphonate (**71^−^**) and thiophosphate (**72^−^**) esters of [8,8′-(OH)_2_-(1,2-C_2_B_9_H_10_)_2_-3,3′-Co(III)]^−^; i. PCl_3_, imidazole, Et_3_N in THF; ii. H_2_O; iii. S_8_, DBU in MeOH. DBU = 1,8-diazabicyclo(5.4.0)undec-7-en [93].

The same research group investigated the functionalization of boron atoms 8 and 8′ in 8,8′-O,O-[cobalt bis(1,2-dicarbollide)] phosphorothioate (**72^−^**) using *S*-alkylation. The synthesis of compound **72^−^** involved a two-step procedure (Figure 14). Initially, they converted the 8,8′-dihydroxy-derivative into 8,8′-bridged 8,8′-O,O-[cobalt bis(1,2-dicarbollide)] H-phosphonate acid ester (**71^−^**) using tris(1H-imidazol-1-yl)phosphine and the in situ hydrolysis of the resultant imidazolide. Subsequently, they treated H-phosphonate acid ester **72^−^** with elemental sulfur in anhydrous methanol and with a strong organic base present in the reaction mixture [93].

**Figure 15:**
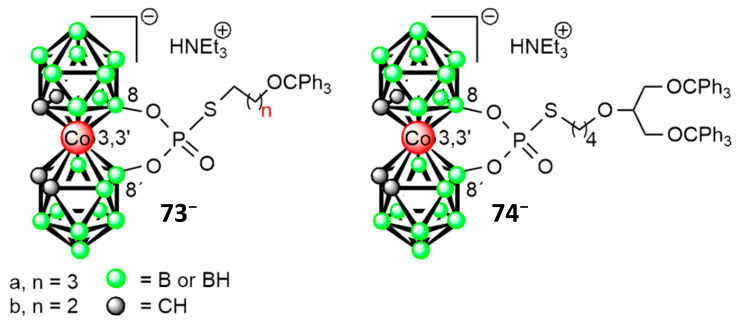
Structural formulas of *S*-alkylated phosphorothioates compounds 8,8′-bridged [8,8′-*μ*-O_2_P(O)S(CH_2_)_n_OCPh_3_-(1,2-C_2_B_9_H_10_)_2_-3,3′-Co(III)]HNEt_3_ (**73^−^**) and 8,8′-bridged {8,8′-*μ*-O_2_P(O)S[(CH_2_)_4_OCH(CH_2_OCPh_3_)_2_]-(1,2-C_2_B_9_H_10_)_2_-3,3′-Co(III)}HNEt_3_ (**74^−^**) [93].

Furthermore, the *S*-alkylation of compound **72^−^** could be performed using both linear and branched alkylating agents with hydroxyl functions protected by a trityl group. The nucleophilic properties of sulfur played a beneficial role in this alkylation reaction, resulting in high yields of the alkylated products **73^−^a**, **b**, and **74^−^** (Figure 15). Interestingly, derivatives of bridged 8,8′-µ-O,O-[cobalt bis(1,2-dicarbollide)] phosphate, which have an O,O-phosphate bridge instead of a phosphorothioate group, have not been previously described. This distinction in reactivity could be attributed to the “metallacarborane effect”, which leads to the lower nucleophilicity of the phosphorus center and oxygen compared to sulfur [93].

**Figure 16:**
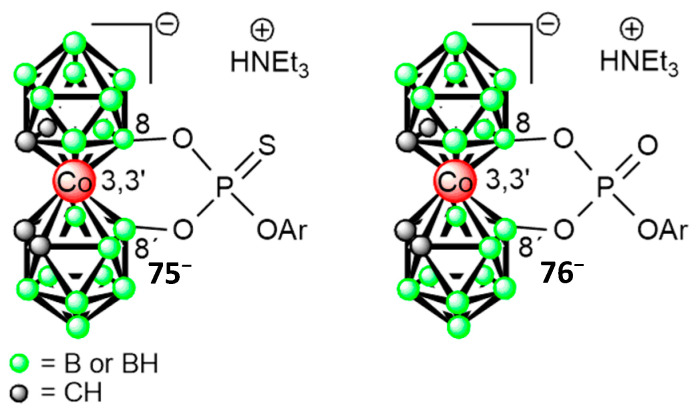
Chemical structures of [8,8′-*μ*-O_2_P(S)OC_6_H_4_NO_2_-(1,2-C_2_B_9_H_10_)_2_-3,3′-Co(III)]^−^ (**75^−^**) and [8,8′-*μ*-O_2_P(O)OC_6_H_4_NO_2_-(1,2-C_2_B_9_H_10_)_2_-3,3′-Co(III)]^−^ (**76^−^**); Ar = 4-nitrophenyl [128].

In the study of Sardo et al., bridged thiophosphate **75^−^** and its oxo-analogue **76^−^** (Figure 16) were synthesized by reacting the B(8,8′)-dihydroxy cobalt bis(dicarbollide) derivative with the corresponding dichloride esters in pyridine at room temperature. The remarkable stability of O-(4-nitrophenyl)phosphorothioate and phosphate esters of cobalt bis(dicarbollide) ion under alkali conditions was observed. The study demonstrated that metallacarboranes exhibit a significantly decreased reactivity of phosphorus-bridged metallacarborane esters when compared with organic analogues. This reduction in reactivity is attributed to the inductive effect and electrostatic shielding of the phosphorus atom by the metallacarborane, which weakens the electrophilicity of the phosphorus and hinders the approach of negatively charged nucleophiles. These effects, along with steric hindrance and the limited flexibility of the six-membered ring (considering the involvement of phosphorus, oxygens, boron, and cobalt atoms), are referred to as the metallacarborane effect. The metallacarborane effect has a significant influence on the reactivity of phosphate and phosphorothioate functional groups bonded via a bridge, while the thio- effect has a minor role in this respect [128].

**Figure 17:**
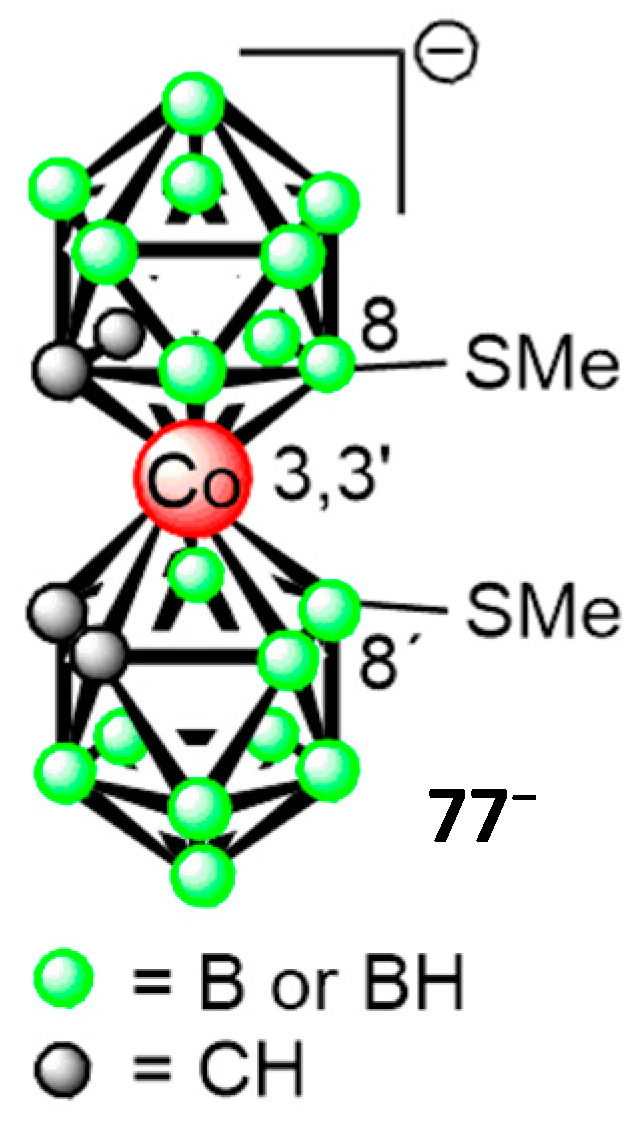
Chemical structures of [8,8′-(MeS)_2_-(1,2-C_2_B_9_H_10_)_2_-3,3′-Co(III)]^−^ (**77^−^**).

The synthesis of 8,8′-bis(methylsulfanyl) derivatives of cobalt bis(dicarbollide) **77^−^** was achieved through two different strategies (Figure 17). The first method involved the substitution of hydrogen atoms in the parent cobalt bis(dicarbollide) molecule. A dithiol derivative was obtained via the reaction of the parent molecule with carbon disulfide, followed by acidic hydrolysis. The alkylation of the dithiol derivative with various alkylating agents provided alkylthio derivatives. However, this method could not be extended to other transition-metal bis(dicarbollides) or to the preparation of complexes with asymmetrically substituted dicarbollide ligands [129,130,131,132]. The second strategy involved the synthesis of the corresponding carborane ligand in the first step followed by metal insertion. This approach allows for the synthesis of transition-metal bis(dicarbollide) complexes with substituents at different positions of the dicarbollide ligands. A symmetrically substituted methylsulfanyl derivative of cobalt bis(dicarbollide) was synthesized by reacting the corresponding carborane ligand with CoCl_2_ in a strongly alkaline solution. The products were isolated in good yields [133,134].

**Figure 18:**
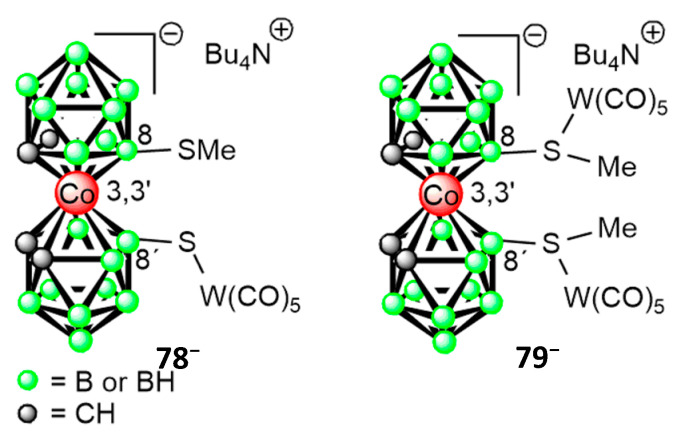
Chemical structures of {(CO)_5_W[*μ*-8,8′-(MeS)_2_-(1,2-C_2_B_9_H_10_)_2_-3,3′-Co(III)]}^−^ (**78^−^**) and {[(CO)_5_W]_2_[*μ*-8,8′-(MeS)_2_-(1,2-C_2_B_9_H_10_)_2_-3,3′-Co(III)]}^−^ (**79^−^**).

Methylsulfanyl derivatives of carboranes have been shown to form stable tetra- and pentacarbonyltungsten complexes [135,136]. Timofeev et al. hypothesized that the reaction between the 8,8′-bis(methylsulfanyl) derivatives of cobalt bis(dicarbollide) and (MeCN)_3_W(CO)_3_ results in the formation of a chelate complex {(CO)_4_W[*μ*-8,8′-(MeS)_2_-(1,2-C_2_B_9_H_10_)_2_-3,3′-Co(III)]}^−^, involving the rotation of the dicarbollide ligands from *transoid*- to *cisoid*-conformation. However, the reaction yielded a mixture of pentacarbonyl complexes (**78^−^**, **79^−^**; Figure 18) instead [137]. The 8,8′-bis(methylsulfanyl) derivatives of cobalt bis(dicarbollide) can also form chelate complexes with various transition metals such as copper, silver, palladium, and rhodium. This complexation process causes a conformational change from the *transoid*- to the *cisoid*-form. The transition between these conformations is reversible, and it can be reversed by removing the external transition metal using stronger ligands or precipitating agents. This reversible conformational change may offer potential for the design of coordination-driven molecular switches based on transition metal bis(dicarbollide) complexes [60].

**Figure 19:**
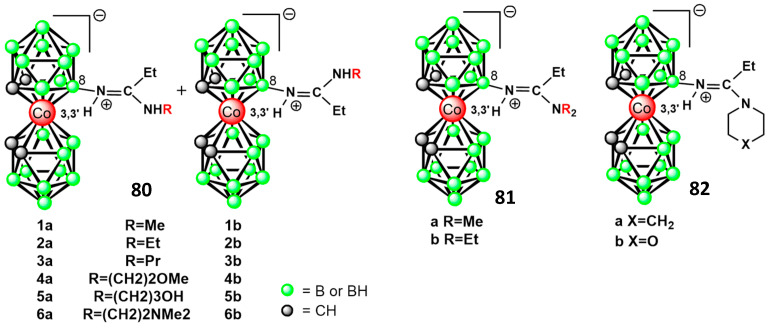
Schematic structures of [8-EtC(NH*R*)=HN-(1,2-C_2_B_9_H_10_)_2_-3,3′-Co(III)] (**80**), [8-EtC(N*R*_2_)=HN-(1,2-C_2_B_9_H_10_)_2_-3,3′-Co(III)] (**81**), and [8-EtC(NC_4_H_8_*X*)=HN-(1,2-C_2_B_9_H_10_)_2_-3,3′-Co(III)] (**82**).

Amidines are nitrogen analogues of carboxylic acids and esters and they are also important organic compounds widely used in the synthesis of various heterocycles and as pharmacophores in biologically active compounds. Bogdanova et al. studied the nucleophilic addition reactions of primary and secondary amines to the highly activated B–N^+^≡C–R triple bond of the propionitrilium derivative **80**. Several new amidines, based on metallacarboranes, were successfully synthesized (**81**, **82**; Figure 19). It was observed that the reactions with primary amines produced mixtures of *E*- and *Z*-isomers, while the reactions with secondary amines selectively yielded *E*-isomers [138].

### 4.1. Cobalt Bis(dicarbollide) as Extraction Agent

The field of radionuclide extraction using the cobalt bis(dicarbollide) ion was covered in two book chapters by Rais et al. (2004) [9], Grűner et al. (2012) [10], and a ligand designed for f-elements by Leoncini et al. (2017) [139]. Halogenated cobalt bis(dicarbollide) and their derivatives play a significant role in the extraction and separation of radioactive isotopes from nuclear waste, especially in the processes related to spent nuclear fuel (SNF) reprocessing and waste management. Due to their remarkable selectivity for cesium, strontium (with PEGs as synergists), and even lanthanides/minor actinides (either in synergic mixtures or after chemical modification), they have been shown to serve as a valuable tool for reducing nuclear waste volume and assisting in environmental remediation during nuclear accidents. Ongoing research in the field of radiochemistry focuses on cobalt bis(dicarbollide), with the aim of enhancing its extraction efficiency and optimizing its performance for various applications in nuclear waste treatment and management [140,141,142].

In 2017, Leoncini et al. published a review paper on extraction agents, summarizing developments in the ligand design, optimization, and extraction properties for the liquid–liquid extraction of f-elements from nuclear waste [139]. The following discussion will primarily highlight scientific papers published after the aforementioned review work.

**Chart 1:**
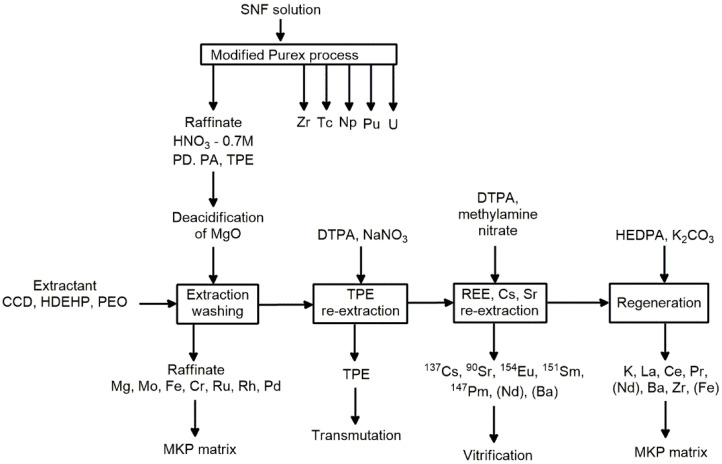
The flow sheet for extraction of TPE from weakly acidic raffinate.

A study conducted by Shishkin et al. (2020) reported the use of different extractive agents in the separation of elements present in weakly acidic raffinate. The study demonstrates that the extractive agent chlorinated cobalt dicarbollide (CCD) and di-2-ethylhexyl phosphoric acid (HDEHP) can effectively extract americium and europium from the solution, while Cs, Zr, U, Mo, and Fe are also extracted. Strontium and chromium remain in the raffinate. This extractive system also facilitates the separation of rare earths (REE) and transplutonium elements (TPE) with good separation factors (β). Urea is found to enhance the separation of REE and TPE in the extractive system. A scheme (see below; Chart 1) for the extraction of TPE from weakly acidic raffinate using the CCD + HDEHP extractive agent is presented, which reduces the volume of solutions to be evaporated and minimizes the use of complexating agents and auxiliary substances [143].

In 2022, this research group presented the efficiency of a synergic mixture comprising CCD, HDEHP, and polyethylene oxide (PEO) in separating cesium, strontium, REE, and TPE from weakly acidic raffinate. Adjusting the raffinate’s acidity with magnesium oxide enhances the extraction of cesium, strontium, americium, and europium. The nitric acid concentration affects the distribution coefficients of REE and TPE. The use of diethylenetriaminepentaacetic acid (DTPA) and sodium nitrate allows for the effective separation of REE and TPE. The addition of methylamine nitrate enables the separation of radiotoxic lanthanides. The regeneration of the extractant is possible using solutions of potassium carbonate and HEDPA. The deep partitioning scheme offers advantages in element separation and minimizes vitrified HLW volume [144].

**Figure 20:**
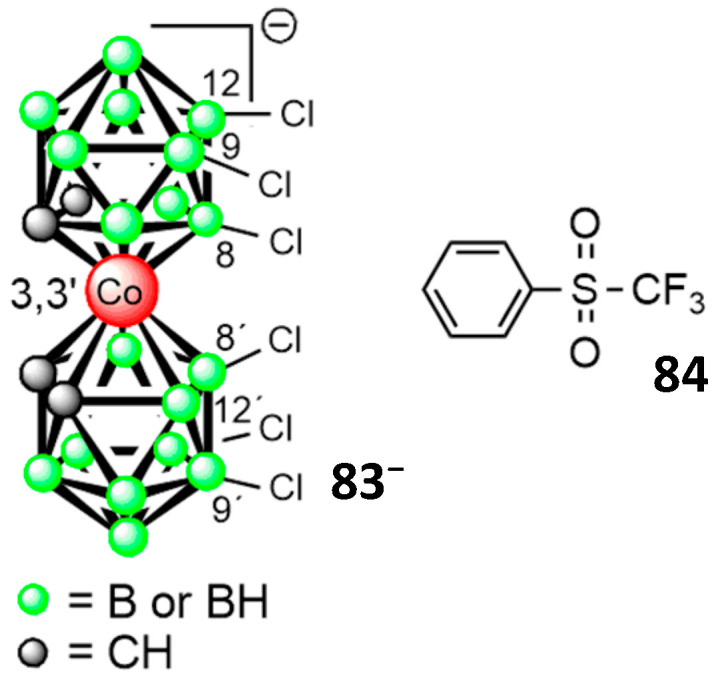
Chemical structures of [8,8′,9,9′,12,12′-Cl_6_-(1,2-C_2_B_9_H_10_)_2_-3,3′-Co(III)]**^−^** (**83^−^**) and phenyl trifluoromethyl sulfone (**84**).

The kinetics of Cs^+^ and Sr(II) extraction and back-extraction were investigated using hexachlorinated cobalt bis(dicarbollide)/polyethylene glycol-400/FS-13 and a solvent system consisting of sulfone FS-13 (**83^−^**, **84**; Figure 20) in batch experiments. Cs^+^ extraction was relatively fast, reaching a steady state within 10 s, while stripping took about 50 s. Surprisingly, Sr(II) exhibited slower extraction kinetics compared to its back-extraction, contrary to common trends. In micromixer settler experiments, both Cs^+^ and Sr(II) extraction and stripping were improved using guanidine carbonate and diethylenetriamine pentaacetic acid (DTPA). The microbore-based helix provided short residence times, achieving quantitative extraction and back-extraction of Cs^+^, showing significantly faster kinetics than batch mixing. The mini-settler used in the experiments demonstrated effective settling, despite the role reversal of phases. Overall, the study showcased the kinetics of Cs^+^ and Sr(II) extraction, emphasizing the advantages of microchannel mixers over batch mixing methods [145].

### 4.2. Cobalt Bis(dicarbollide) as Potentiometric Membrane Sensors

**Figure 21:**
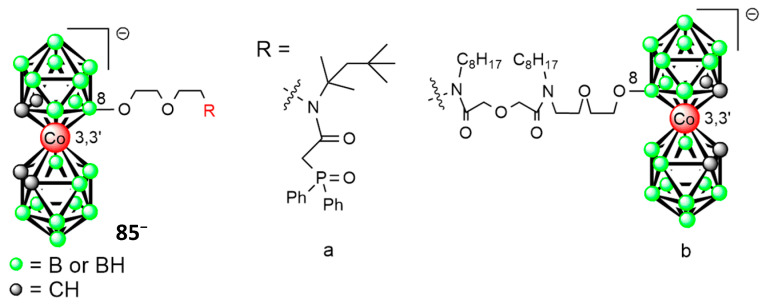
Chemical structures of [8-*R*(C_2_H_4_O)_2_-(1,2-C_2_B_9_H_10_)(1′,2′-C_2_B_9_H_11_)-3,3′-Co(III)]^−^ (**95^−^**) (2-(diphenylphosphoryl)acetyl)(2,4,4-trimethylpentan-2-yl) (**a**) and *N-R-N,N′*-dioctyl diglycolamide[8-(C_2_H_4_O)_2_-(1,2-C_2_B_9_H_10_)(1′,2′-C_2_B_9_H_11_)-3,3′-Co(III)]^−^ (**b**).

The sensitivity of novel sensors to single-, double-, and triple-charged ions was studied by Khaydukova et al., along with their selectivity. The sensor sensitivity varied depending on the type of ionophore used, either TODGA or CMPO (*N,N,N′,N′*-tetraoctyl diglycolamide or di-phenyl-*N,N*-di-*i*-sobutylcarbamoylmethylene phoshine oxide). In the first case, the ionophore was either covalently bound to the cage or used in a synergic mixture with chlorinated cobalt bis(dicarbollide) ion in the later studied system (**85^−^a**, **b**, Figure 21). For single-charged ions, CD-bonded sensors showed sensitivity to K^+^. Double-charged ions exhibited good sensitivity to Ca(II) with TODGA-based sensors. The highest sensitivity was observed for Pb(II) ions, but sensitivity to Zn(II), Cd(II), and Cu(II) species remained low. Triple-charged ions (REEs) displayed sensitivity correlating with atomic number, particularly with TODGA-based sensors. The study also explored the effects of plasticizers and chlorinated cobalt bis(dicarbollide) concentration on sensitivity and selectivity. Sensors with bonded ligands were found to be suitable for multisensor systems for the simultaneous determination of several cations [146].

Chaudhury et al. (2014) investigated the electro-driven selective transport of Cs^+^ from low acidic/neutral and high-level waste solutions through ion-exchange membranes. The main aim was to reach high Cs^+^/Na^+^ selectivity. The study, which measured potential drop and membrane resistance, demonstrated that cellulose-triacetate-based polymer inclusion membranes proved to have advanced properties compared to conventional types of membranes. The polymer inclusion membrane containing hexachlorinated cobalt bis(dicarbollide) efficiently transported Cs^+^ in an electric field with high selectivity, making it energy-efficient and eco-friendly for nuclear waste treatment [147]. In 2018, the same research group published another study on the electro-driven transport of Cs^+^ using chlorinated cobaltbis(dicarbollide) loaded membranes. The authors show that hollow-fiber-supported liquid membrane (HFSLM) can effectively treat large volumes of waste solutions with high Cs^+^ selectivity, outperforming PIM in terms of transport rate and decontamination factor. The stability of the membrane against carrier leaching is also confirmed. Furthermore, the text provides current profiles and efficiencies for Cs+ transport from both neutral and acidic feed solutions. It emphasizes the time needed for complete Cs^+^ transport in highly acidic solutions. The successful transport of Cs^+^ from environmental samples is also reported, demonstrating the selective removal of Cs^+^ over Na^+^, K^+^, and calcium(II) [148].

Further information on the significance of metallacarboranes for producing potentiometric membrane sensors, to monitor amine-containing drugs and other nitrogen-containing molecules, is reported in a mini-review from Stoica et al. published in 2022 [14].

## 5. Recent Medicinal Applications

Polyhedral boron clusters have garnered significant attention as unconventional inorganic pharmacophores [66,149,150,151,152,153,154,155,156,157,158]. In recent years, there has been a growing focus on exploring the applications of cobalt bis(dicarbollide) ion in medicinal chemistry, particularly in the design of biologically active compounds with antiviral [31,159], antibacterial [35], and anticancer properties [32,160,161,162] and carriers for BNCT [38,163]. This is a binary treatment method for cancer that relies on the selective accumulation of boron compounds in tumor cells, followed by irradiation with a thermal neutron flux. The irradiation leads to the selective destruction of tumor cells while minimizing damage to surrounding normal tissues. To achieve the successful development of BNCT, it is crucial to reach the selective delivery of a large load of ^10^B nucleus to tumor cells and ensure high boron accumulation (by uptake), while maintaining a low concentration of boron in the surrounding normal tissues [69,164,165,166].

To enhance the functionality of parent anion, the nucleophilic cleavage of a cyclic ether ring is employed as the building block for conjugation with biomolecules. Additionally, the regioselective click reaction, specifically the [3 + 2] cycloaddition of alkynes to azides [108,167], is a useful method for the modification of biomolecules with boron clusters. The resulting boron-containing triazole linkage mimics the stability and properties of the peptide bond [108]. The following paragraphs discuss the biomedical versatility of cobalt bis(dicarbolide) anion derivatives.

### 5.1. Recent Studies on the Parent Cobalt Bis(dicarbollide) Ion

Advancements in the emerging medicinal chemistry of the cobalt bis(dicarbollide) ion have triggered recent interest in its interactions with cells and the phenomena that occur in aqueous solutions. Indeed, the solution of the behavior of this ion is unusual and parallels that of other bulky surface-active inorganic ions denoted as chaotropes or even superchaotropes [168,169]. Understanding these properties plays a key role in the practical aspects of drug design and the pharmaceutical formulations of active compounds.

Moreover, from the perspective of practical synthesis and biological properties, the effects of charge, interactions with cations, solubility of the particular salt in selected solvents, cage solvation, and the tendency to self-assemble correspond to factors that have not been fully understood and warrant further investigation.

Self-assembly in an aqueous solution, host–guest properties, and the interactions of boron cluster compounds, inclusive of cobalt bis(dicarbollide) ion with biomolecules, are the subject of a recent review article published by Cebula et al. (2022) [170]. Here, we briefly describe and comment on the recent results of physicochemical and biological studies.

In 2006, Matějíček et al. made a significant step forward in understanding the physicochemical phenomena in aqueous solutions of the ion **1^−^**. The aggregation of the sodium salt of cobalt bis(dicarbollide) (Na**1**) and other metallacarboranes in aqueous solutions was described [171]. Recent studies on the Na**1** revealed a two-step aggregation process [172,173,174]. The first step involves the formation of small aggregates with a pentamer structure, driven by favorable enthalpy and positive entropy. The second step leads to larger aggregates with more counterion binding and exothermic enthalpy. The proposed model suggests that pentamers are stabilized by two embedded Na^+^ counterions, a hypothesis supported by quantum chemistry calculations [175].

Water-soluble cobalt bis(dicarbollide) salts, including H**1**, Li**1**, K**1**, and Cs**1**, exhibit an aggregation tendency, with similar critical aggregation concentration (CAC) values and aggregation numbers, suggesting a common aggregation mechanism. However, the aggregation behavior of Cs**1** differs due to the large size of the cesium cation, making it unable to fit into the space between cobalt bis(dicarbollide) clusters. Molecular dynamics simulations were used to investigate the aggregation behavior of Na**1** in explicit water. While the simulation snapshots showed changes in rotational isomerism, the fixed *cisoid*-cobalt bis(dicarbollide) clusters model provided more realistic results with roughly uniform pentamers forming around CAC. The simulation results are in agreement with the experimental evidence, and the presence of pentamers as the most probable pattern of the cobalt bis(dicarbollide) aggregates in solutions around CAC was confirmed. Additional investigation is required to gain a comprehensive understanding of the counterions’ role in the aggregates of various cobalt bis(dicarbollide) salts and other metallacarborane analogues [175].

The study by Merhi et al. (2020) delved into how the cobalt bis(dicarbollide) interacts with an octyl-glucopyranoside surfactant (C8G1), leading to the formation of mixed aggregates. Depending on the concentrations of both substances, various types of assemblies were formed. At low cobalt bis(dicarbollide) content, cobalt bis(dicarbollide) vesicles and monomers coexisted. The addition of C8G1 in a monomeric form disrupted cobalt bis(dicarbollide) vesicles, forming cobalt bis(dicarbollide)-C8G1 nano-assemblies through hydrophobic interactions. At low cobalt bis(dicarbollide) content and high C8G1 concentrations, the ion adsorbed to the surface of C8G1 micelles, stabilizing them and reducing the critical micellar concentration of C8G1. However, with high cobalt bis(dicarbollide) content, the ion disrupted C8G1 micelles and penetrated the micellar surface. At higher cobalt bis(dicarbollide)/C8G1 ratios, assemblies similar to cobalt bis(dicarbollide) micelles containing solubilized C8G1 were formed. The study unveiled the superchaotropic behavior of cobalt bis(dicarbollide), where it spontaneously adsorbed onto C8G1 micelles, akin to other nanometric ions. This behavior was more pronounced at high C8G1 concentrations, superseding the hydrophobic effect observed at low concentrations. Cobalt bis(dicarbollide) and its derivatives demonstrated surfactant properties and hydrophobic characteristics, enabling them to cross biological membranes, including cell membranes, with potential applications in the pharmaceutical field [176].

Zaulet et al. (2018) explored the self-assembly of cobalt bis(dicarbollide) in water and the role of intermolecular interactions in forming aggregates. Salts of cobalt bis(dicarbollide) with alkali metal cations of Na^+^, K^+^, and Li^+^ of cobalt bis(dicarbollide) were prepared using cation exchange, and their crystal structures revealed the presence of dihydrogen bonds. These dihydrogen bonds were responsible for the formation of stable supramolecular entities in water, avoiding direct contact with surrounding water molecules. The intermolecular B-H_B_ ^δ−^ …^δ+^H_C_-C interactions played a crucial role in the self-assembly process. The coordination of counter ions also influenced the self-assembly, as evidenced by the formation of a layered 2D structure in the crystal structure of [Na(H_2_O)_4_][Co(C_2_B_9_H_11_)_2_] [177].

Fernandez-Alvarez et al. (2019) focused on examining how presence of cobalt bis(dicarbollide) ion affect solution behavior and morphology of star-like polyelectrolyte micelles with a fixed number of arms. The two hydrophobic counterions consisted of cobalt bis(dicarbollide) anion, and the polycationic copolymeric micelles, which had polycationic star-like structures with frozen cores. By conducting computer simulations and experiments, they observed that the gradual addition of cobalt bis(dicarbollide) caused the micelles to precipitate at specific ratios, irrespective of the micelle’s ionization level. In contrast, the addition of an indifferent electrolyte like NaCl did not produce the same effect. The simulations revealed that hydrophobic counterions altered the ionization profile of micelles, causing the formation of compact domains. The precipitation mechanisms differed based on ionization levels: less ionized stars collapsed into a single globule, while fully ionized stars formed an infinite gel network with counterion pearls. An NMR analysis confirmed the collapsed domains due to hydrophobic counterions. The findings could have applications in pH-controlled drug delivery with hydrophobic ionic solutes in polyelectrolyte-based nanostructures [178].

Traditionally, bulky monovalent cations and alkylammonium salt are used to precipitate the cobalt bis(dicarbollide) anion, but divalent cations are of particular biological relevance. There is a study by Zaulet et al. (2021) that explores the use of biocompatible divalent cations, such as Ca(II), Mg(II), and Fe(II), to isolate the cobalt bis(dicarbollide) anion. Ca(II) and Mg(II) are classified as hard Lewis acids, which means they form aqua ions when water is present during the synthesis process. In this study, all solid Ca(II) and Mg(II) salts examined were found to contain coordinated water molecules, as detected by IR and TGA/DSC analyses. On the other hand, Fe(II) is considered a medium-hard Lewis acid, while Fe(III) is classified as a hard Lewis acid. When placed in acetone, Fe(II) and Fe(III) ions do not coordinate with the cobalt bis(dicarbollide) anion but instead become solvated by acetone [153].

Rak et al. (2010), for the first time, studied the deaggregation of the cobalt bis(dicarbollide) ion in aqueous solutions on a panel of hydrophobic HIV-Protease and NO synthase inhibitors. A broad series of modified cyclodextrins (CD), classical surfactants, and amphiphilic copolymers were tested with the aim of finding general biocompatible excipients. They found several hints such as Pluronic F-127, DIMEB, or PVP, which may serve as suitable excipients. The strong interaction of metallacarorane derivatives with Human Serum Albumin (HSA) was found to compete with the solubilizing action of these species. However, it was found that HSA can itself act as a solubilization agent [179].

In the study of Goszczyński et al. (2017), they examined the fluorescence quenching mechanism, binding constants, and binding modes of Bovine Serum Albumin (BSA) when exposed to various boron clusters. The results showed that metallacarboranes caused substantial fluorescence quenching, utilizing both dynamic and static mechanisms. Metallacarboranes displayed strong binding constants, primarily engaging in hydrophobic interactions. At low stoichiometry, they first exhibited specific interactions with BSA within the hydrophobic cavity. However, at high stoichiometry, they also displayed non-specific interactions with the protein surface. Cobalt bis(dicarbollide) ion had a significant impact on BSA, inducing changes in BSA’s *α*-helix content and forming stable complexes that affected BSA’s hydrodynamic size. These findings offered valuable contributions to the development of novel bioactive compounds incorporating boron clusters [180].

Assaf et al. (2019) studied in detail the supramolecular chemistry of the series of parent isomeric cobalt bis(dicarbollide) ions, and seven simply substituted derivatives with cyclodextrins in an aqueous solution, using NMR and UV-visible spectroscopy, MS, electrochemistry, and isothermal titration calorimetry techniques. The compounds were found to be strongly bound to cyclodextrins [181], in particular into *β*-CD and *γ*-CD cavities. The binding constants of the inclusion complexes reached values in the micromolar range and thus exceeded those of highly hydrophobic nanodiamonds and were comparable with perhalogenated dodecaborate clusters [168]. The translocation of several cobalt bis(dicarbollide) derivatives through the lipid bilayer was shown by using fluorescence monitoring in supramolecular tandem membrane assays. No damage of the bilayer was observed.

The research of Abdelgawwad et al. (2021) aimed to explore the light-induced stabilization of cobalt bis(dicarbollide) isomers in water, with potential applications in light-switchable surfactants for drug delivery. *Transoid*-cobalt bis(dicarbollide) showed higher stability in the excited state than *cisoid*-cobalt bis(dicarbollide). Photoexcitation in water promoted rotation to the *transoid*-form, creating a fast photoinduced rotation. The study also revealed non-radiative decay mechanisms affecting luminescence in 3D fluorescence experiments. Overall, the research provided insights into cobalt bis(dicarbollide)’s photochemical behavior and its potential for light-responsive surfactants in drug delivery [182].

A recent study by Chazapi, Diat, and Bauduin (2023) demonstrated that sodium salt of cobalt bis(dicarbollide) ion can act, due to its surfactant properties, as an efficient aqueous solubilizer of medium-chain alcohols (0.6 < log*P* < 1.5) and hydrophobic organic compounds, when the sodium salt is present in a solution in a monomeric form [183]. This relates to concentrations lying below its critical micelle formation. Mechanistically, the solubilization corresponds to the two-dimensional anisotropic growth of cobalt bis(dicarbollide)/butanol co-assemblies, whereas solubilization by the surfactant occurs via an isotropic swelling of micelles. Such co-assemblies with 2-butanol efficiently solubilize more hydrophobic compounds and dyes with log*P*_ov_ values from 0.6 to approximately 5.6. This seems to be an important result, which suggests good prospects for use in several fields of contemporary applications, in particular in formulations of lipophilic cobalt bis(dicarbollide) drugs, e.g., those dicluster inhibitors of HIV-Protease [165], lipophilic organic compounds such as aromatics, terpenoids, aldehydes, and ketones, and ethers used as fragrances, pharmaceutical active species, and dyes.

The penetration of a parent cobalt bis(dicarbollide) ion and its dihalogenated derivatives through lipid membranes was studied by Rokitskaya, Bregadze et al. (2017). The rates of penetration increased with the molecular weight of the halogen and the molecular volume of the anion. Thus, the parent ion and its fluorinated derivative exhibited the slowest transmembrane penetration compared to the compounds modified with heavier halogens. The authors expressed the hypothesis that this is connected with different conformations of the adsorbed species, which complicates the permeation due to the rotational component of the transmembrane diffusion [184].

Barba-Bon, Nau et al. (2022) demonstrated that both the isomers of parent cobalt bis(dicarbollide) ion and its halogenated and methylated derivatives are selective and highly efficient molecular carriers of impermeable hydrophilic oligopeptides through both artificial and cellular membranes. At the studied low micromolar concentrations of the carrier, no damage to the membrane was observed. The compounds were shown to transport both arginine- and lysine-rich peptides. Neither low-molecular-weight analytes, such as amino acids, nor neurotransmitters as well as neutral and anionic cargos (phalloidin and BSA) were co-transported in these experiments. U-tube experiments and electrophysiology establish that the transport is mediated by a molecular carrier mechanism and exclude alternative uptake pathways, such as channel or pore formation. It has been shown that the oligopeptides are delivered into the cytosol and nucleus by direct penetration [185].

Fink et al. (2023) conducted a comprehensive study to investigate the interaction between cobalt bis(dicarbollide) and DNA using various techniques. UV-Vis absorption spectroscopy, circular dichroism, linear dichroism, viscosity measurements, and differential scanning calorimetry all indicated that cobalt bis(dicarbollide) did not strongly interact with DNA. These methods showed no significant changes in the DNA absorption spectra, secondary structure, or intercalation of cobalt bis(dicarbollide) into DNA, suggesting no strong binding. Additionally, three other methods (isothermal titration calorimetry, equilibrium dialysis, and NMR) were used to detect potential cobalt bis(dicarbollide)-DNA complexes, but no strong interaction between cobalt bis(dicarbollide) and DNA was found. Regarding toxicity, cobalt bis(dicarbollide) showed moderate hemolytic activity towards anuclear cells (like red blood cells) and also demonstrated toxicity towards nuclear cells (various cell lines) at similar concentrations. The toxicity towards nuclear cells could potentially involve cell membrane disruption, but further investigation is needed to confirm this hypothesis. Thus, the study’s overall findings suggest that cobalt bis(dicarbollide) does not strongly bind to DNA and exhibits moderate toxicity towards both anuclear and nuclear cells [26].

### 5.2. Application of Carbon-Substituted Compounds

#### 5.2.1. Anticancer Compounds

##### Inhibitors of Carbonic Anhydrase IX Enzyme

Carbonic anhydrase IX (CA-IX) is a transmembrane zinc metalloenzyme that regulates pH in hypoxic tumors and promotes tumor cell survival. Its expression is associated with the occurrence of metastases and poor patient prognosis [186]. Carboranes and cobalt bis(dicarbollide) ions may act as highly potent inhibitors of this enzyme if the cage is substituted with a zinc binding group attached via an aliphatic pendant group. The progress in the field of boron cluster inhibitors forms the topic of a recently published review [32].

**Scheme 18:**
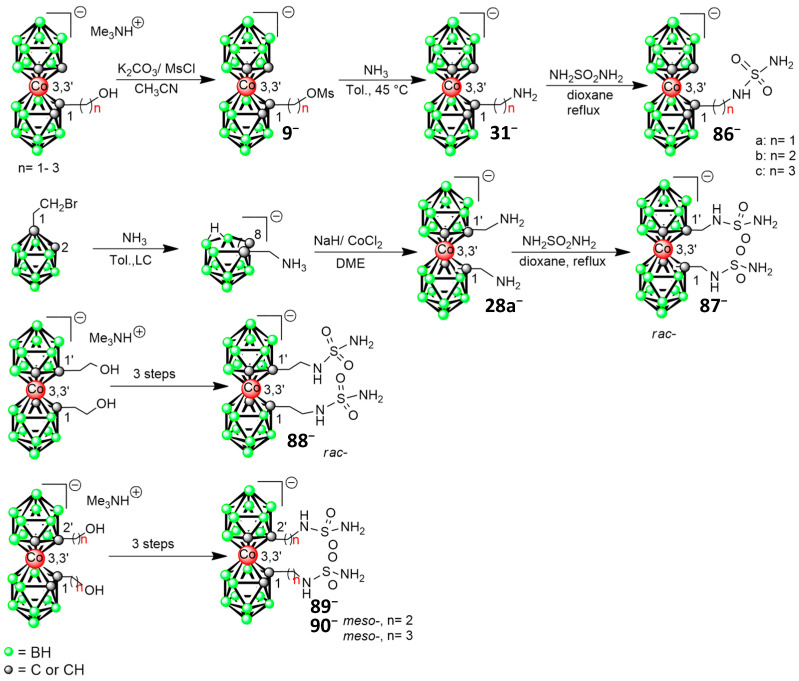
Synthetic procedures used for the synthesis of the pilot series of inhibitors based on modifications of the cobalt bis(dicarbollide) ion at carbon atoms with alkylsulfamido group [25,32].

The first series of highly specific and selective cobalt bis(dicarbollide)(**1^−^**) inhibitors of CA-IX, substituted either on the boron or on carbon sites by alkylsulfamide group(s), was reported by Grűner, Hajdúch, and Řezáčová et al. in 2019 [25]. A key step forward that enabled the rational design of inhibitors in this research was connected with the preparative availability of C-aminoalkyl-substituted metallacarboranes with tunable linker length. The synthesis started with a three-step process based on cobalt bis(dicarbollide) described in Section 3.6.3, which we consider as the most reliable reaction pathways leading to C-alkylamines. In only one case, a different strategy was used consisting of indirect cobalt insertion i.e., cobalt insertion into [7-H_3_NCH_2_-C_2_B_9_H_11_] that resulted in corresponding metallacarborane amine [(1,1′-(NH_2_CH_2_-1,2-C_2_B_9_H_10_)_2_-3,3′-Co(III)]^−^. The *rac*-isomer of the di(methyleneamino)-substituted sandwich was obtained as the only isolatable product, as verified by NMR and its X-ray structure (see Figure 2). The primary alkylamino groups were then converted to corresponding sulfamides upon heating with sulfamide in dioxane in the presence of K_2_CO_3_ (Scheme 18), providing inhibitors with one sulfamido group of formula [(1-H_2_NSO_2_NH-(CH_2_)_n_-1,2-C_2_B_9_H_10_)(1,2-C_2_B_9_H_11_)-3,3′-Co(III)]^−^ (**86^−^a**, **b**, **c**, n = 1–3). In addition, four compounds containing two alkylsulfamido groups were prepared: [*rac*-1,1′-(NH_2_CH_2_-1,2-C_2_B_9_H_10_)_2_-3,3′-Co(III)]-, [*rac*-1,1′-(NH_2_-S(O)_2_NH-C_2_H_4_-1,2-C_2_B_9_H_10_)_2_-3,3′-Co(III)]^−^ (**88^−^**), [*meso*-1,2′-(NH_2_-S(O)_2_NH-C_2_H_4_-1,2-C_2_B_9_H_10_)_2_-3,3′-Co(III)]^−^ (**89^−^**) and [*meso*-1,2′-(NH_2_-S(O)_2_NH-C_3_H_6_-1,2-C_2_B_9_H_10_)_2_-3,3′-Co(III)]^−^ (**90^−^**). The interactions of these compounds with the active site of CA-IX were explored on the atomic level using protein crystallography.

The inhibitory profile of the compounds was tested in vitro using the stopped-flow carbon dioxide hydration assay. Two selected derivatives **86^−^** and **88^−^**, that showed *sub*-nanomolar or picomolar *K*_i_ values and high selectivity for the tumor-specific CA-IX over cytosolic isoform CAII, were the subject of subsequent detailed biological tests. Both derivatives proved to have a time-dependent effect on the growth of multicellular spheroids of HT-29 and HCT116 colorectal cancer cells, and, in addition, facilitated the penetration and/or accumulation of doxorubicin into spheroids. The results from in vivo tests demonstrated that both inhibitors displayed low toxicity and showed promising pharmacokinetics and a significant inhibitory effect on tumor growth in syngeneic breast 4T1 and colorectal HT-29 cancer xenotransplants in mice. The results from preclinical studies indicate the promising potential of these compounds to be developed into drug-like forms and used in cancer therapy [25,32].

More recently, the synthetic routes to compounds substituted with sulfonamido groups have been developed. The sulfamido group is known to bind to the zinc atom in the active site more tightly than sulfamide, typically resulting in improved inhibitory activity compared to corresponding isostructural compounds with the sulfamide group. Even in the case of cobalt bis(dicarbollide) inhibitors, the substitution for the sulfonamide function resulted in an increase in inhibition activity by approximately one order of magnitude [75].

Several methods for the direct substitution of the cobalt bis(dicarbollide) cage with one alkylsulfonamido group were tested. These included the conversion of the respective mesyl esters to corresponding derivatives containing thiourea derivatives with the formation of the corresponding derivative [1-*X*-(CH_2_)_n_-(1,2-C_2_B_9_H_10_)(1′,2′-C_2_B_9_H_11_)-3,3′-Co(III)]^−^ (X = -SC(NH_2_)_2_, n = 2 and 3). The terminal thiourea group was then converted by oxidative chlorination in the second step to the corresponding sulfamoyl chloride group. Mild conditions were used using NaClO in a two-phase system at low temperatures and high dilution. However, even then it was impossible to avoid cage chlorination at the two most electron-rich boron (B8,8′) sites. These sulfamoyl chlorides were then subsequently converted, by reaction with aqueous NH_4_OH, to the sulfonamides of formula [1-H_2_NSO_2_-(CH_2_)_n_-8,8′-Cl_2_-(1,2-C_2_B_9_H_9_)(1′,2′-C_2_B_9_H_10_)-3,3′-Co(III)]^−^. Two compounds comprising ethylene and propylene connectors were prepared [75].

**Scheme 19:**
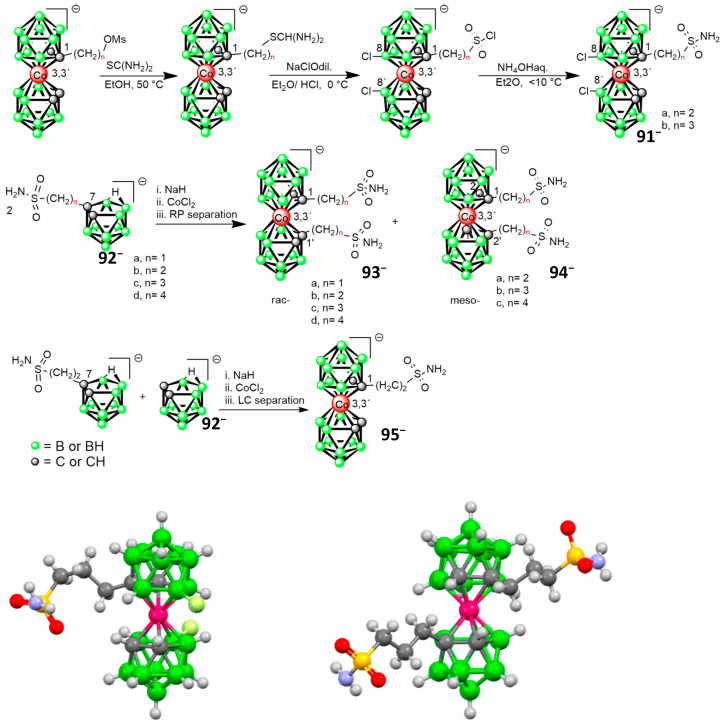
Synthetic methods used for the preparation of the series of alkylsulfonamido-substituted inhibitors. Schematic presentation of molecular structures of 8,8′-dichloro-sulfonamidopropyl and di(sulfonamidopropyl) cobalt bis(dicarbollide) ions, **91b^−^** and **93c^−^,** are shown in the bottom, from refs. [32,75]. Boron atoms are drawn in green, carbon in gray, cobalt in rose, sulfur in yellow, nitrogen in blue, oxygen in red, and chlorine in yellow-green color.

The primary synthetic routes, however, involved the use of an indirect approach. Thus, a cobalt atom was inserted into a substituted and NaH deprotonated 11-vertex *nido*-species of general formula [1-H_2_N_S_(O)_2_-(CH_2_)_n_-7,8-C_2_B_9_H_11_]^−^ (**92^−^a**, **b**, **c**, **d**, n = 1–4). The insertion of the Co(III) central atom provided the respective di(sulfonamidoalkyl) cobalt bis(dicarbollide) ions of formula [1,1′-(NH_2_-S(O)_2_-(CH_2_)_n_)_2_-(1,2-C_2_B_9_H_10_)_2_-3,3′-Co(III)]- (**93^−^a**, **b**, **c**, **d–104^−^a**, **b**, **c**) in moderate (for n = 1 and 4) or excellent yields (for n = 2 and 3). As follows from their origin, it was possible to synthesize mainly the disubstituted derivatives. The sandwich compounds were obtained as a mixture of *meso*- and *rac*-stereoisomers. Both isomers could be readily separated using preparative flash chromatography on a C18-RP column using a mobile phase consisting of aqueous methanol. Also, compounds from the monosubstituted series of formula [1-H_2_NSO_2_-(CH_2_)_n_-(1,2-C_2_B_9_H_10_)(1,2-C_2_B_9_H_11_)-3,3′-Co(III)]^−^ (**95^−^**) (Scheme 19, bottom) could be prepared using a similar indirect method of reacting the sulfonamide-substituted eleven-vertex ligand together with the parent unsubstituted [C_2_B_9_H_12_]^−^ ion, however, only in a low yield [75].

**Figure 22:**
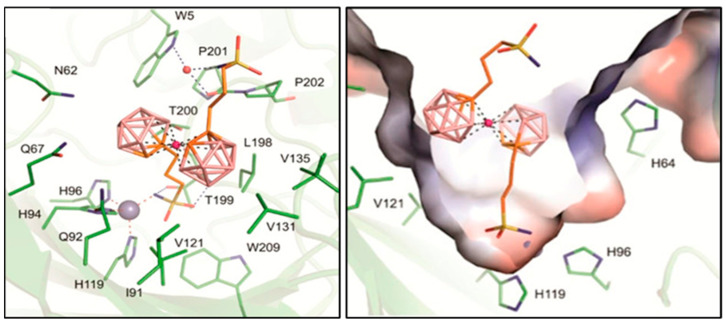
The binding mode of the compound **93c^−^** in the active site of CA-IX-mimic enzyme, according to refs. [75]. The boron atoms of the cage are drawn in light-pink, carbon in orange, cobalt in rose, sulfur in yellow, nitrogen in blue, oxygen in red, and zinc in the active site in gray color.

The inhibitory profile of the compounds was tested in vitro using the stopped-flow carbon dioxide hydration assay. To evaluate selectivity, the inhibition of two CA isoforms, the cancer-associated CA-IX and the widespread cytosolic CA-II isoform, was studied (Figure 22). The selectivity index (SI), expressed as a ratio of the two *K*_i_ values, is presented in Table 1. The whole series of compounds proved to serve as potent inhibitors of both CA isoforms, however, the values for CA-IX were significantly lower, which resulted in high selectivity [32,75].

Interestingly, comparing *K*_i_ values for **91a^−^** and **91b^−^**, the chlorination of the cage at B(8,8′) sites improved the inhibition of CA-II, rendering the activity towards CA-IX on the same level. However, this results in decreased selectivity towards CA-IX (Table 1).

A three-atomic pendant group appears to be of optimal length for CA-IX inhibition over the whole series. Thus, the most active inhibitor *rac*-**93c^−^** selectively inhibits CA-IX with a picomolar *K*_i_ value and also shows the highest S_I_ value 668. This is one order of magnitude better than for the corresponding *meso*-isoform. On the other hand, isomeric couples for other lengths of the linker do not show significant differences in their S_I_. Therefore, no conclusion about the effect of stereochemistry on CA-IX inhibition could be drawn over the whole series of inhibitors [32].

##### 1,8-Naphthalimides Derivatives, Analogues of Mitonafide, and Pinafide

**Figure 23:**
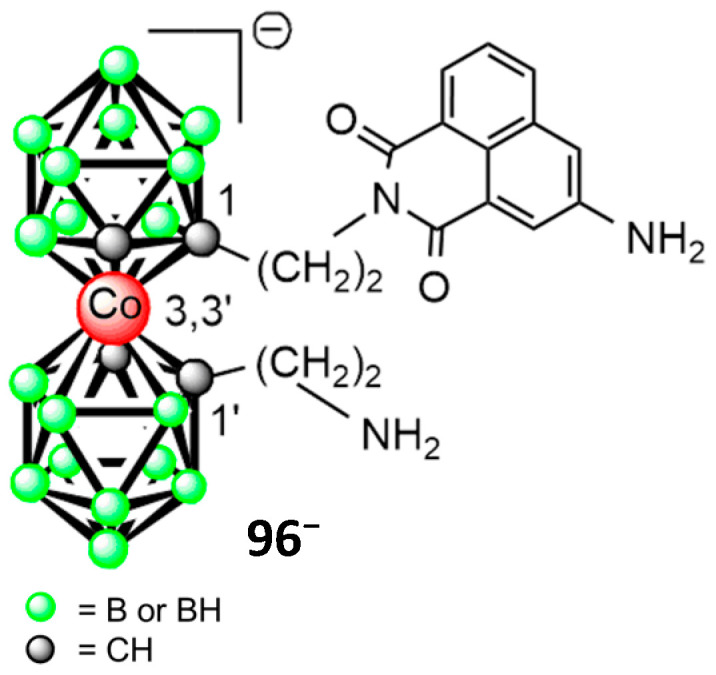
Schematic structure of the derivative **96^−^** showing the most promising antiproliferative activity.

1,8-Naphthalimides are compounds well known for their high antitumor activities with mechanisms proceeding via intercalation into DNA. Conjugates containing naphthalimide moiety attached to carbon and boron atoms of the cobalt bis(dicarbollide) ion, analogues of organic drugs mitonafide, were synthesized and studied, along with a panel of related carborane derivatives. The biological evaluations included in vitro cytotoxicity, type of cell death, cell cycle, and ROS production testing. The boron cluster compounds showed a significant effect on the proliferation of cancer cells, although a different kind of activity was observed compared to organic drug leads, mitonafide, and pinafide. The most promising derivative from this series corresponded to a disubstituted cobalt bis(dicarbollide) ion containing the C(1) naphthalimide group attached to the cage via an ethylene linker (**96^−^**, Figure 23) along with a second aminoethyl substituent on C(1′) atom. Compounds without this auxiliary substitution or those containing two naphthalimide groups in the same cage positions proved to decrease in activity. The compound **96^−^** was selected for detailed studies, which revealed cytotoxicity against HepG2 cells and the activation of cell apoptosis. The compound caused cell cycle arrest in HepG2 cells. Further investigations in HepG2 cells revealed that compound **96^−^** can also induce ROS generation, particularly mitochondrial ROS (mtROS), which was reflected by an increased 8-oxo-dG level in DNA. The interactions of new compounds with ct-DNA were also studied by CD spectra and melting temperature. The results confirmed the weak intercalation of boronated compounds into DNA, which is in contrast to the organic naphthalimide derivatives that usually show significant intercalating action [162].

#### 5.2.2. Antiparasitic Activity

**Figure 24:**
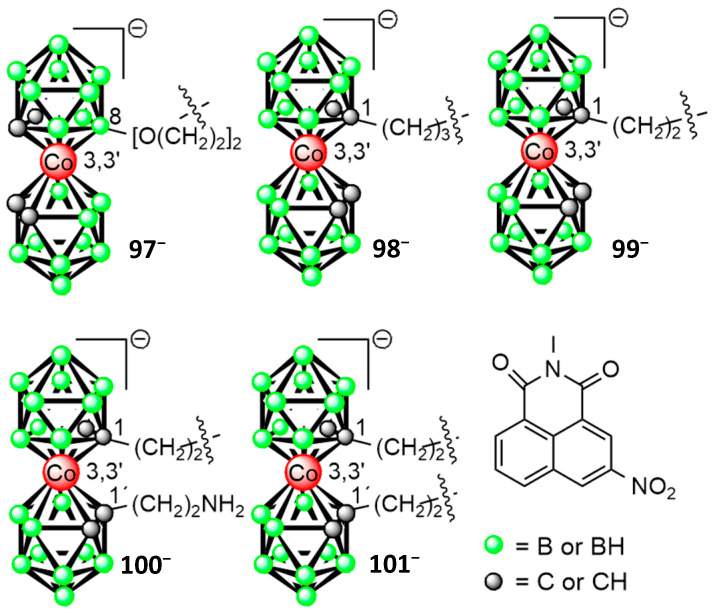
Chemical structures of [8-*R*-(1,2-C_2_B_9_H_10_)(1,2-C_2_B_9_H_11_)-3,3′-Co(III)] (R = (OC_2_H_4_)_2_ (**97^−^**), [1-*R*-(1,2-C_2_B_9_H_10_)(1,2-C_2_B_9_H_11_)-3,3′-Co(III)] (R = (CH_2_)_3_ (**98^−^**), (CH_2_)_2_ (**99^−^**)), [1-(CH_2_)_2_-2′-(NH_2_(CH_2_)_2_)-(1,2-C_2_B_9_H_10_)_2_-3,3′-Co(III)]^−^ (**100^−^**), and [1,1′-C_2_H_4_)_2_-(1,2-C_2_B_9_H_10_)_2_-3,3′-Co(III)]^−^ (**101^−^**) [56].

Boronated mitonafide analogues, naphthalimide derivatives of carboranes and metallacarborane Co(III) cages **97^−^**–**101^−^** (Figure 24), were prepared using reactions of C- and B-substituted amines with 3-nitro-1,8-naphthalic anhydride [56]. The biological tests of antihelmintic activity against Rhabditis sp. have shown that the cobalt bis(dicarbollide) conjugates **98^−^**, **99^−^**, attached via a carbon atom, showed the highest activity from the series. The lowest LC50 values and strongest nematicidal activity were observed for *N*-[(8-(3-oxa-pentoxy)-cobalt bis(dicarbollide)]-1,8-naphthalimide conjugate, in which the terminal group was attached via a bis(ethylenglycol) linker. Its LC_50_ value was as low as 0.148 mg mL^−1^. Slightly lower activity with an LC50 value of 0.207 mg mL^−1^ was observed for the carbon-bound conjugate corresponding to *N*-[2-cobalt bis(1,2-dicarbollide)ethyl)]-1,8-naphthalimide. Interestingly, compound **97^−^**, comprising a longer propylene linker between the modified group and the naphthalimide residue, exhibited significantly lower activity. These modified naphthalimide conjugates, **98^−^** and **99^−^**, displayed considerably higher activity than the mitonafide and mebendazole, drugs approved for the treatment of this type of parasitic infection.

### 5.3. Application of Boron-Substituted Compounds in Medicinal Chemistry

Comprehensive review articles about the potential of boron-substituted cobalt bis(dicarbollide) ions in medicinal chemistry and novel healthcare materials were published in 2023 by Teixidor et al. [150], 2022 by Chen et al. [187], and in 2022 by Das et al. [188]. Therefore, the following chapters will focus exclusively on selected recent articles, highlighting the overall use of cobalt bis(dicarbollide) as an unconventional pharmacophore in biomedicine.

#### 5.3.1. Antimicrobial Active Compounds and Antibiofilm Agents

The rapidly emerging resistance of bacterial pathogens to “classical” antibiotics presents a current and highly challenging issue in contemporary clinical practice. In this context, the use of unconventional hydrophobic pharmacophores based on boron clusters may potentially introduce a viable alternative. The possibility of using boron clusters in combating resistant forms of bacterial pathogens has been denoted by Plešek in his review published three decades ago [189]. His idea was based on an assumption that boron polyhedra are abiotic man-made compounds, which contain an unnatural type of bonding, and their surface composed of B-H bonds provides different interactions. From that time, knowledge about the mechanism of interactions of the cluster boron compounds with biological targets and cells increased appreciably. However, understanding the effects on the mechanism of resistance in particular types of pathogens, remains still limited. Some awareness of this idea can be seen in a recent paper published by Zheng et al. concerning the treatment of antibacterial infections [190] or a study by Řezáčová et al. and Kožíšek et al. showing that bis(dicarbollide) derivatives proved to have promising inhibitory action towards resistant mutants of viral HIV-Pr enzyme [31,159].

Considering antibacterial compounds, amines of the cobalt bis(dicarbollide) ion were recently proved to have antibiotic properties towards Gram-positive bacteria, in particular to Methicillin-resistant Staphylococcus aureus (MRSA), which is of high current concern. This is a significant pathogen that poses a threat to public health, particularly in healthcare settings. The emergence of drug-resistant strains has reduced the effectiveness of conventional treatments. This topic was recently covered by a review article published by Fink and Uchman [35]. Thus, here we focus only on several selected recent articles.

Microorganisms can exist in two forms: suspension cells or biofilms. Biofilms, which are communities of cells embedded in an extracellular matrix attached to a surface, can cause problematic infections. Biofilm formation often increases microbial resistance to antibiotics, necessitating the search for new antibiofilm compounds [191,192,193,194]. Cobalt bis(dicarbollide) and its derivatives have shown promising antimicrobial and pharmacological properties. While their antimicrobial activity has been studied, their antibiofilm activity remains largely unexplored [195,196,197,198]. Previous studies have primarily unlocked the antimicrobial activity of cobalt bis(dicarbollide) and its derivatives, focusing on Gram-positive bacteria and certain fungi, with limited efficacy observed against Gram-negative bacteria [196,197,198]. In the study by Vaňková et al., the antimicrobial and antibiofilm activities of cobalt bis(dicarbollide) derivatives (Na**1**, 8-NH_3_-**1**, and 8-PhNH_2_-**1** were investigated [199]. They showed effective antimicrobial properties against Gram-positive bacteria but limited activity against Gram-negative bacteria and Candida parapsilosis. All three compounds inhibited the growth of the filamentous fungus T. cutaneum. Na**1** exhibited strong antibiofilm activity against gram-positive bacteria, while 8-NH_3_-**1** and 8-PhNH_2_-**1** supported biofilm formation in P. aeruginosa. Na**1** disrupted the biofilm structure, inhibiting biofilm formation by at least 80%. Moreover, Na**1** had low cytotoxicity, making it a potential treatment for biofilm-associated infections. Further research is needed to explore their therapeutic applications [195].

**Scheme 20:**
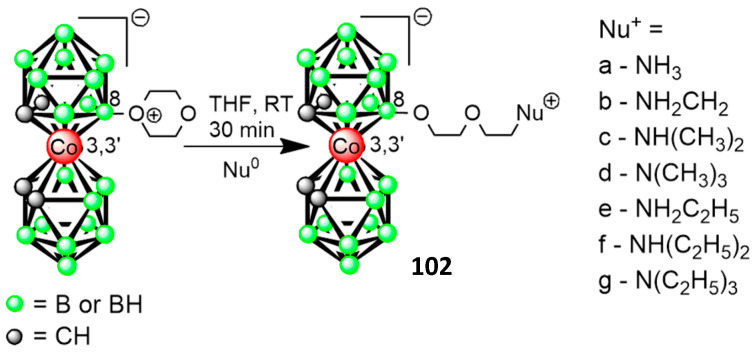
Preparation of [8-*Nu*(C_2_H_4_O)_2_-(1,2-C_2_B_9_H_10_)(1′,2′-C_2_B_9_H_11_)-3,3′-Co(III)] (**102**).

Popova et al. focused on the preparation, characterization, and antimicrobial properties of alkylammonium derivatives. The authors conducted the first investigation into the in vitro antimicrobial activity of compound **102** and its derivatives decorated with ethyl or salicylic ester groups (Scheme 20). They discovered that these derivatives possess antimicrobial activity similar to the antibiotic thiamphenicol against both bacterial and Candida spp. strains. Compound **102a** exhibited the non-selective growth inhibition of Gram-positive bacteria and fungi. Compound **102b** selectively inhibited Trichosporon cutaneum, while compound **102e** showed significant activity against filamentous fungi. Other derivatives were not effective. The increase in substituent bulkiness correlated with hydrophobicity and stability. The exact mechanism and selectivity remain unknown. Compounds **102a**, **102b**, and **102e** show promise as inhibitors of opportunistic pathogens [197].

**Scheme 21:**
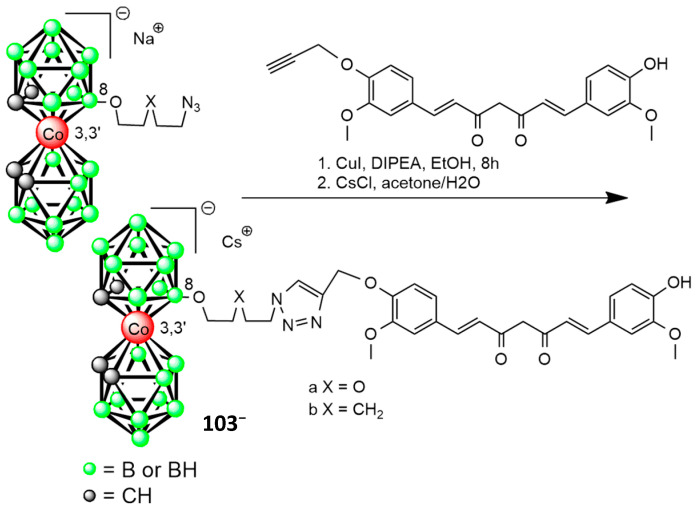
Preparation of [8-OC_4_H_8_*X*-4-((4-((1*E*,6*E*)-7-(4-hydroxy-3-methoxyphenyl)-3,5-dioxohepta-1,6-dien-1-yl)-2-methoxyphenoxy)methyl)-1H-1,2,3-triazole-(1,2-C_2_B_9_H_10_)(1′,2′-C_2_B_9_H_11_)-3,3′-Co(III)]^−^ (**103^−^**).

New conjugates of cobalt bis(dicarbollide) with curcumin were synthesized using the “click” methodology. The antibacterial activity of the synthesized curcumin derivatives was assessed. The compounds showed no activity against Gram-negative bacteria. However, variations in MIC values were observed for Gram-positive bacteria. Bacillus cereus was susceptible to all compounds, while Staphylococcus aureus and Enterococcus faecalis exhibited higher sensitivity to derivative **103e^−^**. Compound **103e^−^** also inhibited the growth of Aspergillus fumigatus, while the other samples had MIC values above 1000 mg L^−1^. In the presence of curcumin and derivatives **103a^−^** and **103b^−^** (Scheme 21)**,** a decrease in the growth density of Candida albicans was observed. Based on the results, compound **103b^−^** exhibited the highest activity against Gram-positive bacteria, A. fumigatus, and a decrease in the growth density of C. albicans, followed by curcumin, derivative **103a^−^**. These findings suggest the potential of these conjugates as antibacterial agents [200].

**Scheme 22:**
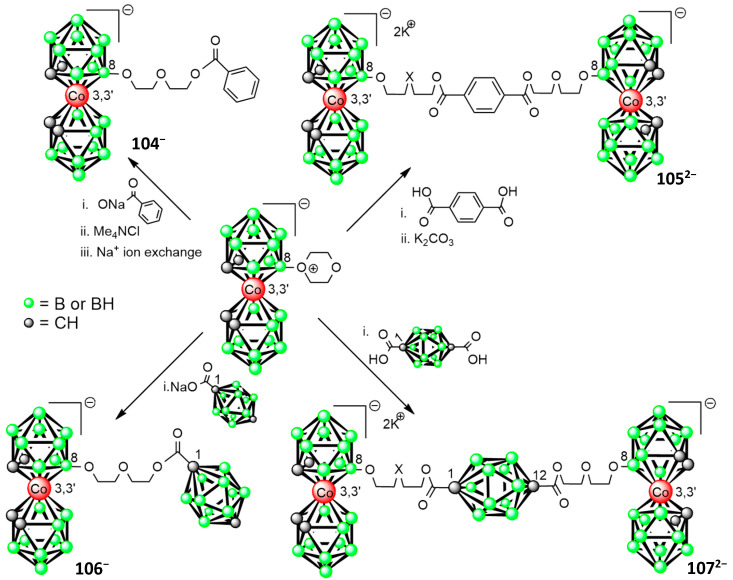
Reaction scheme of [8-O_2_C_4_H_8_-(1,2-C_2_B_9_H_10_)(1′,2′-C_2_B_9_H_11_)-3,3′-Co(III)] with sodium benzoate (**104^−^**), terephtalic acid (**105^2−^**), *p*-carborane-1-carboxylic acid (**106^−^**), and *p*-carborane-1,12-dicarboxylic acid (**107^2−^**).

In 2020, Romero et al. reported on the synthesis, characterization, and antimicrobial testing of cobalt bis(dicarbollide) derivatives Na**104**, K_2_**105**, Na**106**, and K_2_**107** (Scheme 22). The compounds were obtained through a ring-opening reaction of the cyclic oxonium zwitterion with various organic and inorganic carboxylates as nucleophiles. Microdilution tests revealed that the compounds lacked significant antibacterial effects on Gram-negative bacteria. However, they exhibited potent antibacterial activity against Gram-positive bacteria and had moderate antifungal activity against Candida albicans. The study assessed four Gram-positive bacteria strains, among them the life-threatening superbug MRSA, known for its resistance to various antimicrobial drugs. The cobalt bis(dicarbollide) derivative Na**104** displayed exceptional inhibitory effects on MRSA, with a standard minimum inhibitory concentration of 1 mg L^−1^ and a minimum bactericidal concentration of 2 mg L^−1^. These results suggest that Na**104** holds promise as a potent antibacterial agent against MRSA [201].

**Scheme 23:**
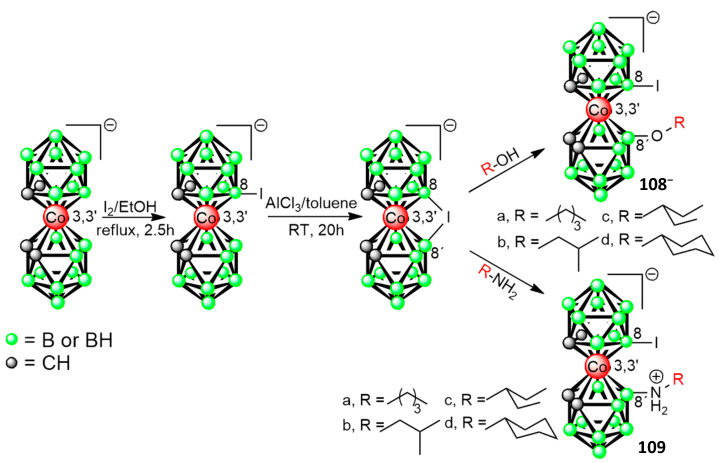
Synthetic road for preparation of [8-I-8′-O*R*-(1,2-C_2_B_9_H_10_)_2_-3,3′-Co(III)]^−^ (**108^−^**) and [8-I-8′-NH_2_*R*-(1,2-C_2_B_9_H_10_)_2_-3,3′-Co(III)] (**109**).

Swietnicki et al. (2021) conducted research on cobalt bis(dicarbollide) derivatives for antibacterial activity against Yersinia enterocolitica and Pseudomonas aeruginosa (Scheme 23). They utilized an iodonium bridge-opening reaction for synthesis, yielding predominantly O-linked derivatives. Compounds **1^−^**, **108^−^a**, **b**, **c**, **d**, and **109b** were most effective against Yersinia, with short aliphatic chains, being Pseudomonas, showing lower susceptibility. The compounds showed unique chemistry and induced resistance in Yersinia. They acted in a bacteriostatic manner, affecting cell division. *N*-linked boron derivatives were more potent in mammalian cell toxicity studies, and compound **108d^−^** had the lowest mortality rate in zebrafish toxicity tests at 20 μmol L^−1^ concentration [202].

**Scheme 24:**
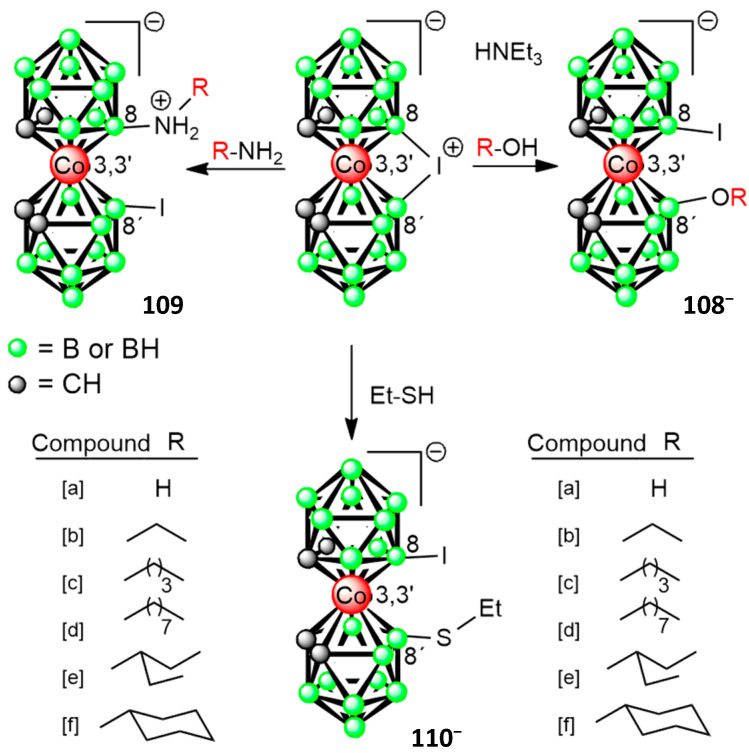
Synthesis of [8-NH_2_*R*-8′-I-(1,2-C_2_B_9_H_10_)_2_-3,3′-Co(III)] (**109**), [8-I-8′-O*R*-(1,2-C_2_B_9_H_10_)_2_-3,3′-Co(III)]^−^ (**108^−^**), and [8-I-8′-SEt-(1,2-C_2_B_9_H_10_)_2_-3,3′-Co(III)]^−^ (**110^−^**).

The synthesis of a series of cobalt bis(dicarbollide) derivatives containing an iodine atom at position B(8) and an organic substituent at B(8′) is described by Kubinśki et al. (2022). These derivatives fall into two types: anionic products linked through oxygen (B(8′)−O bond) and zwitterionic products linked through a nitrogen atom (B(8′)−N). The reactions involve the opening of the iodonium bridge with selected nucleophiles to yield desired bifunctional derivatives. The compounds’ antimicrobial activity was tested against Gram-positive and Gram-negative bacteria, as well as Candida albicans. The unmodified cobalt bis(dicarbollide) anion showed good activity against Gram-positive bacteria and certain fungi but not against Gram-negative bacteria. Synthesized metallacarborane derivatives displayed strong to moderate antimicrobial activity, comparable to some systemic drugs. Compounds **109a**, **109b**, and **109e** exhibited the strongest antibacterial properties (Scheme 24). They were also effective against Candida albicans, with the potential to overcome drug resistance. Some compounds showed synergy with amphotericin B. The compounds had low toxicity towards mammalian cells, making them promising for antiviral and anticancer therapies. Modifications to the parental compound improved safety in vivo [37].

**Figure 25:**
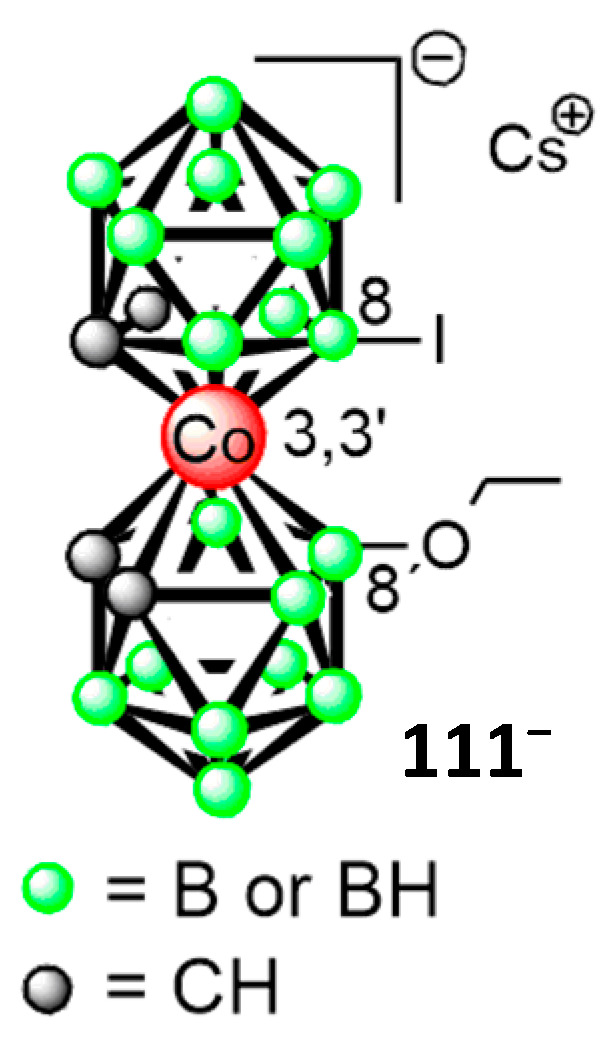
Chemical structure and [8-I-8′-OEt-(1,2-C_2_B_9_H_10_)_2_-3,3′-Co(III)]^−^ (**111^−^**).

To address the treatment of MRSA, Kosenko et al. and Zheng et al. described the synthesis [125] and antimicrobial properties of **111^−^**, a cobalt bis(1,2-dicarbollide) alkoxy derivative, as a potential therapeutic agent (Figure 25). Compound **111^−^** demonstrated potent anti-MRSA activity, effectively reducing the number of MRSA colonies and completely eradicating the bacteria at suitable concentrations. It showed specific activity against MRSA and did not significantly affect other drug-resistant bacteria. The concentration-dependent anti-MRSA effect of **111^−^** was observed, with the complete eradication of MRSA at a concentration as low as 8 μg mL^−1^. **111^−^** exhibited the fastest killing kinetics among the reported metallacarboranes. Importantly, multiple treatments with **111^−^** did not induce drug resistance in MRSA, unlike vancomycin. **111^−^** also showed excellent antibiofilm activity, inhibiting MRSA biofilm formation at sub-MIC concentrations. The mechanism of action of **111^−^** involved damaging the MRSA cell wall/membrane, which was confirmed using microscopy and staining techniques. The increase in reactive oxygen species (ROS) induced by **111^−^** contributed to cell membrane damage. **111^−^** exhibited excellent biocompatibility with mammalian cells and negligible cytotoxicity. These findings highlight the potential of **111^−^** as a nonantibiotic therapeutic agent for the treatment of MRSA infections [190].

**Scheme 25:**
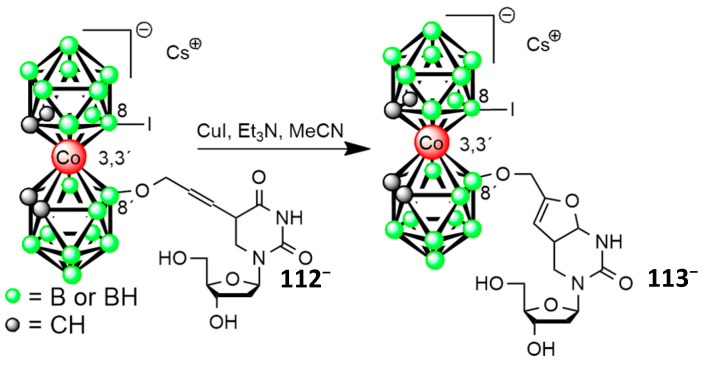
Reaction scheme of cobalt bis(dicarbollide) with 5-ethynyl-2′-deoxyuridine and further intramolecular cyclization (**113^−^**).

Kosenko et al. investigated an iodonium-bridged derivative of **1^−^** for its reactivity with nucleophilic reagents, such as alcohols and amines, leading to disubstituted derivatives. The reaction of the starting derivative with propargyl alcohol yielded terminal alkyne, which underwent a Sonogashira reaction with 5-iodo-2′deoxyuridine to form cobalt bis(dicarbollide) and 5-ethynyl-2′-deoxyuridine conjugate **112^−^** (Scheme 25). Further treatment of **112^−^** resulted in an intramolecular cyclization product, furo [2,3-*d*]pyrimidin-2(3*H*)-one conjugate **113^−^**. The cytotoxicity of **112^−^** and **113^−^** was moderate, with the fetal lung fibroblast MRC-5 cells being the most sensitive. The compounds did not show antiviral activity against tested DNA and RNA viruses. Another attempt to synthesize a 5-ethynyl-2′-deoxyuridine conjugate with a similar spacer length but with a compensating charge did not yield the desired product [203].

#### 5.3.2. Anticancer Compounds

##### Cholesterol-Containing Compounds for Anticancer Therapy

**Figure 26:**
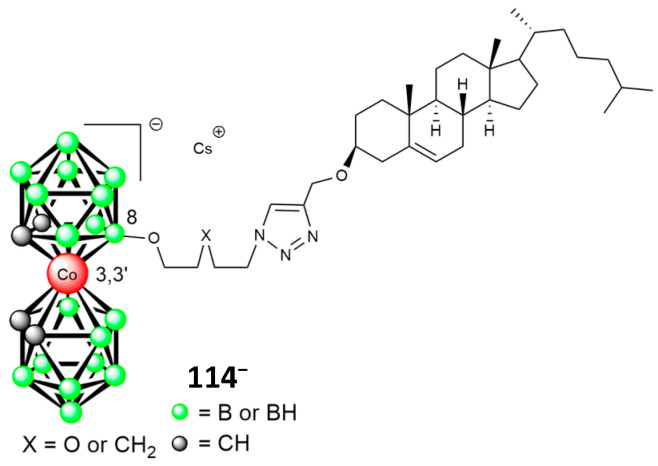
Chemical structure of [8-(OC_4_H_8_-*X*-N_3_(3*β*-(2-azido-ethyl)cholest-5-ene))-(1,2-C_2_B_9_H_10_)(1′,2′-C_2_B_9_H_11_)-3,3′-Co(III)]^−^ (**114^−^**).

Considering anticancer compounds, cholesterol-containing compounds have gained attention for anticancer therapy. Liposomes, known for their biocompatibility, biodegradability, and low immunogenicity, are widely used as nanocarriers for hydrophobic and hydrophilic molecules. They are already actively used in medicine for the transport of some anticancer drugs, such as doxorubicin [204] or paclitaxel [205]. Cancer cells require an increased demand for cholesterol to build their membranes, making boron-containing cholesterols a promising strategy for selective boron delivery using liposomes. Recently, cobalt bis(dicarbollide) conjugates with cholesterol have been synthesized, forming stable and non-toxic liposomes. The conjugates were prepared by performing a copper(I)-catalyzed cycloaddition reaction. Cobalt bis(dicarbollide) derivatives were used with terminal azido groups, which were then reacted with an alkyne compound. The resulting triazoles contained cobalt bis(dicarbollide) at position 1 and cholesterol at position 4. Compounds **114^−^** (Figure 26) were tested for their anti-cancer effects using an MTT assay on various cell lines, including MCF7, HCT116, A549, and WI38. The cytotoxicity of the compounds, compared to cisplatin, was assessed. They demonstrated low toxicity (IC50 > 200 μmol L^−1^) and exhibited consistent antiproliferative activity across different cell lines, irrespective of the spacer structure. This suggests their suitability for BNCT without significant toxicity concerns [206].

**Scheme 26:**
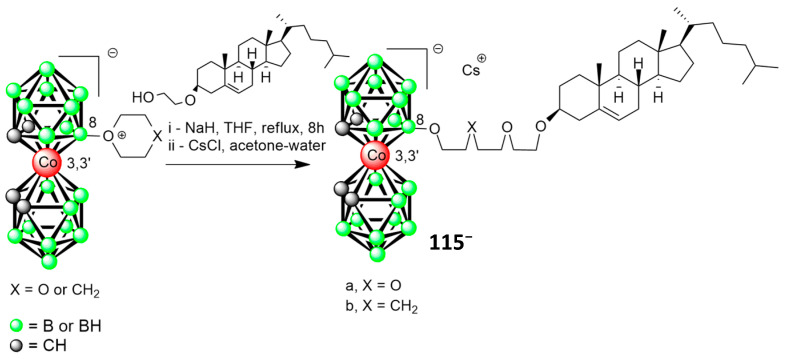
Preparation of [8-(O_2_C_4_H_8_-*X*-(3*β*-(2-azido-ethyl)cholest-5-ene))-(1,2-C_2_B_9_H_10_)(1′,2′-C_2_B_9_H_11_)-3,3′-Co(III)]^−^ (**115^−^**).

Druzina et al. synthesized cholesterol derivatives of cobalt bis(dicarbollide) with different spacers, namely hydrophilic (CH_2_CH_2_O)_2_ in compound **115a^−^** and lipophilic (CH_2_)_5_ in compound **115b^−^** (Scheme 26). These derivatives were obtained through nucleophilic ring-opening reactions using oxonium derivatives of cobalt bis(dicarbollide) and a modified cholesterol derivative. The cytotoxicity of compounds **115a^−^** and **115b^−^** was evaluated against glioblastoma cells (U-87 MG) and human embryo fibroblasts (FECH-15). Compound **115a^−^** showed lower cytotoxicity towards normal cells compared to tumor cells, with an IC50 value of 1.56 mg mL^−1^ for normal cells and 0.06 mg mL^−1^ for tumor cells. Compound **115b^−^** exhibited similar cytotoxicity towards both cells, with IC50 values of 39.5 mg mL^−1^ and 1.56 mg mL^−1^, respectively. The selective cytotoxicity index (CC50) for compounds **115a^−^** and **115b^−^** indicated their potential as antitumor agents. These cholesterol cobalt bis(dicarbollide) conjugates could be promising for BNCT and as independent antitumor agents. They also synthetized cholesterol-cobalt bis(dicarbollide) conjugates through the “click” cycloaddition of 3*β*-(2-azido-ethyl)cholest-5-ene and acetylene-functionalized cobalt bis(dicarbollides). These boronated cholesterols hold potential for application as drug delivery systems for Boron Neutron Capture Therapy in cancer treatment [164].

**Scheme 27:**
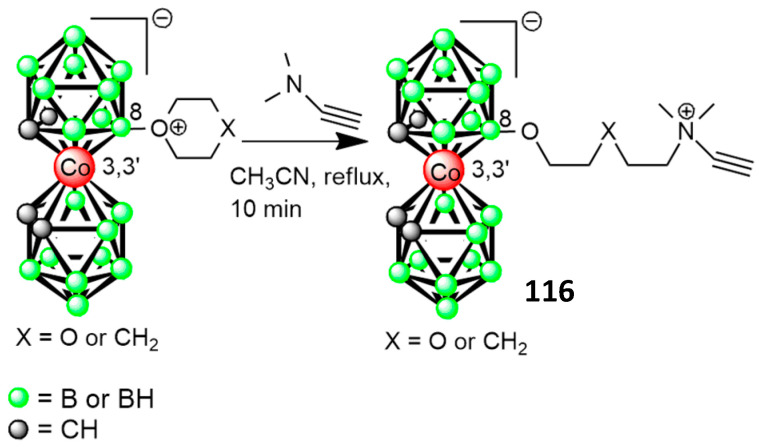
Preparation of [8-OC_4_H_8_-*X*-NC_4_H_7_-(1,2-C_2_B_9_H_10_)(1′,2′-C_2_B_9_H_11_)-3,3′-Co(III)] (**116**).

**Figure 27:**
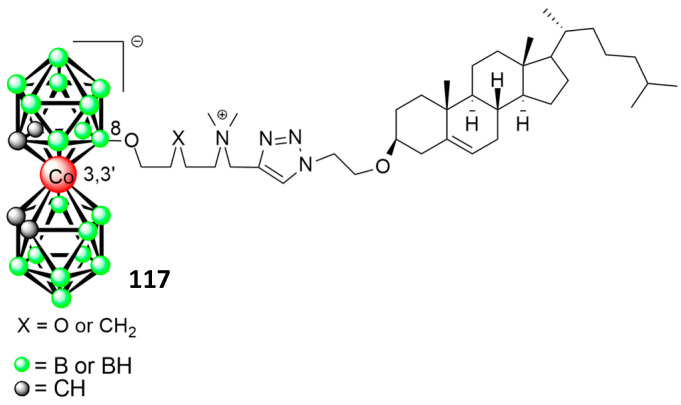
Chemical structure of [8-(OC_4_H_8_-*X*-NC_5_H_9_N_3_-(3*β*-(2-azido-ethyl)cholest-5-ene))-(1,2-C_2_B_9_H_10_)(1′,2′-C_2_B_9_H_11_)-3,3′-Co(III)] (**117**).

Furthermore, Druzina et al. reported on the synthesis of derivatives of cobalt bis(dicarbollide) terminal alkynes with charge-compensated groups (**116**; Scheme 27). They describe a reaction in which the oxonium derivative of cobalt bis(dicarbollide) reacts with tertiary amines to form corresponding ammonium salts. The authors successfully prepared terminal alkynes with quaternary ammonium groups based on cobalt bis(dicarbollide) using *N,N*-dimethylprop-2-yn-1-amine, resulting in novel derivatives with high yields. These compounds were further used in the synthesis of boronated cholesterol derivatives utilizing click reactions between terminal alkynes on metallacarborane and an organic azido-cholesterol derivative (**117**; Figure 27). The reactions yielded novel boron conjugates in high yields. The crystal structures of the alkynes prepared from 1,4-dioxane and tetrahydropyran derivatives of cobalt bis(dicarbollide) were determined. The synthesized compounds will be used to prepare boronated liposomes for delivering boron clusters to cancer cells in future BNCT experiments [108].

**Figure 28:**
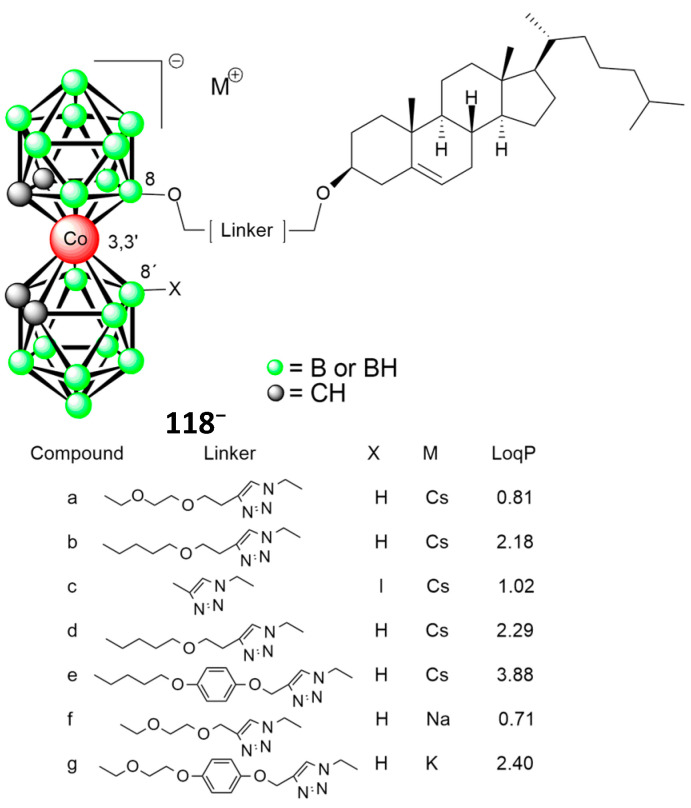
Chemical structure of [8-(OCH_2_-*Linker*-(3*β*-(2-azido-ethyl)cholest-5-ene))-(1,2-C_2_B_9_H_10_)(1′,2′-C_2_B_9_H_11_)-3,3′-Co(III)]^−^ (**118^−^**) [207].

The work of Dubey et al. (2021) explored the use of different linkers in liposomal formulations and their effects on various properties. The linkers tested include triazole, polyethylene glycol, benzene, and hydrocarbon chains (Figure 28). **118f^−^** is identified as the most hydrophilic, while **118e^−^** is the most hydrophobic among the compounds studied. The liposomal formulations are prepared using lipid thin-film hydration and extrusion, resulting in stable and uniformly sized particles with high zeta potential, indicating their physical stability. Encapsulation efficiency is found to be excellent for most formulations, with **118b^−^**, **118d^−^**, and **118f^−^** showing optimal hydrophobicity and the absence of benzene rings leading to high efficiency. The liposomes exhibit minimal drug release at physiological pH, suggesting sustained drug availability for tumor tissues through the enhanced permeability and retention (EPR) effect [207].

##### Curcumin-Containing Compounds for Anticancer Therapy

**Figure 29:**
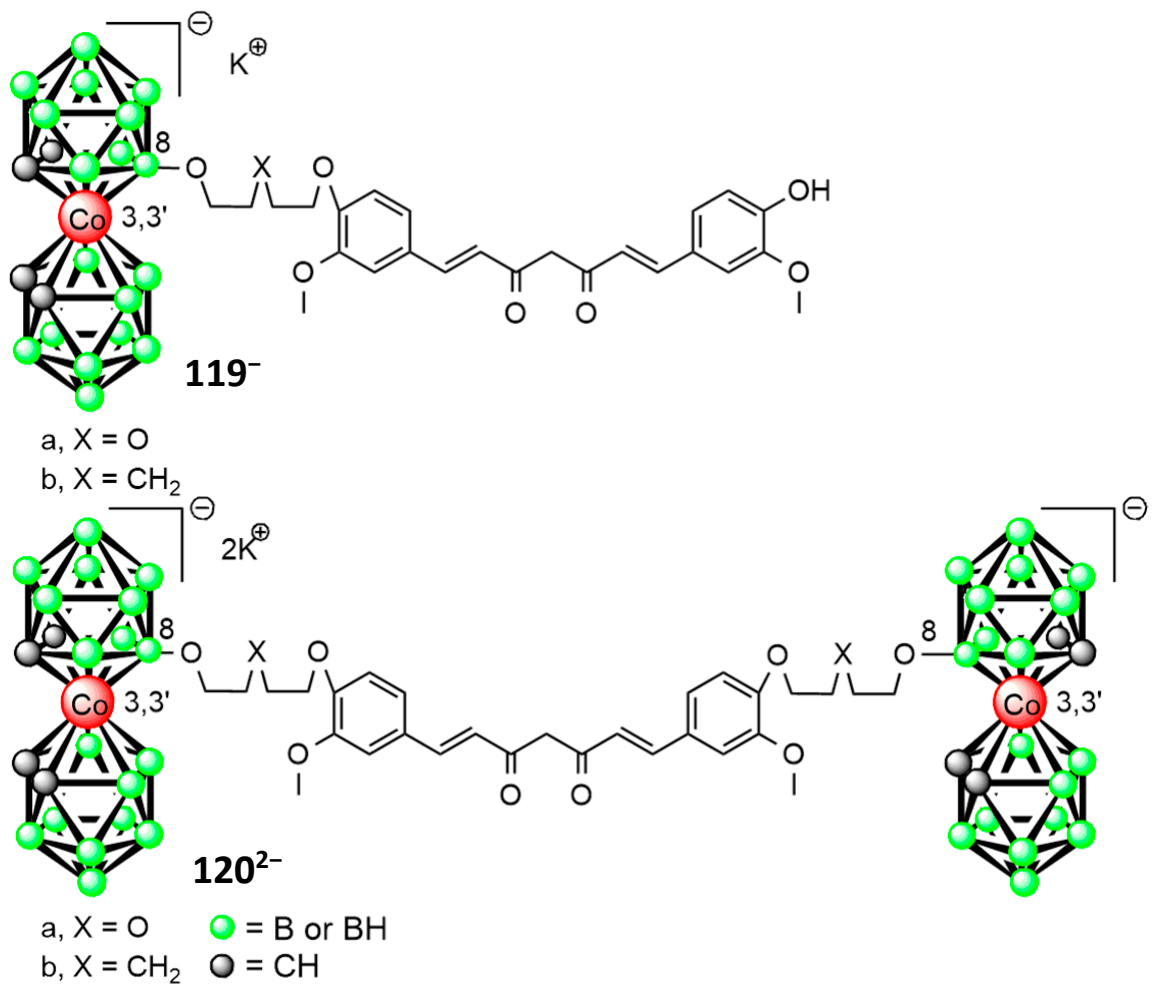
Chemical structure of [8-OC_2_H_4_-*X*-C_2_H_4_O-((1*E*,6*E*)-1-(4-hydroxy-3-methoxyphenyl)-7-(3-methoxyphenyl)hepta-1,6-diene-3,5-dione)-(1,2-C_2_B_9_H_10_)(1′,2′-C_2_B_9_H_11_)-3,3′-Co(III)]^−^ (**119^−^**) and {((1E,6E)-1,7-bis(3-methoxyphenyl)hepta-1,6-diene-3,5-dione)-[8-OC_2_H_4_-*X*-C_2_H_4_O-(1,2-C_2_B_9_H_10_)(1′,2′-C_2_B_9_H_11_)-3,3′-Co(III)]_2_}^2−^ (**120^2−^**).

In the synthesis of cobalt bis(dicarbollide)-curcumin conjugates, a series of compounds were designed using a “boron click” reaction. The reaction involved the nucleophilic ring-opening of cyclic oxonium derivatives of cobalt bis(dicarbollide) with the OH-group of curcumin. Different oxonium cycles and curcumin were used to obtain the boronated curcumin derivatives of cobalt bis(dicarbollide). Cyclic esters of cobalt bis(dicarbollide) provided spacers with varying hydrophilicity (–(CH_2_CH_2_O)_2_– spacer) or lipophilicity (–(CH2)4-5– spacer) between the boron cage and the biological macromolecule. The choice of spacers allowed for flexibility and biocompatibility in the synthesized compounds. Monoanionic and dianionic products were isolated as potassium salts with good yields. The viability of human tumor cell lines (HCT116 and K562) and non-malignant human skin fibroblasts (hFB-hTERT6) was assessed to evaluate the effect of prepared conjugates. Curcumin and doxorubicin were used as reference compounds. Curcumin exhibited cytotoxicity against all cell lines, with slightly lower toxicity towards normal fibroblasts compared to malignant cells. However, the cobalt bis(dicarbollide)-curcumin conjugates **119^−^a**, **b**, and **120^2−^a**, **b** were found to be inactive (Figure 29). To determine if the lack of cytotoxicity was due to poor cell penetration, intracellular drug accumulation was examined using flow cytometry. It was confirmed that all the conjugates entered HCT116 cells in a time-dependent manner. Conjugate **119a^−^** showed the highest penetration, while compounds **120a^2−^** and **120b^2−^** required longer incubation times for complete accumulation. The compounds did not affect cell viability. These findings indicate that compounds **119^−^a**, **b**, and **120^2−^a**, **b** can penetrate cells without causing cytotoxicity [109].

##### Chlorin-Containing Compounds for Anticancer Therapy

**Figure 30:**
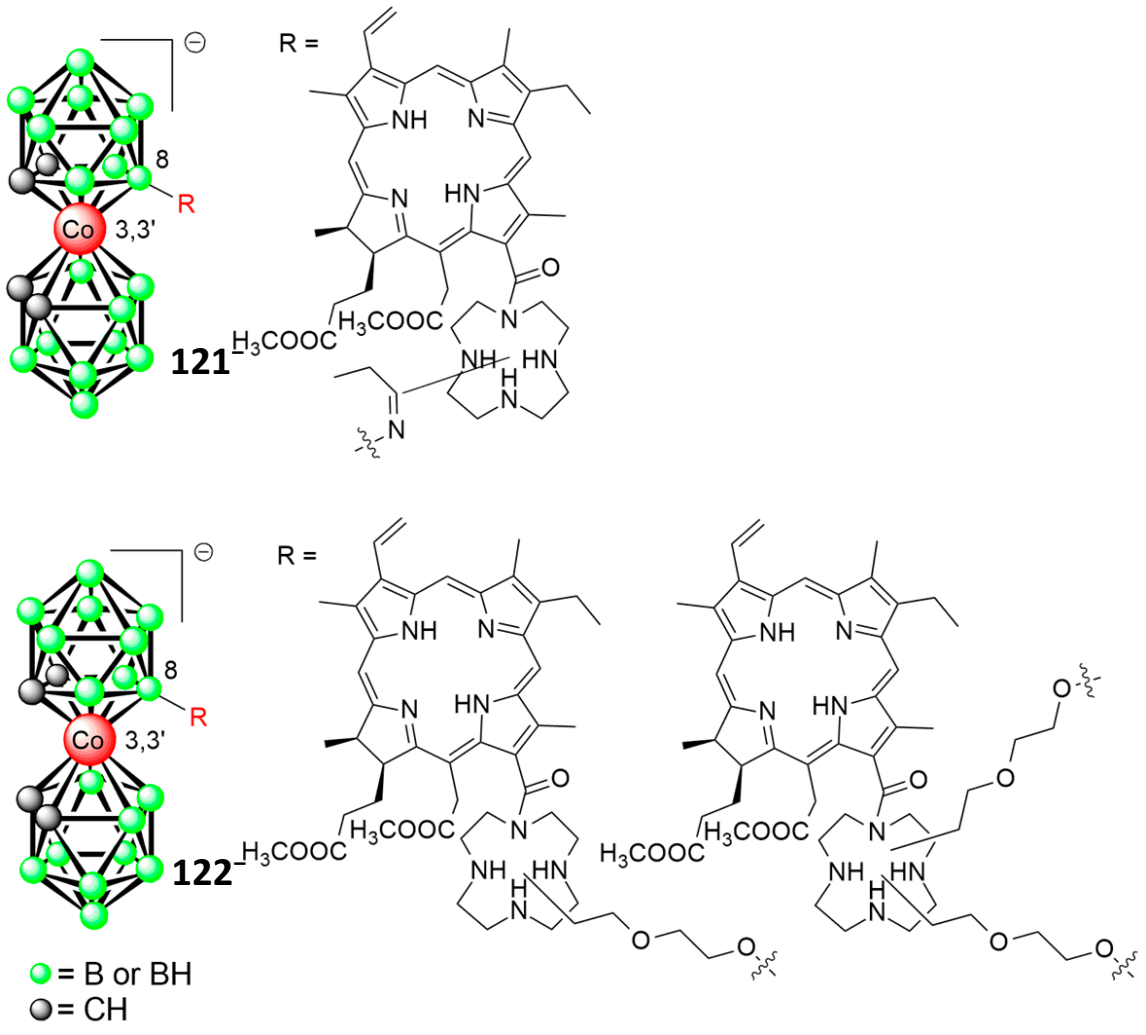
Chemical structure of chlorin–cyclen cobalt bis(dicarbollide) derivatives.

Determining boron content in blood and tissue samples is crucial for BNCT planning and application. Ex vivo methods, like inductively coupled plasma mass spectroscopy (ICP-MS), atomic emission spectrometry (ICP-AES) [109,208,209], and prompt gamma-ray spectroscopy (PGRS) [210,211,212], are commonly used for boron accumulation assessment. Fluorescent boron-containing compounds offer a non-invasive approach to the quantitative assessment of boron content in biological tissues, as their fluorescence intensity corresponds to the boron amount [213]. Chlorin *e*_6_-cobalt bis(dicarbollide) conjugates (CCDC) have shown selective accumulation in lung adenocarcinoma cells suitable for BNCT. However, accurately assessing the boron content using conjugate fluorescence remains a challenge [214,215]. To address this, mathematical simulations based on pharmacokinetic models were used to provide a preliminary assessment of medication doses and compound content in various organs. In the case of studying new boron-containing compounds for BNCT, the development of pharmacokinetic models is of significant interest [216]. The study of Volovetsky et al. demonstrates a direct correlation between boron concentration and CCDC fluorescence in different tissues, indicating the stability of the compound. The authors successfully achieved a contrasting accumulation of boron between tumor and muscle tissues using the chlorin *e*_6_-cobalt bis(dicarbollide) conjugate (**121^−^**; Figure 30, top). This highlights the potential of fluorescent methods for the non-invasive determination of boron content in living organisms. Furthermore, a mathematical model was developed that accurately describes the accumulation and distribution of CCDC in tissues. The model’s high fit with the experimental data suggests its reliability and usefulness in understanding and predicting the behavior of boron accumulation and distribution in various organs [217].

Fedotova et al. discussed the combination of BNCT and photodynamic therapy (PDT) for treating head and neck tumors [218]. BNCT involves using non-toxic isotopes ^10^B and thermal neutrons to target cancer cells, while PDT uses chlorine derivatives as photosensitizers to absorb light and induce therapy [215,219]. Researchers aim to create a theranostic conjugate by adding boron clusters to the chlorin–cyclen conjugate (**122^−^**; Figure 30, bottom), increasing boron atom concentration for more efficient BNCT [220]. The text describes two approaches for preparing boron-containing chlorin conjugates, which comprise a chlorin–cyclen conjugate that reacts with a nitrile derivative of bis(1,2-dicarbollide) cobalt, resulting in the addition of one boron cluster; or, in the second case, the chlorin–cyclen conjugate reacts with a dioxane derivative of bis(1,2-dicarbollide) cobalt, yielding a mixture of mono- and di-substituted derivatives. Interestingly, when using nitrile derivatives, only one product was obtained, while the dioxane derivative allowed for the introduction of a second boron cluster. This difference can be attributed to the steric effects and the length of the spacer group between the macrocycle and boron polyhedron [218].

##### Coumarin-Containing Compounds

**Figure 31:**
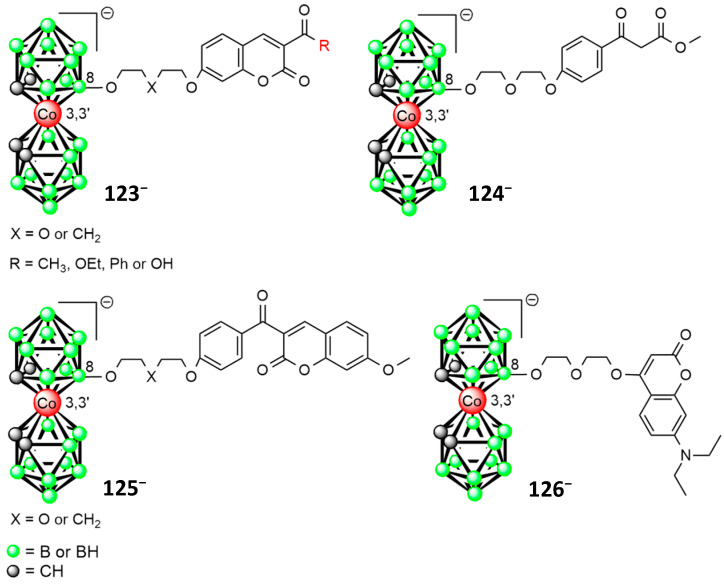
Chemical structure of [8-OC_2_H_4_-*X*-C_2_H_4_O-(2*H*-chromen-2-one)-CO-*R*-(1,2-C_2_B_9_H_10_)(1′,2′-C_2_B_9_H_11_)-3,3′-Co(III)]^−^ (**123^−^**), [8-O_3_C_4_H_8_-(methyl-3-oxo-3-phenylpropanoate)-(1,2-C_2_B_9_H_10_)(1′,2′-C_2_B_9_H_11_)-3,3′-Co(III)]^−^ (**124^−^**), [8-OC_2_H_4_-*X*-C_2_H_4_O-(3-benzoyl-7-methoxy-2*H*-chromen-2-one)-(1,2-C_2_B_9_H_10_)(1′,2′-C_2_B_9_H_11_)-3,3′-Co(III)]^−^ (**125^−^**), and [8-O_3_C_4_H_8_-(7-(diethylamino)-2*H*-chromen-2-one)(1,2-C_2_B_9_H_10_)(1′,2′-C_2_B_9_H_11_)-3,3′-Co(III)]^−^ (**126^−^**).

Coumarins are natural biologically active compounds with versatile biomedical applications (e.g., anticancer, antimicrobial, and anticoagulant activity). That is why they are given increased attention [221]. In 2020, Kosenko et al. performed a synthesis of coumarin derivatives attached to cobalt bis(dicarbollide) (**123^−^**, **124^−^**, **125^−^**; Figure 31). The authors used nucleophilic ring cleavage reactions with oxonium derivatives to prepare these compounds. They found that reactions with cobalt bis(dicarbollide) resulted in conjugates attached at either the C-3 or C-7 positions of coumarin. The new compounds showed good yields and fluorescent properties. The authors also attempted to attach the boron cluster at other positions of the coumarin ring, which has been found to be challenging [222]. Serdyukov et al. (2021) reported on a coumarin conjugate with cobalt bis(dicarbollide) as well (**126^−^**; Figure 31). The synthesis involved the ring cleavage of cyclic oxonium derivative of cobalt bis(dicarbollide) ion by coumarin moiety. The reaction of charge-compensated cobalt bis(dicarbollide) derivative with 7-diethylamino-4-hydroxycoumarin resulted in an anionic product. A new anionic boron conjugate **126^−^** was obtained in the form of a potassium salt. The lipophilicity of the synthesized compound was determined using a log*D*_7.4_ measurement, indicating that this conjugate seems promising for medicinal applications [223].

##### Glioblastoma and Neuroblastoma

Glioblastoma (GBM) is a highly aggressive primary brain tumor with limited treatment options and a poor prognosis. Standard treatments, such as surgical resection followed by chemoradiotherapy, are met with limited success due to the development of therapy resistance and tumor recurrence. Over the years, BNCT has been considered as a potential alternative for the treatment of GBM [224,225,226,227,228]. To further explore the potential of **1^−^** for BNCT, Serdyukov et al. employed Synchrotron Radiation-Fourier Transform Infrared (SR-FTIRM) micro-spectroscopy. This advanced technique allows for the non-destructive analysis of key molecular structures within cells, providing valuable diagnostic information. By utilizing the characteristic ν B-H frequency range of 2.600–2.500 cm^−1^, specific to boron clusters, SR-FTIRM enabled the detection and localization of **1^−^** within cells [223]. The study of Nuez-Martinez et al. investigates the uptake and effects of Na**1** in glioma-initiating cells (GICs). It demonstrates that Na**1** enters GICs and induces changes in cell phenotype, particularly in radio-resistant mesenchymal cells. The study highlights the potential of Na**1** as a therapeutic compound for glioblastoma treatment, especially in resistant cases of GBM. The use of SR-FTIRM provided insight into drug distribution and radio-resistance. The high uptake and rapid clearance of Na**1** make it a promising candidate for BNCT. Further research is needed to understand the underlying mechanisms and optimize its delivery [224].

In another study by Nuez-Martinez et al., the potential of **1^−^** for both chemotherapy and BNCT in GBM was investigated using in vitro and in vivo models. Studies using spheroids derived from the U87 and T98G cell lines revealed that T98G spheroids showed increased resistance to treatment compared to that of the U87 type, contrary to results observed in 2D monolayer cultures. This highlights the importance of employing 3D models for GBM studies. In vitro tests demonstrated that **1^−^** and [8,8′-I_2_-**1^−^**], at non-cytotoxic concentrations, effectively loaded sufficient levels of boron into GBM cells for successful boron neutron capture (BNC) reactions. T98G cells, known for their resistance to standard radiotherapies, exhibited enhanced sensitivity to neutron irradiation after incubation with Na[8,8′-I_2_-**1^−^**] due to their higher boron uptake. In vivo tests using C. elegans nematodes and embryos confirmed the toxicity of these compounds. The compounds formed hybrids with C. elegans’ eggs and arrested larvae development. Thus, [8,8′-I_2_-**1^−^**] and related compounds have the potential as candidates for the combination BNCT treatment of resistant variants of GBM [38].

**Figure 32:**
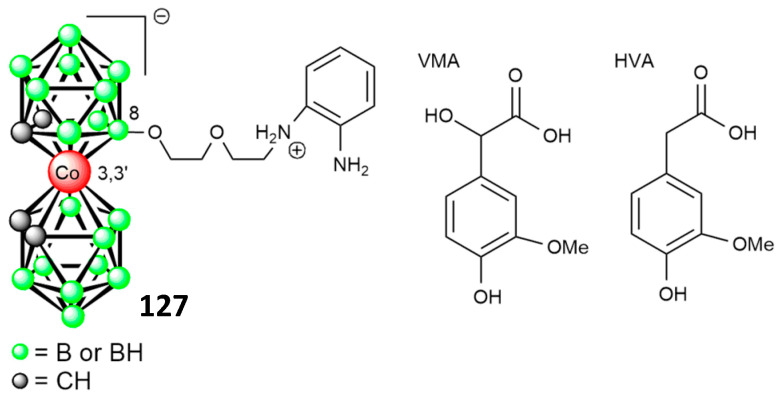
Chemical structure of [8-C_4_H_8_O_2_NH_2_-C_6_H_6_N-I-(1,2-C_2_B_9_H_10_)(1′,2′-C_2_B_9_H_11_)-3,3′-Co(III)] (**127**).

The neuroblastoma (NB) is the most common solid extracranial tumor in children [229]. Current diagnosis involves invasive procedures and general anesthesia for young patients. Screening for NB can be based on monitoring the levels of catecholamine metabolites, homovanillic acid (HVA), and vanillylmandelic acid (VMA) (Figure 32). Various laboratory techniques are used for their analysis. Immobilized supramolecular systems are proposed to enhance the detection of NB metabolites through selective interactions. In this study, Shishanova et al. designed a cobalt bis(dicarbollide) derivative including an o-phenylenediamine unit (CB-oPD) that shows potential for specific interactions with carboxylate analytes (**127**; Figure 32). The objective was to evaluate electrochemical techniques for determining VMA and HVA, considering their structural similarity. Electropolymerized film based on CB-oPD is proposed as a “host” for the selective detection of the metabolites (“guests”). The focus is on monitoring VMA and HVA as individual species and as a mixture to aid in correct diagnosis. This approach is considered novel for the electrochemical detection of these metabolites [230,231,232,233,234]. The electrochemical recognition of neuroblastoma metabolites was investigated on a modified electrode surface using electrochemical impedance spectroscopy (EIS), potentiometry, and differential pulse voltammetry (DPV). The association constants indicate the higher affinity of the electrode surface for HVA (2.5 × 10^5^) compared to VMA (4.3 × 10^4^). The binding of the metabolites to the pCB-oPD-modified electrode allows for the differentiation of VMA and HVA in a mixture, offering practical applications [234].

#### 5.3.3. Nanocomposites for Bioimaging and Drug Delivery

**Figure 33:**
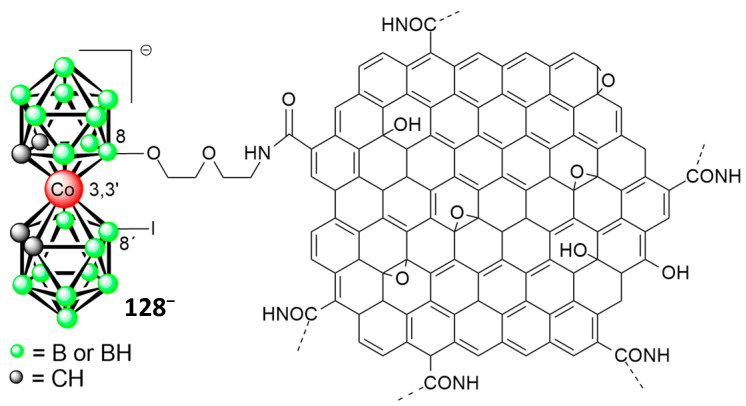
Chemical structure of [8-C_4_H_8_O_2_NH-*GO*-8′-I-(1,2-C_2_B_9_H_10_)_2_-3,3′-Co(III)]^−^ (**128^−^**).

Nanomaterials have shown great potential in the development of efficient in vivo imaging probes for noninvasive techniques like positron emission tomography (PET), magnetic resonance imaging, and optical imaging [234]. Carbon nanomaterials, such as graphene oxide (GO), have attracted attention due to their unique properties, high biocompatibility, and prolonged blood circulation times. GO can be functionalized and used as a platform for drug delivery systems, including anticancer agents [235,236,237]. The cobalt bis(dicarbollide) derivatives have been linked to macromolecules and nanoparticles, including GO, resulting in materials with improved properties and cellular uptake. The chemical modification of GO with cobalt bis(dicarbollide) anions has led to hybrid materials with improved dispersibility and thermal properties. Radiolabeling cobalt bis(dicarbollide) enables its visualization in vivo using PET. In the study of Ferrer-Ugalde et al., boron-enriched carbon-based materials tagged with the positron emitter iodine-124 (^124^I) were developed as theranostic agents for whole-body imaging. The nanocomposite, **128^−^** (Figure 33), was prepared by functionalizing graphene oxide (GO) with a monoiodinated boron-cluster compound (I-**1^−^**). To synthesize I-**1^−^**, iodine crystals were added to a solution of [8-C_4_H_8_O_2_NH_3_-3,3′-Co(1,2-C_2_B_9_H_10_)(1′,2′-C_2_B_9_H_11_)] in dichloromethane. To attach I-**1^−^** to GO, a covalent coupling reaction was performed using *N,N*′-dicyclohexylcarbodiimide (DCC) and 1-hydroxybenzotriazole (HOBt) as coupling reagents. The resulting **128^−^** exhibited exfoliated graphene derivatives and a graphitic structure. The biocompatibility of **128^−^** was evaluated in healthy cells using cytotoxicity studies. The results showed low cytotoxicity, with cell viability above 90% at different concentrations and incubation times. A TEM analysis confirmed the internalization of **128^−^**, and live/dead tests confirmed that the majority of cells remained alive, even at the highest concentration. The positive control exhibited high cell mortality. The TEM analysis showed the presence of material aggregates in the cytoplasm without affecting cell structure. In vivo toxicity tests using C. elegans demonstrated the lack of toxicity and internalization of both **128^−^** and I-**1^−^**. The radiolabeling of I-**1^−^** with iodine isotopes enabled PET imaging, revealing the accumulation of [^124^I] **128^−^** in the liver, lungs, and heart, with long circulation time and elimination through the gastrointestinal tract. These results indicate the potential of **128^−^** as a theranostic agent for Boron Neutron Capture Therapy [238].

Pulagam et al. (2021) developed PEG–cobalt bis(dicarbollide)-AuNRs, a nanoconjugate with potential applications in BNCT. They prepared CTAB-stabilized gold nanorods (AuNRs) and modified them with mPEG (poly(ethylene glycol) methyl ether thiol) for biocompatibility and circulation enhancement. Cobalt bis(dicarbollide) derivatives were then conjugated onto the AuNR surface. The presence of cobalt bis(dicarbollide) ion on the nanoconjugates was confirmed through various analyses, including NMR, STEM-EDXS, and XPS. Despite gold’s high cross-section for thermal neutrons, their calculations showed that the presence of gold as a boron carrier is not a limitation for BNCT applications. The nanoconjugates showed good stability, low cytotoxicity, and accumulated in the tumor in a mouse model of gastric adenocarcinoma. They exhibited promising results for combined Photothermal Therapy and BNCT in vitro, inducing localized thermal heating and cell damage under NIR and neutron irradiation. However, the accumulation of nanoconjugates in the tumor needs improvement for more effective BNCT treatment [239].

### 5.4. Electrochemistry

The electrochemistry of cobalt bis(dicarbollide) anion in non-aqueous media has already been reported in the early 1970s [64,240] and served predominantly as a characterization method for establishing the physicochemical properties of newly synthetized species. These works include, for example, studies on the B-substituted porphyrin derivatives and their spectroelectrochemistry [241], the electrochemical and spectroelectrochemical characterization of the phthalocyanines B-substituted potential BNCT treatment candidate [242], and the electrochemistry of different substituted metallacarboranes including the cobalt ones [243]. This progress was covered by a recent review article [11].

**Figure 34:**
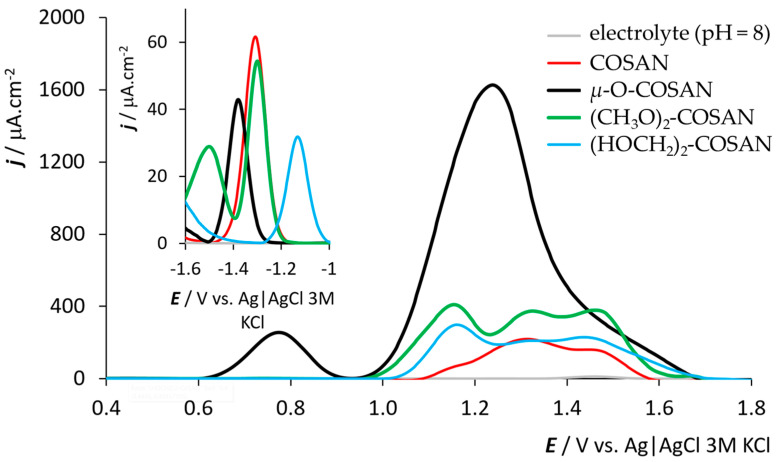
Example of DPV (differential pulse voltammogram) of some cobalt bis(dicarbollide) (COSAN) anions in phosphate buffer, pH = 8500 µmol L^−1^ concentrations of all samples, polished glassy carbon electrode. COSAN-*closo*-[(1,2-C_2_B_9_H_11_)_2_-3,3′-Co(III)]Cs; *µ*-O-COSAN–[*µ*-8,8′-O-(*ortho*-1,2-*closo*-C_2_B_9_H_10_)_2_-3,3′-Co(III)]Na.nH_2_O; (CH_3_O)_2_-COSAN–[8,8′-(CH_3_O)_2_-(*ortho*-1,2-*closo*-C_2_B_9_H_10_)_2_-3,3′-Co(III)]Me_4_N; and (HOCH_2_)_2_-COSAN–[1,1′-(HOCH_2_)_2_-1,2-C_2_B_9_H_10_)_2_-3,3′-Co(III)]Me_3_NH [63,244].

Growing attention to the use of the cobalt bis(dicarbollide) anion in biological applications led to the first attempts of electrochemistry in the aqueous milieu [11,12,14,153]. The authors report not only the reversible redox signal of the Co(III) central atom (as was reported previously in non-aqueous media [240]) but also the irreversible signal of the core boron cage (Figure 34) [63,82,244]. The electrochemical response for the parent cobalt bis(dicarbollide) anion (using a polished glassy carbon electrode in a phosphate buffer of pH = 8) is rather complicated. It consists of the reversible signal of the Co(III)/Co(II) redox process on the cathodic part of the voltammogram. On the anodic part, a set of at least three overlapping peaks could be observed in the bride potential range. This signal was ascribed to the electrooxidation of the dicarbollide ligands. Interestingly, in the case of the bridged samples, the set of overlapping peaks merged into one with a markedly higher current density response. This happens only in the case of a short bridge linker (as a single atom); with prolonging the linker chain, the signals tend to split again into a set of overlapping peaks [244]. This behavior could be explained by the presence of the three main rotamers of the parent cobalt bis(dicarbollide) anion, where the short bridging linker does not allow the transition between the different states. Study [63] compares the electrochemical behavior of B- and C-substituted derivatives, namely the alkylhydroxy and carboxy groups. Although the electronic properties of the C- and B-derivatives (with comparable ligand structure) markedly differ, this work does not find any significant difference between the electrochemical behavior of these derivatives. Interestingly, other studies conducted in aqueous media reported only the reversible signal of Co(II), for example, the original electrochemical work of the Teixidor group [12], and the use of bis(dicarbollide) ion as a transducer on an electrode for neuroblastoma markers sensing [245].

## 6. Conclusions and Outlook

This review particularly and primarily focuses on the chemistry of the carbon-substituted derivatives of the cobalt bis(dicarbollide) cage, which proceeds via the direct metalation of C-H vertices followed by reactions with electrophiles. We have demonstrated that these methods can be used as a versatile solution for producing a large variety of available structural blocks that offer viable alternatives to boron-substituted compounds and can be used in diverse applications. As demonstrated, due to its flexibility, this approach already resulted in improved inhibitory properties of enzyme inhibitors designed to target cancer-associated CA-IX or compounds that proved to have better antiparasitic activity than the boron-substituted analogues. Furthermore, the methods outlined here also allow for an easy heterosubstitution on boron and carbon vertices.

In light of recent advancements in ferrocene chemistry, we can anticipate further progress in the understanding of the mechanism of metalation of the cobalt bis(dicarbollide) cage. This progress will likely be accompanied by further improvements in reaction conditions, including the use of auxiliary bases and reagents, aimed at enhancing the stereospecificity of these reactions. It is worth investigating the *ortho*-directive effect of certain groups located on carbons and the activation of B-H bonds in close proximity.

The chemistry of carbon atoms provides a straightforward means of inducing chirality into molecules. These compounds may, in principle, serve as chiral platforms resembling BINOL, BINAP, and chiral ferrocenes. Progress in the chemistry of chiral species may, thus, be anticipated, leading to the production of enantiomers of high enantiomeric excess when chiral additives are used in metalation reactions. This will be paralleled with the further development of analytical (and preparative) HPLC and other separation methods that would allow for the fast analysis of enantiomeric purity.

## Figures and Tables

**Table 1:** In vitro inhibition of selected carbonic anhydrase isoenzymes [75].

Compound	Linker, Stereochemistry or Substitution	*K*_i_ (CA-II) [nM]	*K*_i_ (CA-IX) [nM]	Selectivity Index [a]
**95^−^1**	Mono, C_2_	133.10 ± 15.35	0.92 ± 0.28	144.2
**91a^−^**	Mono, C_2_, *Cl*_2_	29.85 ± 4.46	0.89 ± 0.15	33.5
**91b^−^**	Mono, C_3_, *Cl*_2_	8.10 ± 0.86	0.10 ± 0.02	82.7
**93a^−^**	C_1_, *rac*-	41.09 ± 6.09	0.86 ± 0.12	47.6
**93b^−^**	C_2_, *rac*-	164.90 ± 19.86	0.86 ± 0.15	191.5
**94a^−^**	C_2_, *meso*-	74.17 ± 12.67	0.37 ± 0.05	199.9
**93c^−^**	C_3_, *rac*-	11.36 ± 1.25	0.02 ± 0.003	668.2
**94b^−^**	C_3_, *meso*-	26.76 ± 2.45	0.60 ± 0.12	44.5
**93d^−^**	C_4_, *rac*-	10.07 ± 0.61	0.29 ± 0.01	35.2
**94c^−^**	C_4_, *meso*-	31.57 ± 3.69	0.06 ± 0.01	574.0

[a] Selectivity index is the ratio between *K*_i_ (CA-II) and *K*_i_ (CA-IX).
